# Relating Conformational Equilibria to Conformer‐Specific Lipophilicities: New Opportunities in Drug Discovery

**DOI:** 10.1002/anie.202114862

**Published:** 2021-12-29

**Authors:** Bruno Linclau, Zhong Wang, Benjamin Jeffries, Jérôme Graton, Rodrigo J. Carbajo, Davy Sinnaeve, Jean‐Yves Le Questel, James S. Scott, Elisabetta Chiarparin

**Affiliations:** ^1^ School of Chemistry University of Southampton Highfield, Southampton SO17 1BJ UK; ^2^ Department of Organic and Macromolecular Chemistry, Ghent University Campus Sterre, S4 Krijgslaan 281 9000 Ghent Belgium; ^3^ CEISAM UMR CNRS 6230 Université de Nantes CNRS CEISAM UMR 6230 44000 Nantes France; ^4^ Medicinal Chemistry, Oncology R&D AstraZeneca Cambridge CB4 0WG UK; ^5^ Univ. Lille Inserm Institut Pasteur de Lille CHU Lille U1167—RID-AGE—Risk Factors and Molecular Determinants of Aging-Related Diseases 59000 Lille France; ^6^ CNRS ERL9002—Integrative Structural Biology 59000 Lille France

**Keywords:** Amides, Conformation, Drug Development, Lipophilicity, NMR Spectroscopy

## Abstract

Efficient drug discovery is based on a concerted effort in optimizing bioactivity and compound properties such as lipophilicity, and is guided by efficiency metrics that reflect both aspects. While conformation–activity relationships and ligand conformational control are known strategies to improve bioactivity, the use of conformer‐specific lipophilicities (log*p*) is much less explored. Here we show how conformer‐specific log*p* values can be obtained from knowledge of the macroscopic log*P* value, and of the equilibrium constants between the individual species in water and in octanol. This is illustrated with fluorinated amide rotamers, with integration of rotamer ^19^F NMR signals as a facile, direct method to obtain log*p* values. The difference between log*p* and log*P* optimization is highlighted, giving rise to a novel avenue for lipophilicity control in drug discovery.

## Graphical abstract

Catching conformations: Lipophilicity (log*P*) is a molecular property which is nevertheless the weighted average of the lipophilicities of the individual conformers. The first direct measurement of conformer‐specific lipophilicity values is presented, together with the introduction of a new avenue for rational lipophilicity optimization of drug candidates which is focused on modification of conformational equilibria in water and in octanol.

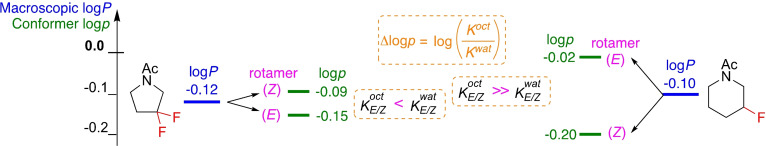

In drug development, the focus on bioactivity has its fundamental origin in an easily understood inverse relationship between bioactivity and dose. In addition, the lower the dose, the lower the risk of toxicity and/or side effects stemming from non‐specific off target activity.^[1]^ Another crucial determinant of drug dosing is lipophilicity (log*P*), as a proxy for a host of physical parameters related to ADMET (absorption, distribution, metabolism, excretion and toxicity).^[2]^ Optimal compound lipophilicity ranges have been proposed. It can be said that historically this aspect has been—at best—deemed secondary compared to the quest for bioactivity, resulting in worrying drug attrition rates in costly late‐stage clinical trials.^[3]^ However, much effort is currently devoted to understanding structure‐lipophilicity relationships including the development of tools to modify lipophilicity. Fluorine introduction is one of the possible strategies for this purpose.^[4]^ A concerted effort towards bioactivity optimization and lipophilicity control is now regarded as the best strategy for successful drug discovery.^[5]^ Novel efficiency metrics reflecting both aspects have been introduced,[^3^, ^6^] and further insights and developments aiding this interactive process are of high interest.

The benefits of considering molecular conformational preferences when optimising ligand‐protein interactions have been well‐recognised,^[7]^ and many approaches have been studied that stabilise or even lock a flexible molecule into its bioactive conformation, in order to reduce the entropic penalty of binding. Fluorination has a notable role in this area as well.[^4a^, ^8^]

Lipophilicity is a molecular property (a log*D*_pH_ value reflects the protonation state of ionisable species), and is defined as the concentration ratio of the solute in the octanol over the water phase ((Eq. 1), Figure 1A). This is referred to as macroscopic lipophilicity (log*P*). However, molecular properties result from the combination of the properties of individual conformers in solution,^[9]^ and the concept of conformer‐specific partition coefficients (log*p*) has been introduced by Davies et al in 1979.^[10]^ These are defined as the concentration ratio of a given conformer over the two phases ((Eq. 2), Figure 1A).

**Figure 1:**
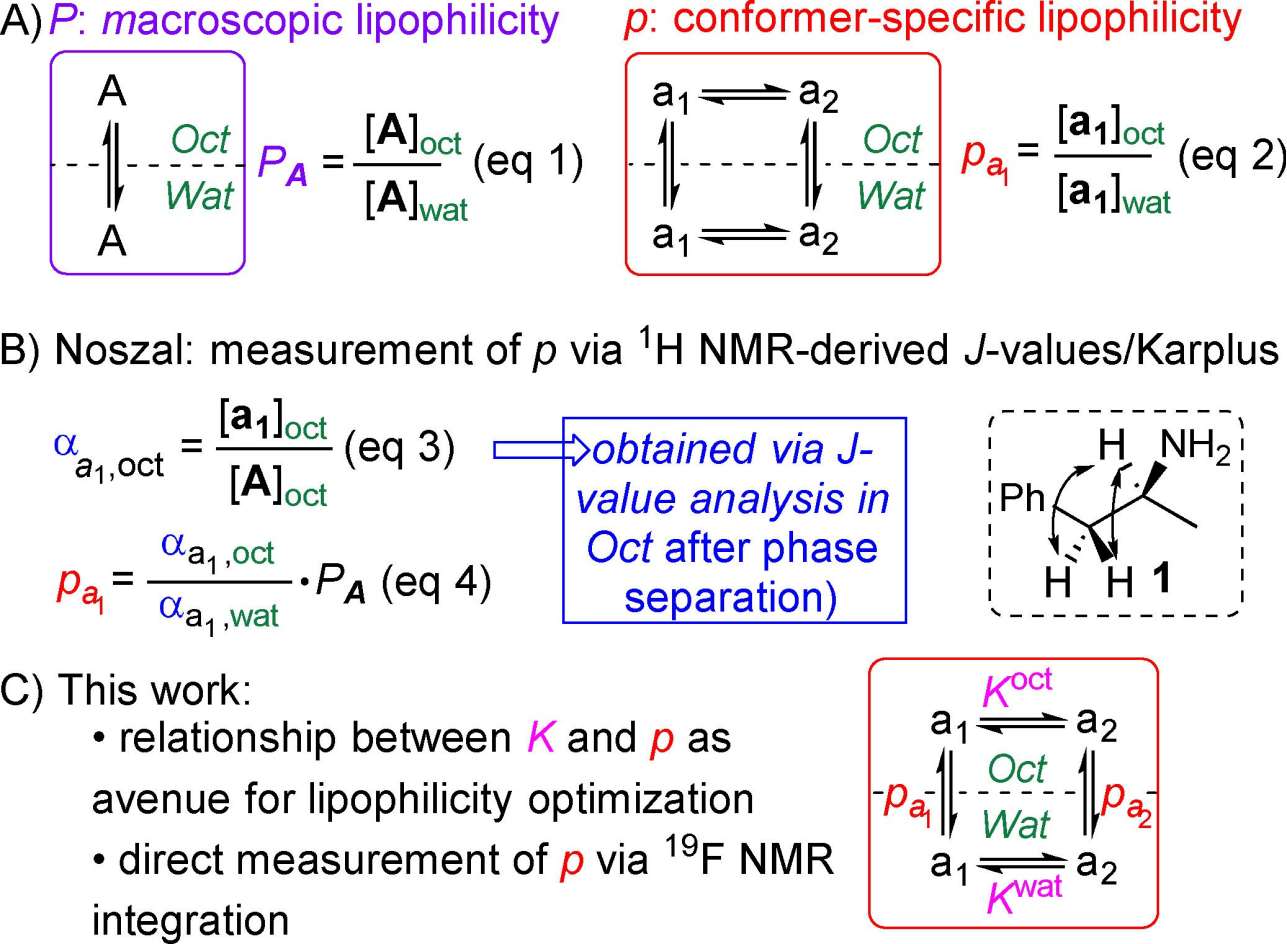
Macroscopic vs microscopic lipophilicity, measurement of the latter.

It wasn't until 2002 that the first, albeit indirect, conformer‐specific lipophilicity measurements were achieved with compounds bearing vicinal *J*‐couplings, by the group of Noszal (**1**, Figure 1B).^[11]^ Upon partitioning equilibration and phase separation, conformer mole fractions α (Eq. 3) in both phases were determined by NMR *J*‐value analysis in each phase based on a Karplus analysis, allowing determination of *p* (Eq. 4). In this way, large conformational log*p* differences were found. However, there are two main problems associated with this approach. Firstly, sufficient ^3^ *J*‐couplings need to be measurable for unambiguous dihedral angle estimation, and secondly, *J*‐value interpretation through the empirical Altona–Haasnoot equation is approximate.

More common is the consideration of molecular conformations for rationalisation of log*P* values (eg to estimate dipoles etc),^[12]^ Evaluation of calculated conformer‐specific lipophilicities has been exemplified by Testa, who substantiated the “chameleonic behaviour” of morphine glucuronide: hydrophilic conformers exist in water but more lipophilic, folded conformers are adopted in lipidic media.^[13]^ The need to understand conformer specific physicochemical properties has recently gained great momentum as a strategy to obtain both water‐solubility and membrane permeability of so‐called “beyond Rule of 5 (bRo5)” molecules,^[14]^ and also with regard to strategies exploiting intramolecular hydrogen bonding.^[15]^ Nevertheless, these rely on calculated conformer specific properties due to the lack of experimental approaches to measure log*p* values, and/or on lipophilicity measurements in different solvents (eg Δlog*P*_oct‐tol_).^[16]^

Accurately calculating lipophilicities is not straightforward however. Calculated log*P* values obtained from 2D structures are clearly unsuitable to use for microscopic lipophilicities. For 3D structures, quantum chemistry calculations can also predict log*P* values, as these provide solvation energy differences between water and octanol for each conformer determining the conformer equilibria between, as well as within, the phases. However, in practice this proves difficult for systems involving small energy differences,^[17]^ while continuum solvation models also need to be applied to take into account the effect of the surroundings. Unfortunately, these implicit models are not able to take into consideration the effect of specific solvent‐solute intermolecular interactions. The situation in the system under study is further complicated by the large water content in octanol, and the fact that octanol itself can engage in hydrogen bonding. Hence, the experimental determination of conformer‐specific lipophilicities would be a crucial way to benchmark compounds during drug discovery optimization programs.

We report here how conformer equilibrium constants *K* in octanol and water are intricately linked to microscopic lipophilicities (Figure 1C), which allows log*p* values to be obtained indirectly. The presence of conformational equilibria distinguishes log*p* from log*P* optimization, and on this basis we propose a specific strategy towards optimizing microscopic lipophilicities. This is exemplified with a direct and convenient measurement of conformer‐specific lipophilicities using our ^19^F NMR based log*P* determination procedure,^[18]^ which is possible when conformers have observable and different ^19^F chemical shift values. Recently, Zafrani et al. reported the log*P* determination of trifluoromethyl ketones and their hydrates using this NMR method, which is based on the same principle.^[19]^

The principle of the strategy for log*p* optimization, and of the direct log*p* measurement, is illustrated for amide rotamers (Figure 2) in slow exchange in solution, where each rotamer signal can be integrated directly. This constitutes a system with four coupled equilibria of conformers between (refers to *p*) and within (refers to *K*) the two phases (Figure 2A). Our lipophilicity measurement procedure^[18]^ involves acquiring a ^19^F NMR spectrum of each phase after phase separation (Figure 2B), and measuring integration relative to an added internal reference. Three peaks are observed: one corresponding to the reference, and two corresponding to each amide rotamer. A ρ‐value for a given rotamer in a given phase is defined as the integration ratio of the rotamer signal and that of the reference (Eq. 5). Through taking the ratio of the ρ‐values of a rotamer, Equation 6 can be obtained,^[18]^ showing that its log*p* equals the known log*P* of the reference plus the log of the ratio of its ρ‐values. The overall log*P* of the amide is then determined by using the integration sum of its rotamer signals using the same equation.^[18]^

**Figure 2:**
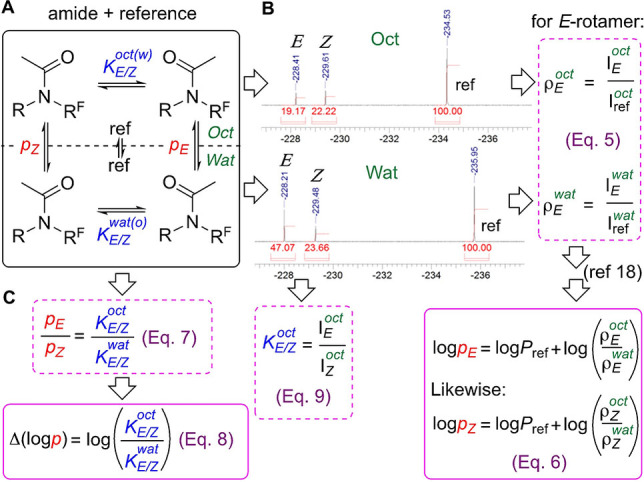
A) Equivalency between *K* and *p*‐values. B) Measurement of conformer‐specific lipophilicity. C) Relationship between *p* and *K*.

In the closed equilibrium system shown in Figure 2A, the ratio of the *p*‐values equates to the ratio of the *K*‐values ((Eq. 7), Figure 2C), so that the difference in log*p* values equals the log of the *K*‐value ratio (Eq. 8). In other words: the lipophilicity difference between two conformers is directly related to the change between their equilibrium concentration ratios in water and octanol. The *K*‐values can be obtained through integration measurement (Eq. 8). Hence, Equation 8 provides an avenue for rational log*p* optimization through influencing of *K*‐values in octanol and water.

The ρ and *K* determinations require assignment of each fluorine signal in each phase to a particular rotamer, as it was observed that the relative position of *cis*‐and *trans*‐amide rotamer resonances in the ^19^F NMR spectrum in octanol was not always the same as that in water. Conformer signal assignment in the water phase is easily accomplished by a control experiment using (octanol‐saturated) D_2_O by standard NOESY analysis combined with peak integration (see Supporting Information). As perdeuterated octanol is very expensive, this was experimentally non‐trivial for cases when the huge octanol solvent R‐CH_2_OH signal and its ^13^C satellites obscure proton resonances that are required for rotamer assignment. Solvent suppression techniques are impractical, and skew the integration ratio. We therefore introduced 1,1‐dideuteriooctanol (C_7_H_15_CD_2_OH), which considerably facilitated rotamer assignment. It is prepared on large scale by a modified Bouveault–Blanc reduction^[20]^ of cheap methyl octanoate with EtOD as deuterium source (see Supporting Information).

With this methodology, a panel of fluorinated *N*‐acyl piperidines and pyrrolidines as generic examples of abundant drug scaffolds^[21]^ were investigated (**2**–**5**, Figure 3A), next to a panel of 4‐fluorinated prolines (**6**/**7**, Figure 3B), as specific examples where *cis/trans* isomerism is of particular interest.[^22^, ^23^] Proline is the only amino acid for which measurable amounts of *cis*‐amide (proline nomenclature) rotamers are observed, a behaviour which has profound biological implications.^[24]^ Wennemers et al. had shown that the rotamer ratio of **6 a**/**6 b** is solvent‐dependent,^[25]^ prompting us to investigate their log*p* values. As expected, the macroscopic log*P* of the difluorinated derivatives is larger than that of their monofluorinated counterparts for both the pyrrolidines (cf **5**/**4** and **3**/**2**) and the prolines (**7 a**/**6 a** and **7 b**/**6 b**). Additionally, for the prolines, amides **b** have a lower log*P* compared to esters **a**.^[26]^

**Figure 3:**
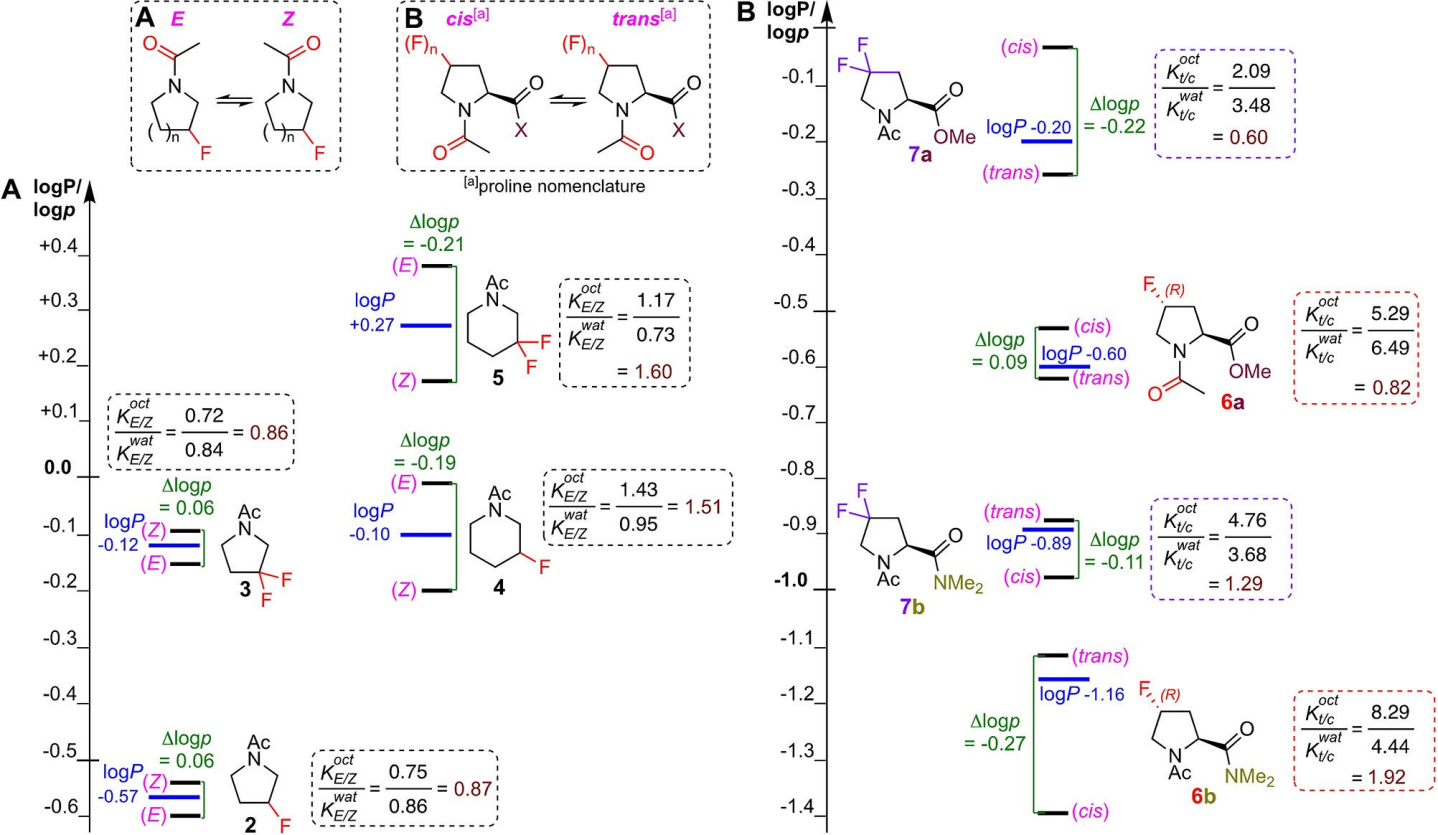
Lipophilicities (log*P*) and conformer‐specific lipophilicities (log*p*) of fluorinated pyrrolidines and piperidines (A), and prolines (B).

The log*p* values of the **2** and **3** rotamers are similar. A *gauche‐effect* effectively results in **2 ax** as the only ring conformer (Figure 4i). While there is little difference in rotamer dipole moment, the least polar *Z*‐rotamer turns out to be the most lipophilic. However, for **3**, the *Z* rotamer is the most polar (not shown), while still being the most lipophilic, as the solvation energies still result in similar *K*‐values as for **2**. The *gauche* effect is less defining for the ring conformation of **4**, with a significant amount of **4 eq** in the octanol phase (Figure 4ii). Given the low dipole moment of **4 eq**, its significant stabilisation in octanol results in a marked increase in $K_{E/Z}^{oct}$ value, making the *E*‐rotamer now the most lipophilic.

**Figure 4:**
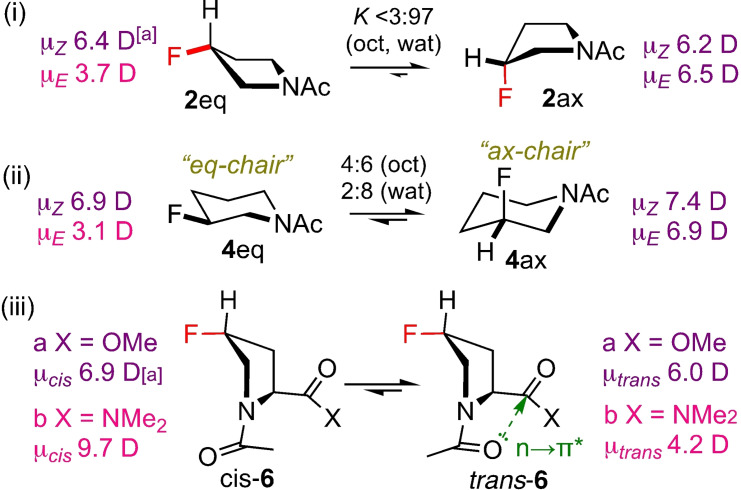
Conformations and dipole moments of selected examples. ^[a]^ Calculated in octanol at the SMD/MN15/aug‐cc‐pVTZ//MN15/cc‐pVTZ level of theory.

For the prolines, the rotamers of 4*R*‐4‐fluoroproline methyl ester **6 a** have a small log*p* difference, with the *cis*‐rotamer the most lipophilic. Introducing a second fluorine (**7 a**) leads to a reduction in both *K*_t/c_‐values, which is more pronounced for $K_{t/c}^{oct}$ , hence leading to an enhanced log*p* difference. However, changing the ester in **6 a** to the amide in **6 b** enhances the $K_{t/c}^{oct}$ value, but leads to a reduction in the $K_{t/c}^{wat}$ , resulting in $K_{t/c}^{oct}>K_{t/c}^{wat}$ , hence the *trans*‐rotamer is now the most lipophilic. An amide group displays increased n→π* stabilization (Figure 4iii) compared to an ester, serving to increase *K*_t/c_. However, the much larger difference in dipole moment for the **6 b** rotamers, with μ_*cis*_(**6 b**)>μ_*cis*_(**6 a**) (Figure 4iii) and vice versa for μ_*trans*_, results in extra stabilization of the polar *cis*‐**6 b** in water, overriding the effect of the n→π* hyperconjugation, leading to a decreased $K_{t/c}^{wat}$ value compared to that of **6 a**. Taken together, the simple modifications shown in Figure 3 clearly affect conformer equilibria, and thus conformer lipophilicities.

The usefulness of conformation‐specific lipophilicities in a drug discovery context results from their more detailed description: instead of a one‐point value (log*P*), a series of conformation‐dependent values are obtained, the ensemble of which has been defined by Testa as “property space”.^[27]^ The physiological relevance is clear: a log*P* value is a population‐weighted macroscopic value, while log*p* values can be considered being *effective* lipophilicities of the actual species existing in solution. This is especially of interest for so‐called “sensitive molecules”^[27]^ when conformations have very different log*p* values. The greater this difference or “amplitude”, the greater the propensity for the molecule to adapt to its environment.^[28]^ Crucially, this may blur definitions of optimal lipophilicity ranges: while a log*P* value could fall outside an optimal (application‐specific) lipophilicity range, one conformer, could fall *within* this range. For example, a molecule with a “too low” log*P* may have a conformer with higher log*p* that may facilitate membrane transport, with molecular chameleons as extreme examples.

Equation 8 (Figure 2) indicates that it is not necessary to conduct lipophilicity determinations to have information about conformer lipophilicities: knowledge of the conformer populations and *K*‐values in each solvent will give the difference in the log*p* values (though not their absolute values). In this case, octanol‐saturated water and water‐saturated octanol would need to be used as solvents.^[29]^ Furthermore, by introducing Eqs 10 and 11, which express the relationship between log*p*, log*P* and the *K* values (Figure 5; see Supporting Information), absolute microscopic lipophilicity values can be obtained by measuring the *K*‐values and the macroscopic lipophilicity *P*. Of course, these can also be obtained using different methods (e.g. that do not rely on the presence of fluorine, or accommodate fast exchange),^[30]^ making the approach generally applicable.

**Figure 5:**
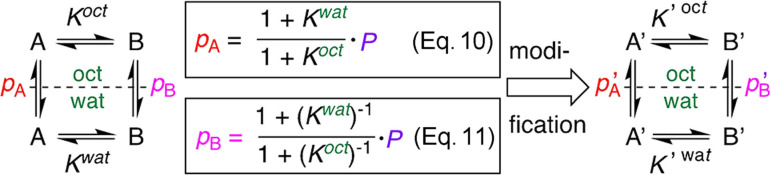
Relationship between log*p* and log*P*: the dependence on the *K* values. A and B are species in equilibrium. A′ and B′ arise after a structural modification.

Hence, optimization of conformer lipophilicities can be achieved by focusing on conformational equilibria: if a structural change is introduced that leads to a comparatively larger stabilization of conformer B′ in the octanol phase compared to the water phase [Δ(*K*′^(oct)^−*K* ^oct^)>Δ(*K*′^(wat)^−*K* ^wat^)], then *p*′_A_<*p*_A_ (over and above, but in the first instance independent from, any inherent change in macroscopic log*P* caused by the structural modification), and vice versa for *p*′_B_ and *p*_B_. A key illustration of this is the “scaffold‐hopping” from a difluorinated pyrrolidine ring to a monofluorinated piperidine such as **3**→**4** (Figure 3): while there is no impact on log*P*, the difference in *K*‐values results in a significant impact on log*p*, clearly illustrating the additional information log*p* measurements provide. In addition, given protein binding pockets are generally more hydrophobic than the surrounding aqueous environment, optimising conformer lipophilicity values provide another useful avenue to maximising bioactive conformations. This can be illustrated with proline derivatives **7 a** and **7 b**, which have similar *trans*/*cis* ratios in water. However, in octanol, for the ester **7 a**, this ratio decreases while for **7 b**, it increases. This translates to a larger log*p* for the *cis*‐rotamer for **7 a**, and for the *trans*‐rotamer in **7 b**. Hence, depending on which is deemed to be the bioactive conformation, this could, in this example, guide the nature of carboxylate functionalisation before additional optimisation of the macroscopic log*P*.

Rational optimization requires insight in the difference in the change in *K‐*value in water vs. octanol upon structural modification, and also how these may depend on the macroscopic log*P* values. Experimental log*p* determination in drug discovery programs will be invaluable in this regard. For the compounds reported herein, computational analysis was only moderately effective in predicting *K* ^oct^/*K* ^wat^ ratios (see Supporting Information).

The relationship between conformer equilibrium constant *K* and conformer lipophilicity log*p* enables a new direction towards rational lipophilicity optimization, by focusing on the difference of *K* values in water and in octanol upon structural modification. In addition, knowledge of log*p* values can guide structural modifications to optimize the lipophilicity of the bioactive conformation. The *K*‐*p* relationship also shows how conformer‐specific lipophilicity values can be independently obtained from the macroscopic lipophilicity and the phase‐specific conformer equilibrium constants, while for specific cases involving slow chemical exchange of the species in solution, direct, straightforward measurement of conformer specific lipophilicities by our ^19^F NMR based method is possible. This is the first time that direct log*p* measurement has been demonstrated. For compounds that are in fast exchange on the NMR time‐scale, conformer populations and thus conformer specific lipophilicities can be indirectly derived from NMR conformational analysis, making the approach generally applicable. In this regard, while there are many types of substrates where *cis*‐*trans* isomerisms are of fundamental biological interest,^[22]^ our analysis is not restricted to conformers alone, and one can envision a more general “equilibrating species” scenario involving tautomer/ anomer equilibria, and even species in chemical equilibrium, for example hydrates,^[19]^ or any catalysed reaction, which may inspire novel thinking in property control in drug discovery. We anticipate that quantitative conformation‐dependent physicochemical property measurements will inspire conformational control strategies to stabilise bioactive conformations that also shield polarity, or that are in conformational exchange equilibrium with conformations capable of shielding polarity leading to water‐soluble, membrane permeable entities,^[31]^ potentially paving the way to developing 3D conformer‐specific lipophilicity efficiencies. In addition, availability of experimental conformer dependent physico‐chemical properties will not only help rationalise disconnections between calculated properties from 2D structures, but it will enable generation and validation of 3D based physico‐chemical property models that may be impactful in the rational design of bRo5 compounds.

## Conflict of interest

The authors declare no conflict of interest.

## Supporting information

As a service to our authors and readers, this journal provides supporting information supplied by the authors. Such materials are peer reviewed and may be re‐organized for online delivery, but are not copy‐edited or typeset. Technical support issues arising from supporting information (other than missing files) should be addressed to the authors.

Supporting Information

## Data Availability

The data that support the findings of this study are available in the supplementary material of this article.
