# Supplementary material for: Relating Conformational Equilibria to Conformer‐Specific Lipophilicities: New Opportunities in Drug Discovery
Source: Angew Chem Int Ed Engl. 2021 Dec 29;61(7):e202114862. doi: 10.1002/anie.202114862 (PMC9304282; doi:10.1002/anie.202114862)
Supplement: Supplementary file 1 — Supporting Information [file ANIE-61-0-s001.pdf]

Angewandte Chemie
Eine Zeitschrift der Gesellschaft Deutscher Chemiker

Supporting Information

Relating Conformational Equilibria to Conformer-Specific Lipophilicities: New Opportunities in Drug Discovery

B. Linclau, Z. Wang, B. Jeffries, J. Graton, R. J. Carbajo, D. Sinnaeve, J.-Y. Le Questel, J. S. Scott, E. Chiarparin*

Table of Contents

1 Detailed graph with amide log*P/p* values (Figure S1) 6

2 Derivation of equations 10 and 11 7

3 The log*P* determination by ^{19}F NMR 8

4 lipophilicity determination 9

4.1 Confirmation of slow exchange in water and octanol 9

4.2 Amide rotamer assignment in water and octanol phase 9

4.2.1 Rotamer assignment in water (D_2O) 9

4.2.2 Rotamer assignments in octanol 11

4.2.2.1 Requirement to also independently determine the assignment of the rotamers in octanol 11

4.2.2.2 The use of (1,1-D_2)-octan-1-ol: assignment of *N*-acetyl-3-fluoropyrrolidine **2** in octanol 11

4.2.2.3 Rotamer assignment of *N*-acetyl-3,3-difluoropyrrolidine **3** in octanol 13

4.2.2.4 Rotamer assignment of *N*-acetyl-3-fluoropiperidine **4** in octanol 14

4.2.2.5 Rotamer assignment of *N*-acetyl-3,3-difluoropiperidine **5** in octanol 15

4.2.2.6 Rotamer assignment of *N*-acetyl-(2*S*,4*R*)-4-fluoroproline dimethyl amide **6b** in octanol 17

4.2.2.7 Rotamer assignment of *N*-acetyl-(2*S*)-4,4-difluoroproline dimethyl amide **7b** 18

4.3 Detailed experimental data for the logP determinations (ρ_{oct} and ρ_{wat} values for each experiment) 21

4.3.1 Standard NMR settings 21

4.3.2 N-Acetyl-3-fluoropyrrolidine **2** 21

4.3.3 *N*-Acetyl-3,3-difluoropyrrolidine **3** 22

4.3.4 *N*-Acetyl-3-fluoropiperdine **4** 22

4.3.5 *N*-Acetyl-3,3-difluoropiperdine **5** 23

4.3.6 N-acetyl-4*R*-FPro-OMe **6a** 23

4.3.7 N-acetyl-4*R*-FPro-NMe_2 **6b** 24

4.3.8 N-Acetyl-4,4-F_2Pro-OMe **7a** 25

4.3.9 N-Acetyl-4,4-F_2Pro-NMe_2 **7b** 25

5 Conformational analysis 26

5.1 General 26

5.2 *Pyrrolidines and piperidines **2–5** 26*

5.2.1 N-Acetyl-3-fluoropyrrolidine **2** 26

5.2.2 *N*-Acetyl-3,3-difluoropyrrolidine **3** 27

5.2.3 *N*-Acetyl-3-fluoropiperidine **4** 27

5.2.4 *N*-Acetyl-3,3-difluoropiperidine **5** 29

5.3 Conformational analysis of the proline derivatives 30

5.3.1 N-Acetyl-(4*R*)-FPro-OMe **6a** 30

5.3.2 *N*-Acetyl-(4*R*)-FPro-NMe$_2$ **6b** 31
5.3.3 *N*-Acetyl-4,4-F$_2$Pro-OMe **7a** 32
5.3.4 *N*-Acetyl-4,4-F$_2$Pro-NMe$_2$ **7b** 33

6 Comparison experimental and calculated ($Kt/coct$) / ($Kt/cwat$) values 34

7 Structural coordinates and total energies 35

*7.1 N-Acetyl-3-fluoropyrrolidine **2**, ax-E-rotamer, solvent = n-octanol 35*

*7.2 N-Acetyl-3-fluoropyrrolidine **2**, eq-E-rotamer, solvent = n-octanol 35*

*7.3 N-Acetyl-3-fluoropyrrolidine **2**, ax-Z-rotamer, solvent = n-octanol 36*

*7.4 N-Acetyl-3-fluoropyrrolidine **2**, eq-Z-rotamer, solvent = n-octanol 36*

*7.5 N-Acetyl-3-fluoropyrrolidine **2**, ax-E-rotamer, solvent = water 37*

*7.6 N-Acetyl-3-fluoropyrrolidine **2**, eq-E-rotamer, solvent = water 37*

*7.7 N-Acetyl-3-fluoropyrrolidine **2**, ax-Z-rotamer, solvent = water 38*

*7.8 N-Acetyl-3-fluoropyrrolidine **2**, eq-Z-rotamer, solvent = water 38*

*7.9 N-Acetyl-3,3-difluoropyrrolidine **3**, E-rotamer, solvent = n-octanol 39*

*7.10 N-Acetyl-3,3-difluoropyrrolidine **3**, Z-rotamer, solvent = n-octanol 39*

*7.11 N-Acetyl-3,3-difluoropyrrolidine **3**, E-rotamer, solvent = water 40*

*7.12 N-Acetyl-3,3-difluoropyrrolidine **3**, Z-rotamer, solvent = water 40*

*7.13 N-Acetyl-3-fluoropiperidine **4**, ax_E-rotamer, solvent = n-octanol 41*

*7.14 N-Acetyl-3-fluoropiperidine **4**, ax_Z-rotamer, solvent = n-octanol 41*

*7.15 N-Acetyl-3-fluoropiperidine **4**, eq_E-rotamer, solvent = n-octanol 42*

*7.16 N-Acetyl-3-fluoropiperidine **4**, eq_Z-rotamer, solvent = n-octanol 42*

*7.17 N-Acetyl-3-fluoropiperidine **4**, ax_E-rotamer, solvent = water 43*

*7.18 N-Acetyl-3-fluoropiperidine **4**, ax_Z-rotamer, solvent = water 43*

*7.19 N-Acetyl-3-fluoropiperidine **4**, eq_E-rotamer, solvent = water 44*

*7.20 N-Acetyl-3-fluoropiperidine **4**, eq_Z-rotamer, solvent = water 44*

*7.21 N-Acetyl-3,3-difluoropiperidine **5**, E-rotamer, solvent = n-octanol 45*

*7.22 N-Acetyl-3,3-difluoropiperidine **5**, Z-rotamer, solvent = n-octanol 45*

*7.23 N-Acetyl-3,3-difluoropiperidine **5**, E-rotamer, solvent = water 46*

*7.24 N-Acetyl-3,3-difluoropiperidine **5**, Z-rotamer, solvent = water 46*

*7.25 N-Acetyl-(4R)-FPro-OMe **6a**, trans-rotamer, exo-pucker, psi1-rotamer, solvent=octanol 47*

*7.26 N-Acetyl-(4R)-FPro-OMe **6a**, trans-rotamer, exo-pucker, psi2-rotamer, solvent=octanol 47*

*7.27 N-Acetyl-(4R)-FPro-OMe **6a**, trans-rotamer, endo-pucker, psi1-rotamer, solvent=octanol 48*

*7.28 N-Acetyl-(4R)-FPro-OMe **6a**, trans-rotamer, endo-pucker, psi2-rotamer, solvent=octanol 48*

*7.29 N-Acetyl-(4R)-FPro-OMe **6a**, cis-rotamer, exo-pucker, psi1-rotamer, solvent=octanol 49*

*7.30 N-Acetyl-(4R)-FPro-OMe **6a**, cis-rotamer, exo-pucker, psi2-rotamer, solvent=octanol 50*

7.31 *N-Acetyl-(4R)-FPro-OMe* ***6a****, cis-rotamer, endo-pucker, psi1-rotamer, solvent=octanol* ... 50

7.32 *N-Acetyl-(4R)-FPro-OMe* ***6a****, cis-rotamer, endo-pucker, psi2-rotamer, solvent=octanol* ... 51

7.33 *N-acetyl-(4R)-FPro-OMe* ***6a****, trans-rotamer, exo-pucker, psi1-rotamer, solvent = water* ... 51

7.34 *N-Acetyl-(4R)-FPro-OMe* ***6a****, trans-rotamer, exo-pucker, psi2-rotamer, solvent = water* ... 52

7.35 *N-Acetyl-(4R)-FPro-OMe* ***6a****, trans-rotamer, endo-pucker, psi1-rotamer, solvent = water* ... 53

7.36 *N-Acetyl-(4R)-FPro-OMe* ***6a****, trans-rotamer, endo-pucker, psi2-rotamer, solvent = water* ... 53

7.37 *N-acetyl-(4R)-FPro-OMe* ***6a****, cis-rotamer, exo-pucker, psi1-rotamer, solvent = water* ... 54

7.38 *N-Acetyl-(4R)-FPro-OMe* ***6a****, cis-rotamer, exo-pucker, psi2-rotamer, solvent = water* ... 54

7.39 *N-acetyl-(4R)-FPro-OMe* ***6a****, cis-rotamer, endo-pucker, psi1-rotamer, solvent = water* ... 55

7.40 *N-Acetyl-(4R)-FPro-OMe* ***6a****, cis-rotamer, endo-pucker, psi2-rotamer, solvent = water* ... 56

7.41 *N-Acetyl-(4R)-FPro-NMe$_2$* ***6b****, trans-rotamer, exo-pucker, psi1-rotamer, solvent=octanol* ... 56

7.42 *N-Acetyl-(4R)-FPro-NMe$_2$* ***6b****, trans-rotamer, exo-pucker, psi2-rotamer, solvent=octanol* ... 57

7.43 *N-Acetyl-(4R)-FPro-NMe$_2$* ***6b****, trans-rotamer, endo-pucker, psi1-rotamer, solvent=octanol* ... 58

7.44 *N-Acetyl-(4R)-FPro-NMe$_2$* ***6b****, trans-rotamer, endo-pucker, psi2-rotamer, solvent=octanol* ... 58

7.45 *N-Acetyl-(4R)-FPro-NMe$_2$* ***6b****, cis-rotamer, exo-pucker, psi1-rotamer, solvent=octanol* ... 59

7.46 *N-Acetyl-(4R)-FPro-NMe$_2$* ***6b****, cis-rotamer, exo-pucker, psi2-rotamer, solvent=octanol* ... 60

7.47 *N-Acetyl-(4R)-FPro-NMe$_2$* ***6b****, cis-rotamer, endo-pucker, psi1-rotamer, solvent=octanol* ... 60

7.48 *N-Acetyl-(4R)-FPro-NMe$_2$* ***6b****, cis-rotamer, endo-pucker, psi2-rotamer, solvent=octanol* ... 61

7.49 *N-Acetyl-(4R)-FPro-NMe$_2$* ***6b****, trans-rotamer, exo-pucker, psi1-rotamer, solvent = water* ... 62

7.50 *N-Acetyl-(4R)-FPro-NMe$_2$* ***6b****, trans-rotamer, endo-pucker, psi1 rotamer, solvent = water* ... 62

7.51 *N-Acetyl-(4R)-FPro-NMe$_2$* ***6b****, trans-rotamer, exo-pucker, psi2-rotamer, solvent = water* ... 63

7.52 *N-Acetyl-(4R)-FPro-NMe$_2$* ***6b****, trans-rotamer, endo-pucker, psi2-rotamer, solvent = water* ... 64

7.53 *N-Acetyl-(4R)-FPro-NMe$_2$* ***6b****, cis-rotamer, endo-pucker, psi1-rotamer, solvent = water* ... 64

7.54 *N-Acetyl-(4R)-FPro-NMe$_2$* ***6b****, cis-rotamer, exo-pucker, psi1-rotamer, solvent = water* ... 65

7.55 *N-Acetyl-(4R)-FPro-NMe$_2$* ***6b****, cis-rotamer, exo-pucker, psi2-rotamer, solvent = water* ... 66

7.56 *N-Acetyl-(4R)-FPro-NMe$_2$* ***6b****, cis-rotamer, endo-pucker, psi2-rotamer, solvent = water* ... 66

7.57 *N-Acetyl-(4,4)-F$_2$Pro-OMe* ***7a****, trans-rotamer, exo-pucker, psi1-rotamer, solvent=octanol* ... 67

7.58 *N-Acetyl-(4,4)-F$_2$Pro-OMe* ***7a****, trans-rotamer, exo-pucker, psi2-rotamer, solvent=octanol* ... 68

7.59 *N-Acetyl-(4,4)-F$_2$Pro-OMe* ***7a****, trans-rotamer, endo-pucker, psi1-rotamer, solvent=octanol* ... 68

7.60 *N-Acetyl-(4,4)-F$_2$Pro-OMe* ***7a****, trans-rotamer, endo-pucker, psi2-rotamer, solvent=octanol* ... 69

7.61 *N-Acetyl-(4,4)-F$_2$Pro-OMe* ***7a****, cis-rotamer, exo-pucker, psi1-rotamer, solvent=octanol* ... 70

7.62 *N-Acetyl-(4,4)-F$_2$Pro-OMe* ***7a****, cis-rotamer, exo-pucker, psi2-rotamer, solvent=octanol* ... 70

7.63 *N-Acetyl-(4,4)-F$_2$Pro-OMe* ***7a****, cis-rotamer, endo-pucker, psi1-rotamer, solvent=octanol* ... 71

7.64 *N-Acetyl-(4,4)-F$_2$Pro-OMe* ***7a****, cis-rotamer, endo-pucker, psi2-rotamer, solvent=octanol* ... 72

7.65 *N-acetyl-(4,4)-F$_2$Pro-OMe* ***7a****, trans-rotamer, exo-pucker, psi1-rotamer, solvent = water* ... 72

7.66 *N-Acetyl-(4,4)-F_2Pro-OMe **7a**, trans-rotamer, exo-pucker, psi2-rotamer, solvent = water* ... 73
7.67 *N-Acetyl-(4,4)-F_2Pro-OMe **7a**, trans-rotamer, endo-pucker, psi1-rotamer, solvent = water* ... 73
7.68 *N-Acetyl-(4,4)-F_2Pro-OMe **7a**, trans-rotamer, endo-pucker, psi2-rotamer, solvent = water* ... 74
7.69 *N-Acetyl-(4,4)-F_2Pro-OMe **7a**, cis-rotamer, exo-pucker, psi1-rotamer, solvent = water* ... 75
7.70 *N-Acetyl-(4,4)-F_2Pro-OMe **7a**, cis-rotamer, exo-pucker, psi2-rotamer, solvent = water* ... 75
7.71 *N-acetyl-(4,4)-F_2Pro-OMe **7a**, cis-rotamer, endo-pucker, psi1-rotamer, solvent = water* ... 76
7.72 *N-Acetyl-(4,4)-F_2Pro-OMe **7a**, cis-rotamer, endo-pucker, psi2-rotamer, solvent = water* ... 76
7.73 *N-Acetyl-(4,4)-F_2Pro-NMe_2 **7b**, trans-rotamer, exo-pucker, psi1-rotamer, solvent=octanol* ... 78
7.74 *N-Acetyl-(4,4)-F_2Pro-NMe_2 **7b**, trans-rotamer, exo-pucker, psi2-rotamer, solvent=octanol* ... 78
7.75 *N-Acetyl-(4,4)-F_2Pro-NMe_2 **7b**, trans-rotamer, endo-pucker, psi1-rotamer, solvent=octanol* ... 79
7.76 *N-Acetyl-(4,4)-F_2Pro-NMe_2 **7b**, trans-rotamer, endo-pucker, psi2-rotamer, solvent=octanol* ... 80
7.77 *N-Acetyl-(4,4)-F_2Pro-NMe_2 **7b**, cis-rotamer, exo-pucker, psi1-rotamer, solvent=octanol* ... 80
7.78 *N-Acetyl-(4,4)-F_2Pro-NMe_2 **7b**, cis-rotamer, exo-pucker, psi2-rotamer, solvent=octanol* ... 81
7.79 *N-Acetyl-(4,4)-F_2Pro-NMe_2 **7b**, cis-rotamer, endo-pucker, psi1-rotamer, solvent=octanol* ... 82
7.80 *N-Acetyl-(4,4)-F_2Pro-NMe_2 **7b**, cis-rotamer, endo-pucker, psi2-rotamer, solvent=octanol* ... 82
7.81 *N-Acetyl-(4,4)-F_2Pro-NMe_2 **7b**, trans-rotamer, exo-pucker, psi1-rotamer, solvent = water* ... 83
7.82 *N-Acetyl-(4,4)-F_2Pro-NMe_2 **7b**, trans-rotamer, exo-pucker, psi2-rotamer, solvent = water* ... 84
7.83 *N-Acetyl-(4,4)-F_2Pro-NMe_2 **7b**, trans-rotamer, endo-pucker, psi1-rotamer, solvent = water* ... 84
7.84 *N-Acetyl-(4,4)-F_2Pro-NMe_2 **7b**, trans-rotamer, endo-pucker, psi2-rotamer, solvent = water* ... 85
7.85 *N-Acetyl-(4,4)-F_2Pro-NMe_2 **7b**, cis-rotamer, exo-pucker, psi1-rotamer, solvent = water* ... 86
7.86 *N-Acetyl-(4,4)-F_2Pro-NMe_2 **7b**, cis-rotamer, exo-pucker, psi2-rotamer, solvent = water* ... 86
7.87 *N-Acetyl-(4,4)-F_2Pro-NMe_2 **7b**, cis-rotamer, endo-pucker, psi1-rotamer, solvent = water* ... 87
7.88 *N-Acetyl-(4,4)-F_2Pro-NMe_2 **7b**, cis-rotamer, endo-pucker, psi2-rotamer, solvent = water* ... 88

8 Compound synthesis and characterisation ... 89

8.1 *α,α-Deuterated octanol* ... 89
8.2 *Known compounds* ... 89
8.3 *N-Acetyl-3-fluoropyrrolidine (**2**)* ... 89
8.4 *N-Acetyl-3,3-difluoropyrrolidine (**3**)* ... 90
8.5 *N-Acetyl-3-fluoropiperidine (**4**)* ... 90
8.6 *N-Acetyl-3,3-difluoropiperidine (**5**)* ... 91
8.7 *N-acetyl-(2S)-4,4-difluoroproline dimethyl amide (**10b**)* ... 91

9 NMR spectra of novel compounds ... 93

9.1 *N-Acetyl-3-fluoropyrrolidine (**2**): ^{1}H NMR spectrum ($CDCl_3$, 400 MHz)* ... 93
9.2 *N-Acetyl-3-fluoropyrrolidine (**2**): ^{13}C{^{1}H} NMR spectrum ($CDCl_3$, 101 MHz)* ... 93
9.3 *N-Acetyl-3-fluoropyrrolidine (**2**): ^{19}F NMR spectrum ($CDCl_3$, 376 MHz)* ... 94

9.4 *N-Acetyl-3-fluoropyrrolidine (**2**): ^{19}F {H} NMR spectrum (CDCl$_3$, 376 MHz)*......94

9.5 *N-Acetyl-3,3-difluoropyrrolidine (**3**): ^{1}H NMR spectrum (CDCl$_3$, 400 MHz)*......95

9.6 *N-Acetyl-3,3-difluoropyrrolidine (**3**): ^{13}C{^{1}H} NMR spectrum (CDCl$_3$, 101 MHz)*......95

9.7 *N-Acetyl-3,3-difluoropyrrolidine (**3**): ^{19}F NMR spectrum (CDCl$_3$, 376 MHz)*......96

9.8 *N-Acetyl-3,3-difluoropyrrolidine (**3**): ^{19}F ^{1}{H} NMR spectrum (CDCl$_3$, 376 MHz)*......96

9.9 *N-Acetyl-3-fluoropiperidine (**4**): ^{1}H NMR spectrum (CDCl$_3$, 400 MHz)*......97

9.10 *N-Acetyl-3-fluoropiperidine (**4**): ^{13}C{^{1}H} NMR spectrum (CDCl$_3$, 101 MHz)*......97

9.11 *N-Acetyl-3-fluoropiperidine (**4**): ^{19}F NMR spectrum (CDCl$_3$, 376 MHz)*......98

9.12 *N-acetyl-3-fluoropiperidine (**4**): ^{19}F ^{1}{H} NMR spectrum (CDCl$_3$, 376 MHz)*......98

9.13 *N-Acetyl-3,3-difluoropyrrolidine (**5**): ^{1}H NMR spectrum (CDCl$_3$, 400 MHz)*......99

9.14 *N-Acetyl-3,3-difluoropyrrolidine (**5**): ^{13}C{^{1}H} NMR spectrum (CDCl$_3$, 101 MHz)*......99

9.15 *N-Acetyl-3,3-difluoropyrrolidine (**5**):^{19}F NMR spectrum (CDCl$_3$, 376 MHz)*......100

9.16 *N-Acetyl-3,3-difluoropyrrolidine (**5**):^{19}F {H} NMR spectrum (CDCl$_3$, 376 MHz)*......100

9.17 *N-(Acetyl)-(2S)-4,4-difluoroproline dimethyl amide **7b**: ^{1}H NMR spectrum (CDCl$_3$, 400 MHz)*......101

9.18 *N-(Acetyl)-(2S)-4,4-difluoroproline dimethyl amide **7b**: ^{13}C{^{1}H} NMR spectrum (CDCl$_3$, 100 MHz)*......101

9.19 *N-(Acetyl)-(2S)-4,4-difluoroproline dimethyl amide **7b**: ^{19}F NMR spectrum (CDCl$_3$, 471 MHz)*......102

9.20 *N-(Acetyl)-(2S)-4,4-difluoroproline dimethyl amide **7b**: ^{19}F{^{1}H} NMR spectrum (CDCl$_3$, 471 MHz)*......102

References......103

10 103

1 Detailed graph with amide log*P*/*p* values (Figure S1)

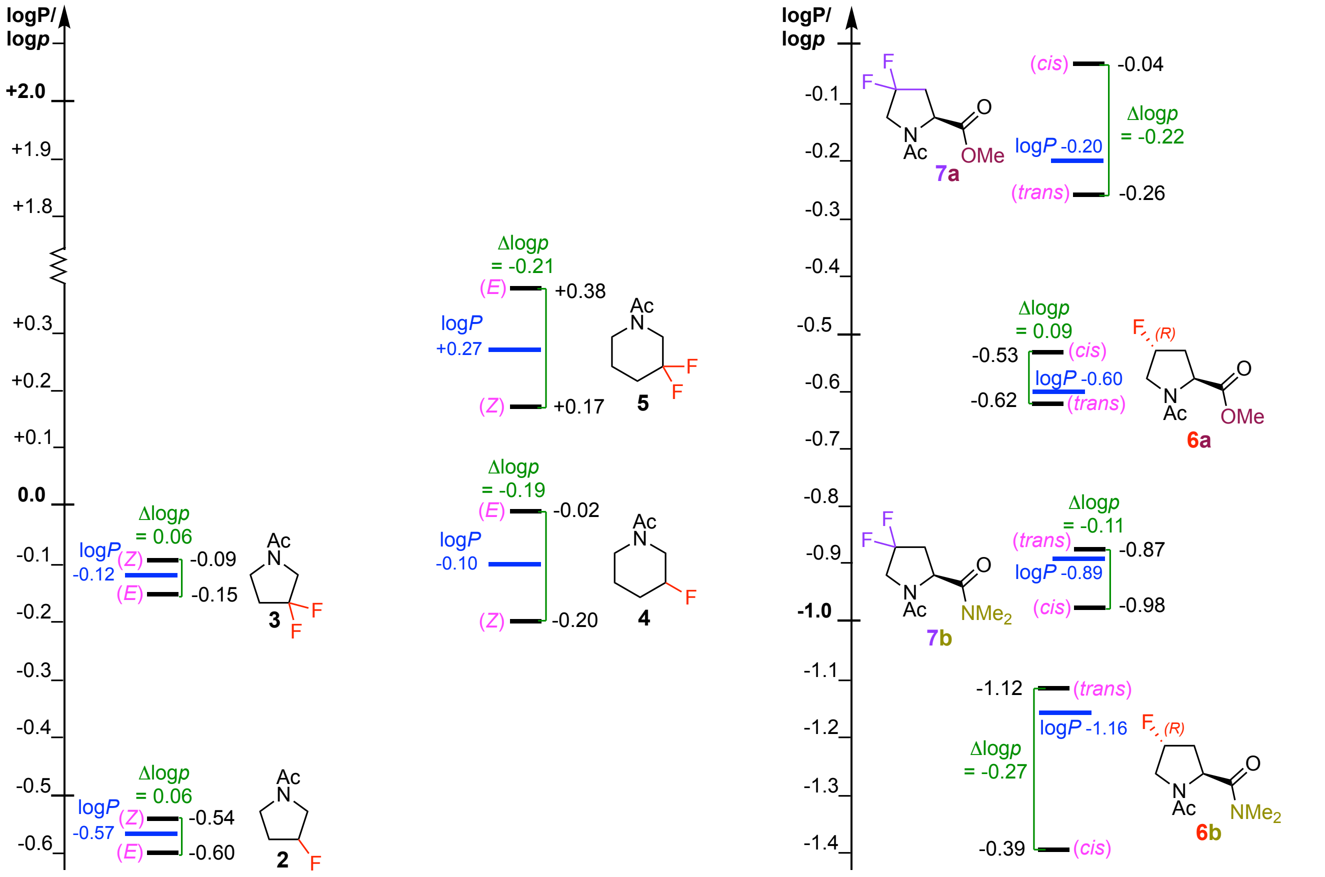

Figure S1.

2 Derivation of equations 10 and 11

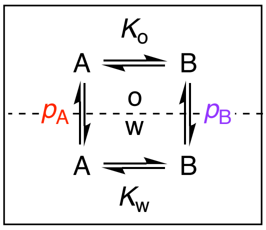

Standard definitions:

$$K_o = \frac{c_o^B}{c_o^A} \qquad K_w = \frac{c_w^B}{c_w^A} \qquad p_A = \frac{c_o^A}{c_w^A} \qquad p_B = \frac{c_o^B}{c_w^B} \qquad P = \frac{c_o^A + c_o^B}{c_w^A + c_w^B}$$

Dividing K_o by K_w:

$$\frac{K_o}{K_w} = \frac{c_o^B}{c_o^A} \cdot \frac{c_w^A}{c_w^B}$$

$$= \frac{p_B}{p_A}$$

$$p_B = p_A \cdot \frac{K_o}{K_w} \quad \text{(eq 12)}$$

$$p_A = p_B \cdot \frac{K_w}{K_o} \quad \text{(eq 13)}$$

In the equation of P, displace all terms except c_o^A:

$$P = \frac{c_o^A + c_o^B}{c_w^A + c_w^B}$$

$$c_o^B = K_o \cdot c_o^A$$

$$c_w^A = \frac{c_o^A}{p_A}$$

$$c_w^B = \frac{c_o^B}{p_B} = \frac{K_o \cdot c_o^A}{p_B}$$

$$P = \frac{c_o^A + K_o \cdot c_o^A}{\dfrac{c_o^A}{p_A} + \dfrac{K_o \cdot c_o^A}{p_B}}$$

Divide by c_o^A

$$= \frac{1 + K_o}{\dfrac{1}{p_A} + \dfrac{K_o}{p_B}}$$

Insert eq 12

$$= \frac{1 + K_o}{\dfrac{1}{p_A} + \dfrac{K_o}{p_A \cdot \dfrac{K_o}{K_w}}}$$

$$= \frac{1 + K_o}{\dfrac{1}{p_A} + \dfrac{K_w}{p_A}}$$

$$P = \frac{p_A (1 + K_o)}{1 + K_w}$$

$$p_A = P \cdot \frac{1 + K_w}{1 + K_o} \quad \text{(eq 10)}$$

Insert eq 13

$$= \frac{1 + K_o}{\dfrac{1}{p_B \cdot \dfrac{K_w}{K_o}} + \dfrac{K_o}{p_B}}$$

$$= \frac{1 + K_o}{\dfrac{K_o}{p_B \cdot K_w} + \dfrac{K_o}{p_B}}$$

$$P = p_B \frac{\dfrac{1}{K_o} + 1}{\dfrac{1}{K_w} + 1}$$

$$p_B = P \cdot \frac{1 + (K_w)^{-1}}{1 + (K_o)^{-1}} \quad \text{(eq 11)}$$

3 The log*P* determination by ^{19}F NMR

Lipophilicities were determined using a previously published protocol:[1] to a 10 mL pear-shaped flask was added the compound (1.0 - 10 mg) for log*P* determination, the reference compound (1.0 - 10 mg, with known log*P* value, e.g., 2,2,2-trifluoroethanol, log*P*: +0.36), water (2 mL) and *n*-octanol (2 mL). The resulting biphasic mixture was stirred (at 600 rpm) for 2 h at 25 °C, and then left without stirring for 16 h at 25 °C to allow phase separation. An aliquot of 0.5 mL was taken from each phase using 1 mL syringes with long needles and added to two separate NMR tubes. A deuterated NMR solvent (0.1 mL, e.g., acetone-d_6), or a capillary tube containing deuterated NMR solvent, was added to the NMR tubes to enable signal locking. Because of the volatility of the used compounds, the NMR tubes were sealed using a blowtorch. For NMR samples with directly added deuterated solvent, the tubes were inverted 20 times for mixing. For ^{19}F{^{1}H} NMR experiments, NMR parameters were set as follows: D1 30 sec for the octanol sample, D1 60 sec for the water sample; and O1P centered between two diagnostic fluorine peaks. If needed, an increased number of transients (NS) and/or narrower spectral window (SW) for a good S/N ratio (typically >300) was applied. After NMR data processing, integration ratios ρ_{oct} and ρ_{aq} (ρ_{oct} is defined as the integration ratio between the compound and the reference compound in the octanol sample; likewise for ρ_{aq}) were obtained, and used in the equation ($\log P^X = \log P^{ref} + \log(\rho_{oct}/\rho_{aq})$) to obtain the log*P* value of the compound. The log*P* measurement of each compound was run in triplicate. Log*P* values of non-fluorinated compounds were taken from the literature.

The standard deviation (σ) was calculated using the formula:

$$\sigma = \sqrt{\frac{1}{N}\sum_{i=1}^{N}(x_i - \bar{x})^2}$$

Where x_i are the individual estimated log*P* values and $\bar{x}$ is the mean average.

4 lipophilicity determination

4.1 Confirmation of slow exchange in water and octanol

In all cases, a variable temperature NMR experiment (in DMSO-d_6) displayed coalescence of the NMR resonances at elevated temperature. This allowed us to conclude that the different signals on the NMR spectra at room temperature originated from the presence of rotamers.

4.2 Amide rotamer assignment in water and octanol phase

In order to achieve integration of the fluorine signals for each rotamer, it must first be established which peak corresponds to which rotamer. This is achieved by a NOESY experiment in which the proximity of the methyl group and the ring protons can be established (by the observation of NOE cross peaks) as shown in **Figure S2** for compound **3**:

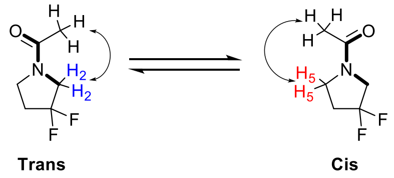

Figure S2. Assignment of the rotamers by nOe.

A typical procedure to achieve this involves:

- Assignment of the individual ring proton peaks in the ^{1}H NMR spectrum, especially the protons in the α-position of the amide nitrogen
- A NOESY experiment as shown above
- Establish which is the major and which is the minor isomer by integration
- Assign the fluorine peaks through their relative integration

4.2.1 Rotamer assignment in water (D_2O)

The assignment in water (D_2O) was straightforward, and illustrated here for **3**. For the amides under study, the ^{13}C{^{1}H} NMR interpretation was facile: C2 can be identified due to the $^2J_{C2\text{-}F}$ coupling constants (t, J = 32.16 Hz) and the chemical shift (adjacent to amide), while C5 can be recognised by its much smaller $^3J_{C5\text{-}F}$ coupling constants (t, J = 3.3 Hz). An analogous situation occurs for the piperidine compounds. Once the carbons had been assigned, the major and minor signals of H2 and H5 can be assigned via HSQC analysis

HSQC analysis (**Figure S3**) to assign the major and minor signals of H2 and H5.

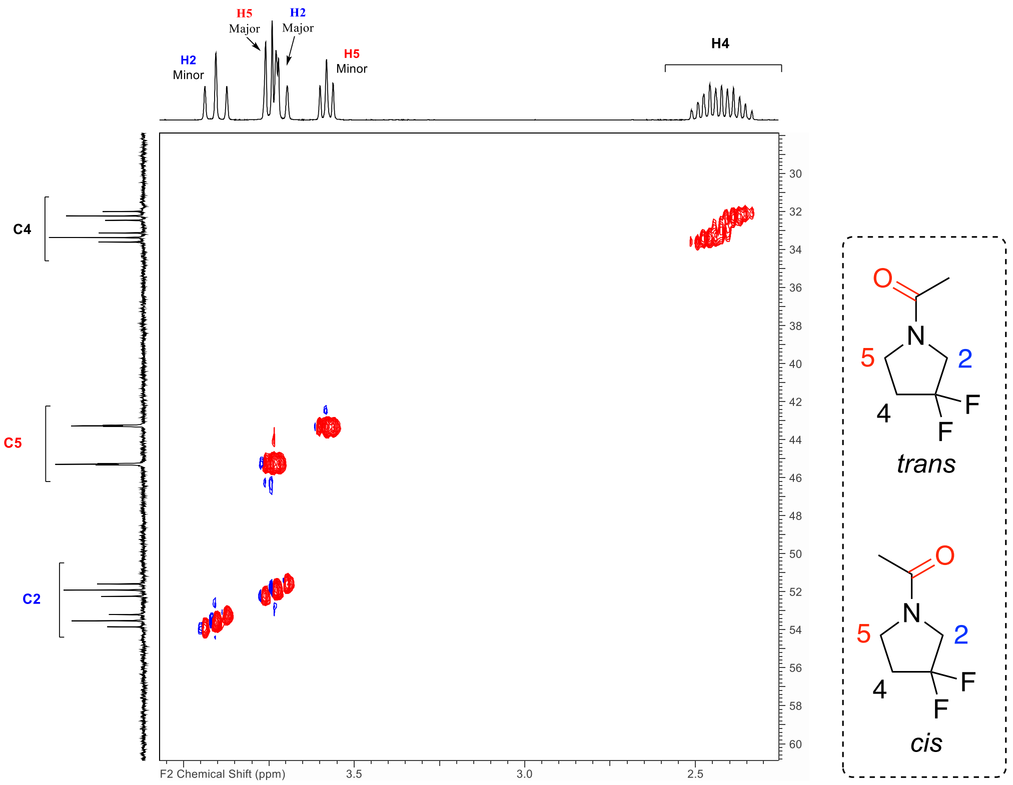

Figure S3. HSQC spectrum of **3** in D_2O.

In a NOESY spectrum a cross-peak will be observed for protons that are close in space. Cross-peaks of the acetate CH_3 with either H2 or H5 will allow to establish the identity of the rotamers (**Figure S4**): the minor acetate signal shows a cross-peak with a previously assigned H2 resonance, which confirms that the minor rotamer has the *trans*-conformation, while the signal for H5 of the minor isomer doesn't show a cross peak. The opposite is expected for the major integration signals and is observed, with the major H5 signal showing a cross-peak with the major acetate signal, and the major H2 signal showing no cross-peak. This confirms that the major rotamer of **3** in D_2O is the *cis*-conformation.

In this way the rotamer assignment in D_2O could be achieved for all amides investigated.

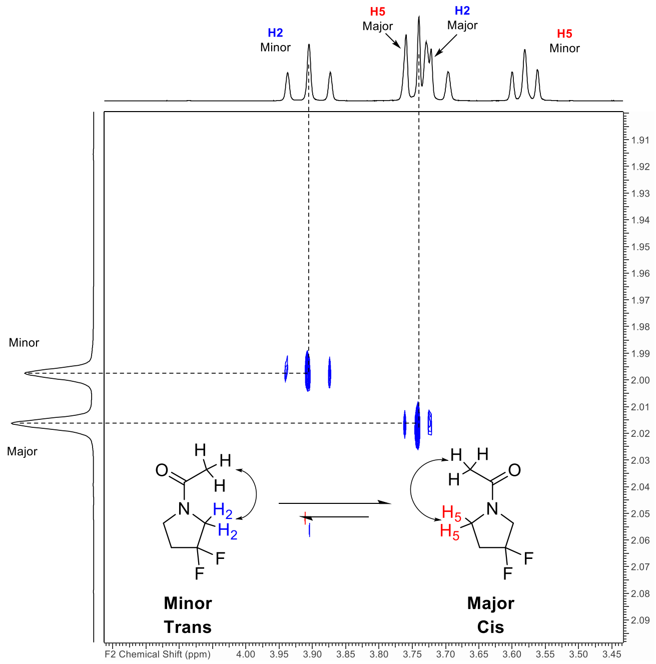

Figure S4. NOESY spectrum of compound **3** in D_2O.

4.2.2 Rotamer assignments in octanol

4.2.2.1 Requirement to also independently determine the assignment of the rotamers in octanol

With the rotamers accurately assigned in D_2O, their respective chemical shifts were compared to the ones observed in $CDCl_3$. It was established that the ^{19}F chemical shift values for the individual rotamers could deviate significantly between solvents. The chemical shifts in some cases even had effectively "reversed". This can be observed for **2** where the ^{19}F resonance for the *cis* conformer is more upfield in $CDCl_3$ compared to the resonance of the *trans*-rotamer (**Figure S5**), while in D_2O it is the other way round. Consequently, the assumption that the chemical shift values of the rotamer signals does not change between H_2O to octanol may result in incorrect log*P* values. Therefore, the rotamer assignment within octanol is crucial.

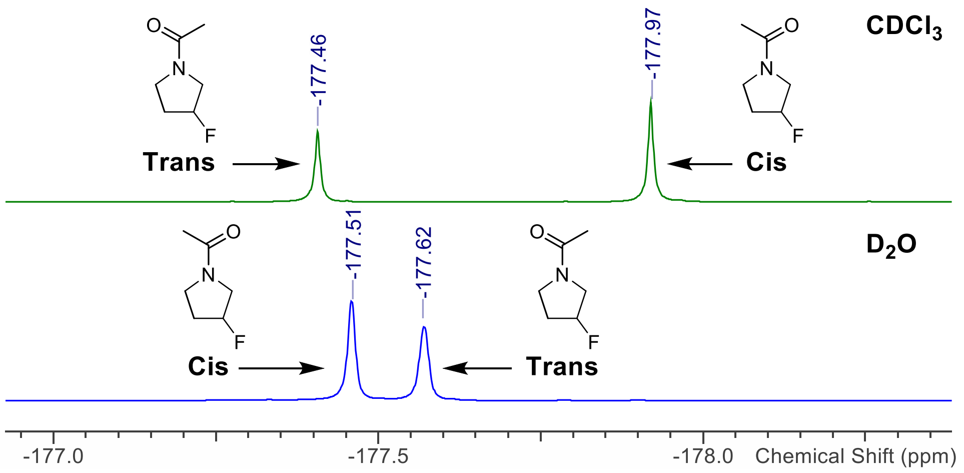

Figure S5. ^{19}F NMR spectrum detail of *N*-acetyl 3-fluoropyrollidine **2** in $CDCl_3$ and in D_2O, representing the change in chemical shifts in different solvents.

4.2.2.2 The use of (1,1-D_2)-octan-1-ol: assignment of *N*-acetyl-3-fluoropyrrolidine 2 in octanol

Given octanol does not contain a deuterium atom, a separate lock solvent is necessary. Given the rotamer ratio is solvent-dependent, a lock solvent (benzene-d6) was inserted in a sealed melting point capillary. Unfortunately, when dissolving ~5 mg of **2** in 0.5 mL of 1-octanol with a capillary insert of benzene-*d*6 (spectrum A, **Figure S6**), the H2 and H5 signals overlap with octanol's α,α-hydrogen signal and its ^{13}C-satellites, preventing rotamer assignment.

Hence, (1,1-D_2)-octan-1-ol was used: ~5 mg of **2** was dissolved in 0.5 mL of (1,1-*d*2)-octan-1-ol and minimal overlapping of these signals is observed (spectrum B). Following this success, a further ~5 mg of **2** was added to the NMR sample B and full characterisation ($^{13}C\{^1H\}$ NMR, HSQC and NOESY) was performed to enable the tentative assignment of the rotamers in (1,1-D_2)-octan-1-ol.

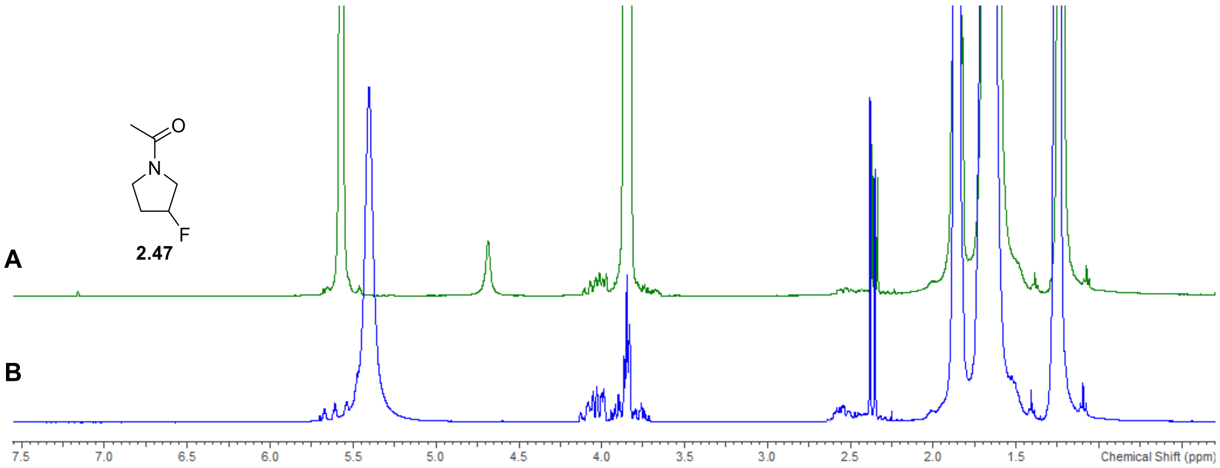

Figure S6. ^1H NMR spectrum depicting the comparison of N-acetyl-3-fluoropyrrolidine **2** in octanol (spectrum A) and (1,1-D_2)-octan-1-ol (spectrum B).

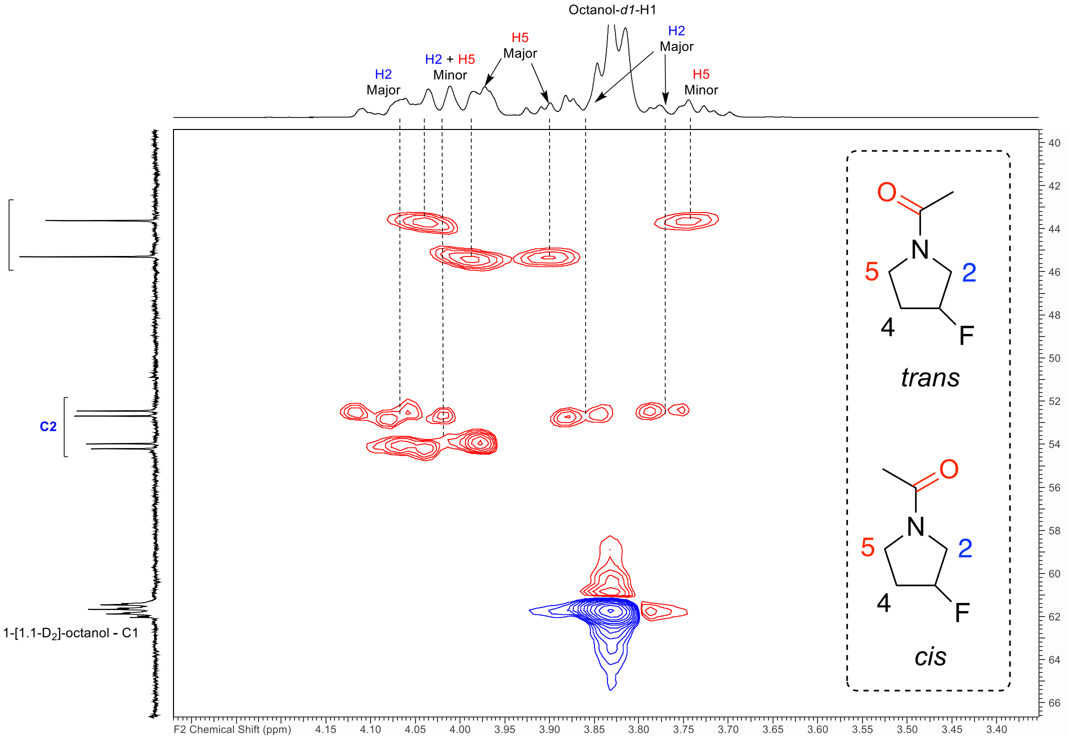

Figure S7: HSQC spectrum of N-acetyl-3-fluoropyrrolidine **2** in (1,1-D_2)-octan-1-ol.

Now, HSQC analysis could be carried out (**Figure S7**). C2 can be identified based on the $^2J_{C2\text{-}F}$ coupling constants (d, J = 23.5 Hz), while C5 can be recognised by its lack of multiplicity. The respective crosspeaks indicate the chemical shift values of the corresponding proton resonances.

In a NOESY spectrum a cross-peak will be observed for protons that are close in space. Therefore in the *trans*-rotamer, the acetate CH_3 will only show a cross-peak with H2 and while in the cis rotamer, the acetate CH_3 will only show a cross-peak with H5. In the spectra below H5 has a cross-peak with the major acetate signal, which confirms that the major rotamer has the *cis*-conformation (**Figure S8**). The opposite is expected – and observed – for H2, where H2 only shows a cross-peak with the minor acetate signal.

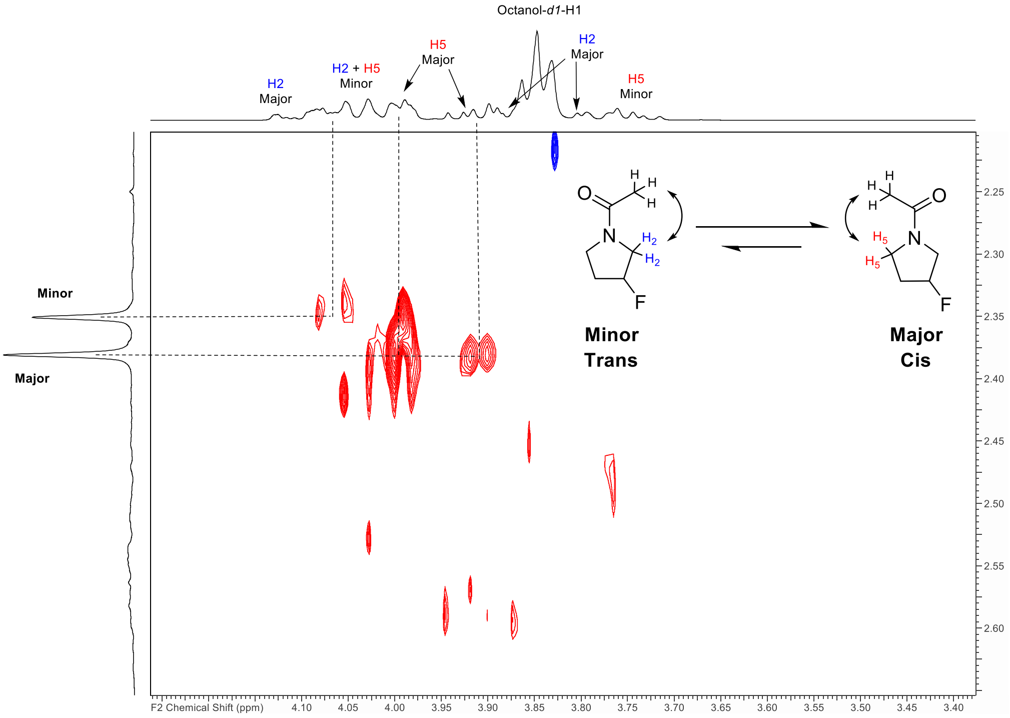

Figure S8. NOESY spectrum of *N*-acetyl-3-fluoropyrrolidine **2** in 1-(1,1-D_2)-octanol

4.2.2.3 Rotamer assignment of *N*-acetyl-3,3-difluoropyrrolidine **3** in octanol

HSQC analysis (**Figure S9**) in (1,1-D_2)-octan-1-ol (benzene-d_6 capillary) was carried out similarly to that of the previous example: C2 can be identified due to the $^2J_{C2\text{-}F}$ coupling constants (t, J = 32.3 Hz), while C5 can be recognised by its much smaller $^3J_{C5\text{-}F}$ coupling constants (t, J = 2.9 Hz). Once the carbons had been assigned, the major and minor signals of H2 and H5 can be assigned via the cross-peaks, clearly visible adjacent to the residual resonance of octanol, that is present as trace impurity in (1,1-D_2)-octan-1-ol.

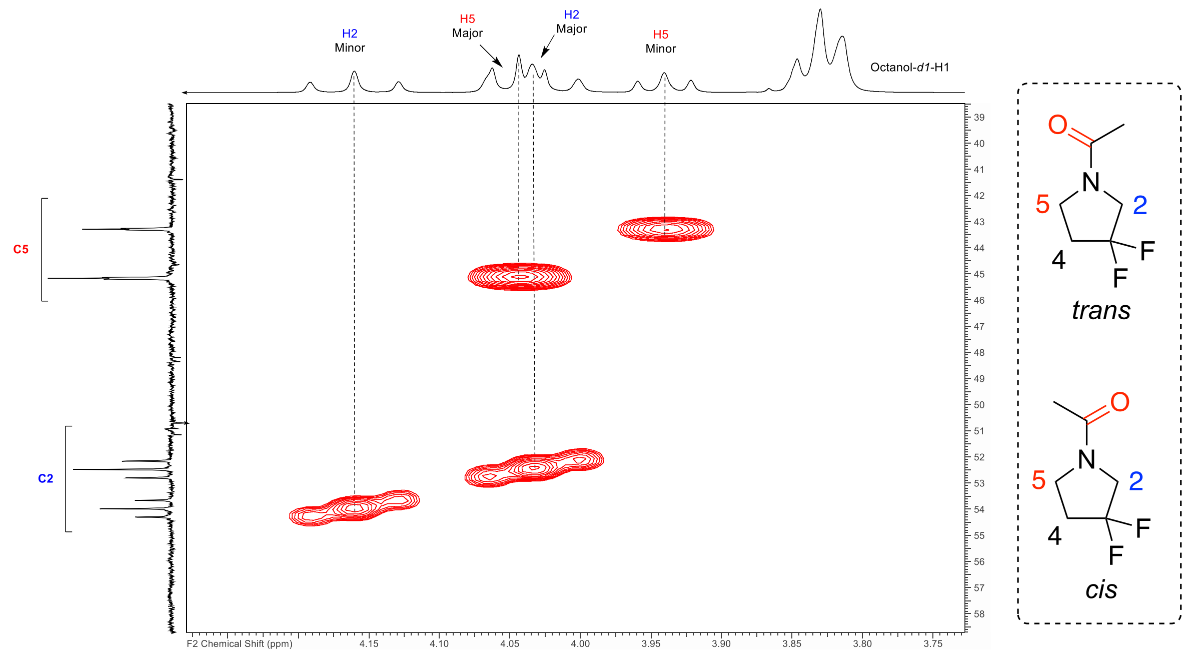

Figure S9. HSQC spectrum of N-acetyl-3,3-difluoropyrrolidine **3** in 1-(1,1-D_2)-octanol.

Despite the overlapping of the H2 and H5 resonances of the major rotamer in the ^{1}H NMR spectrum, the configuration of the rotamers can still be assigned via HSQC analysis (**Figure S10**). The acetate CH_3 resonance of the minor rotamer shows a cross-peak with an H2 signal, indicating that the minor confirmation has *trans*-configuration. Therefore it can be determined that the major acetate resonance is displaying a cross-peak to the H5 signal of the major rotamer, and not the H2 signal.
Thus, the major rotamer of **3** in octanol also has the *cis*-conformation.

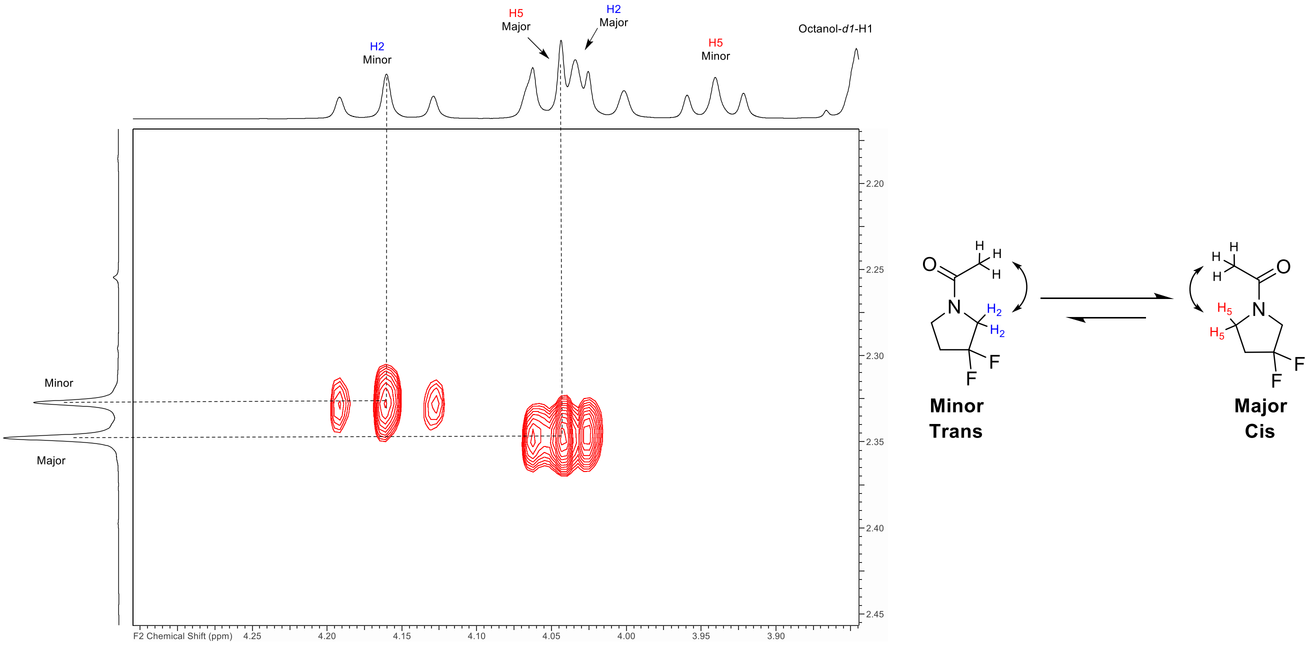

Figure S10. NOESY spectrum of N-acetyl-3,3-difluoropyrrolidine **3** in (1,1-D_2)-octan-1-ol.

4.2.2.4 Rotamer assignment of *N*-acetyl-3-fluoropiperidine **4** in octanol

The HSQC spectrum in (1,1-D_2)-octan-1-ol is shown in **Figure S11**. The correlation of the C6 resonance of the major rotamer with its two protons, which now have a different chemical shift, is easily seen. The other assignments are more difficult, but still clear, as indicated.

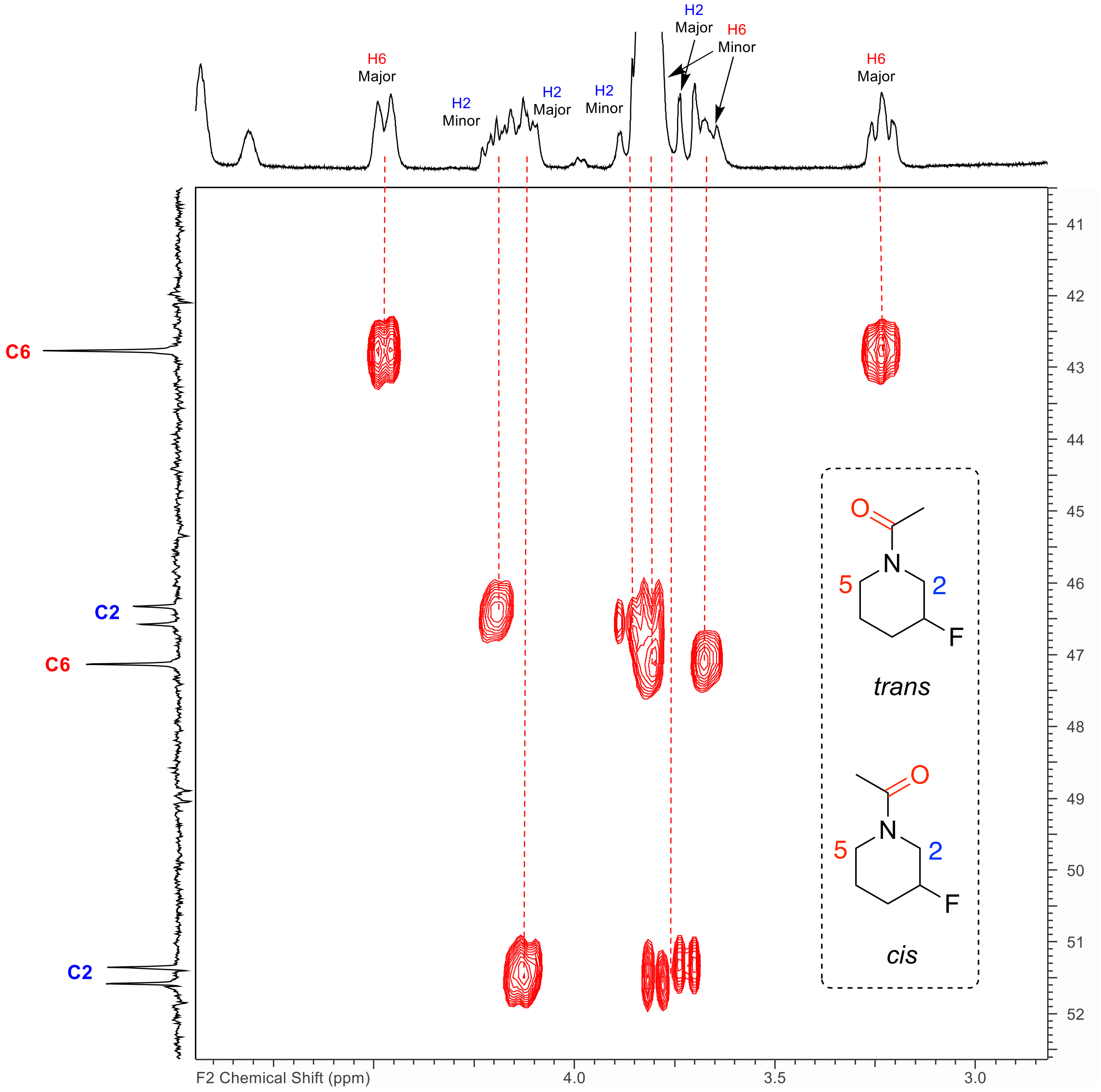

Figure S11. HSQC spectrum of N-acetyl-3-fluoropiperidine **4** in (1,1-D_2)-octan-1-ol.

In the NOESY spectrum (**Figure S12**), it is observed that the H6 signals of the major rotamer do not show a crosspeak to an acetate resonance. Hence, by inference the major rotamer must have the *trans*-conformation. This can be confirmed by inspection of the crosspeaks, despite the slight overlap of the acetate peaks: the major acetate peak shows a crosspeak to the H2 resonances of the major rotamer, while the minor acetate peak shows a crosspeak with the H6 resonances of the minor rotamer.
Hence the major rotamer for N-acetyl-3-fluoropiperidine **4** in octanol has the *trans*-conformation.

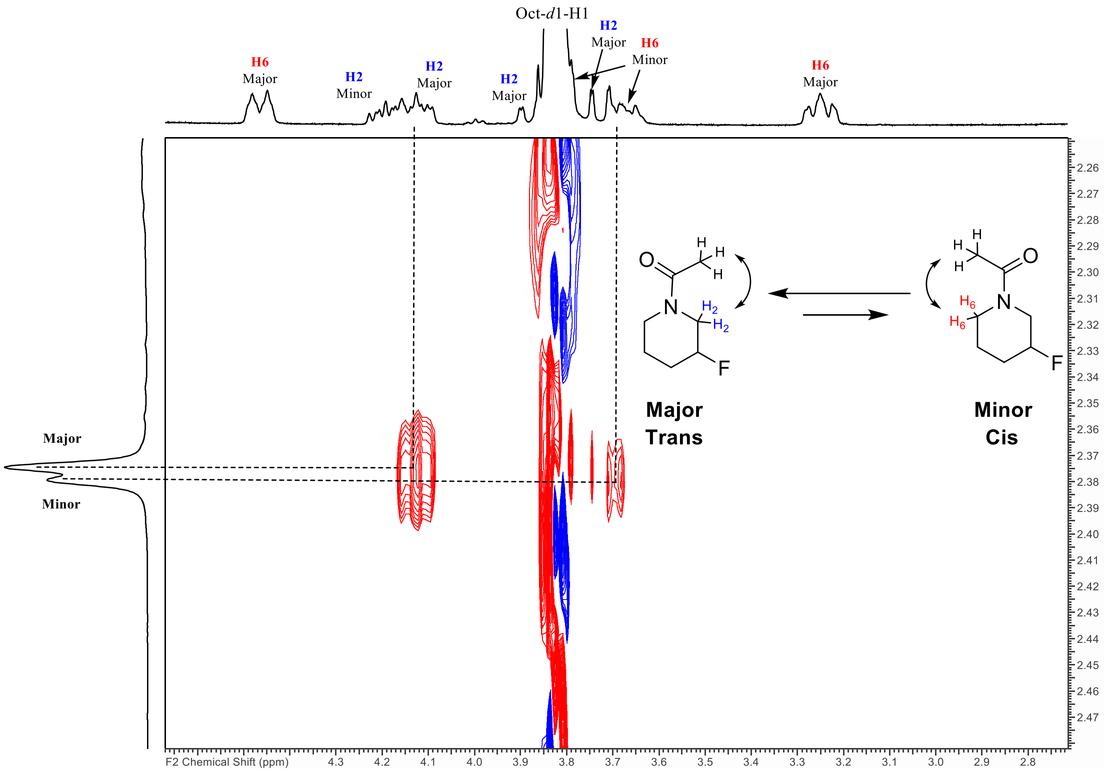

Figure S12. NOESY spectrum of *N*-acetyl-3-fluoropiperidine **4** in (1,1-D_2)-octan-1-ol.

4.2.2.5 Rotamer assignment of *N*-acetyl-3,3-difluoropiperidine **5** in octanol

The HSQC spectrum in (1,1-D_2)-octan-1-ol is shown in **Figure S13**. Similar to that of previous examples the C2 can be identified due to $^2J_{C2\text{-}F}$ coupling constants (t, J = 31.5 Hz), while C6 can be recognised by its lack of multiplicity. The respective crosspeaks indicate the chemical shift values of the corresponding proton resonances, with the minor H6 partially overlapping with residual oct-*d1*-H1.

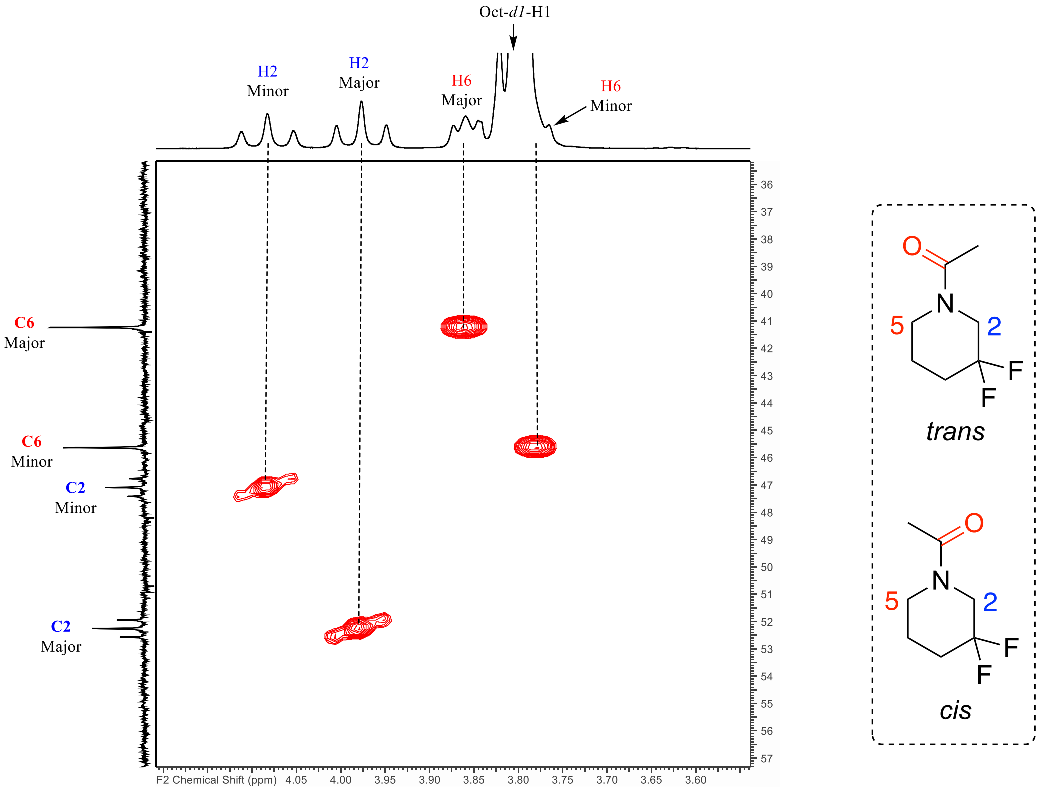

Figure S13. HSQC spectrum of *N*-acetyl-3-fluoropiperidine **5** in (1,1-D_2)-octan-1-ol.

In this case a ROESY experiment was used because of t_1 noise in the NOESY spectrum from the residual oct-d_1-H1 signal, causing difficulty in accurately assigning cross peaks. In the ROESY spectrum (**Figure S14**), it can be seen that the major H2 signal shows a cross-peak with the corresponding major acetate resonance. In the case of the minor acetate resonance, a cross-peak can be observed with the minor H6 signal which partially overlaps with the oct-*d1*-H1.

Hence the major rotamer for *N*-acetyl-3,3-difluoropiperidine **5** in octanol has the *trans*-conformation.

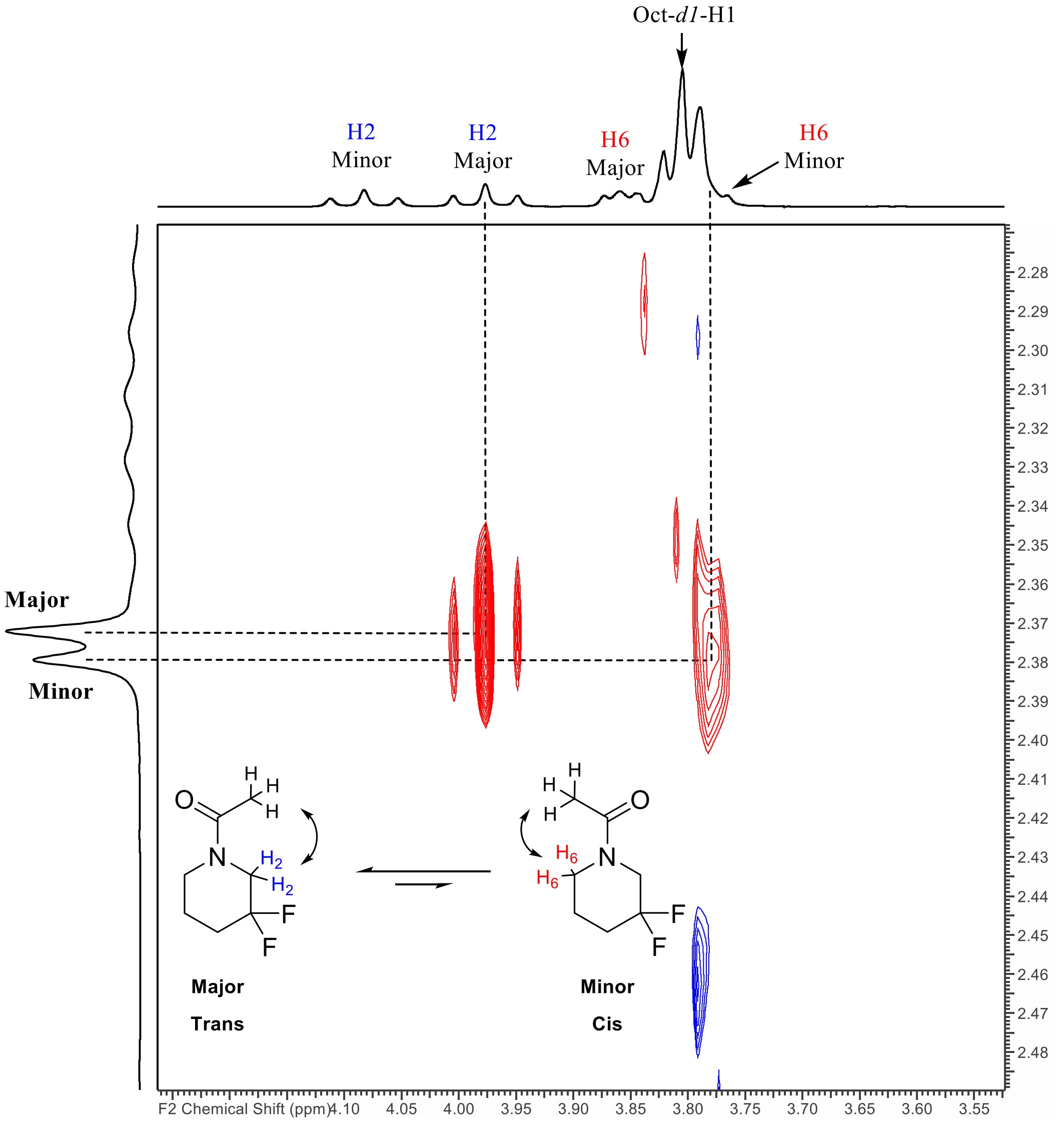

Figure S14. ROESY spectrum of N-acetyl-3-fluoropiperidine **5** in (1,1-D_2)-octan-1-ol.

4.2.2.6 Rotamer assignment of *N*-acetyl-(2*S*,4*R*)-4-fluoroproline dimethyl amide **6b** in octanol

The HSQC spectrum in 1-(1,1-D_2)-octanol is shown in **Figure S15**. Due to the fluorine-fluorine coupling and high rotamer ratio of ~1:0.12, only the major rotamer is easily observed in the $^{13}C\{^1H\}$ NMR spectrum. To add further complications, the C2 and C5 major also overlap. Despite this C5 can be identified due to the $^2J_{C5\text{-}F}$ coupling constant (d, J = 22.0 Hz), while C2 can be recognised by its lack of multiplicity. H2 can be further confirmed by its characteristic coupling to protons H3 $^2J_{H2\text{-}H3}$ (t, J = 8.3). The respective crosspeaks indicate the chemical shift values of the corresponding proton resonances, with the minor H2 partially overlapping with residual oct-*d2*-O**H** signal.

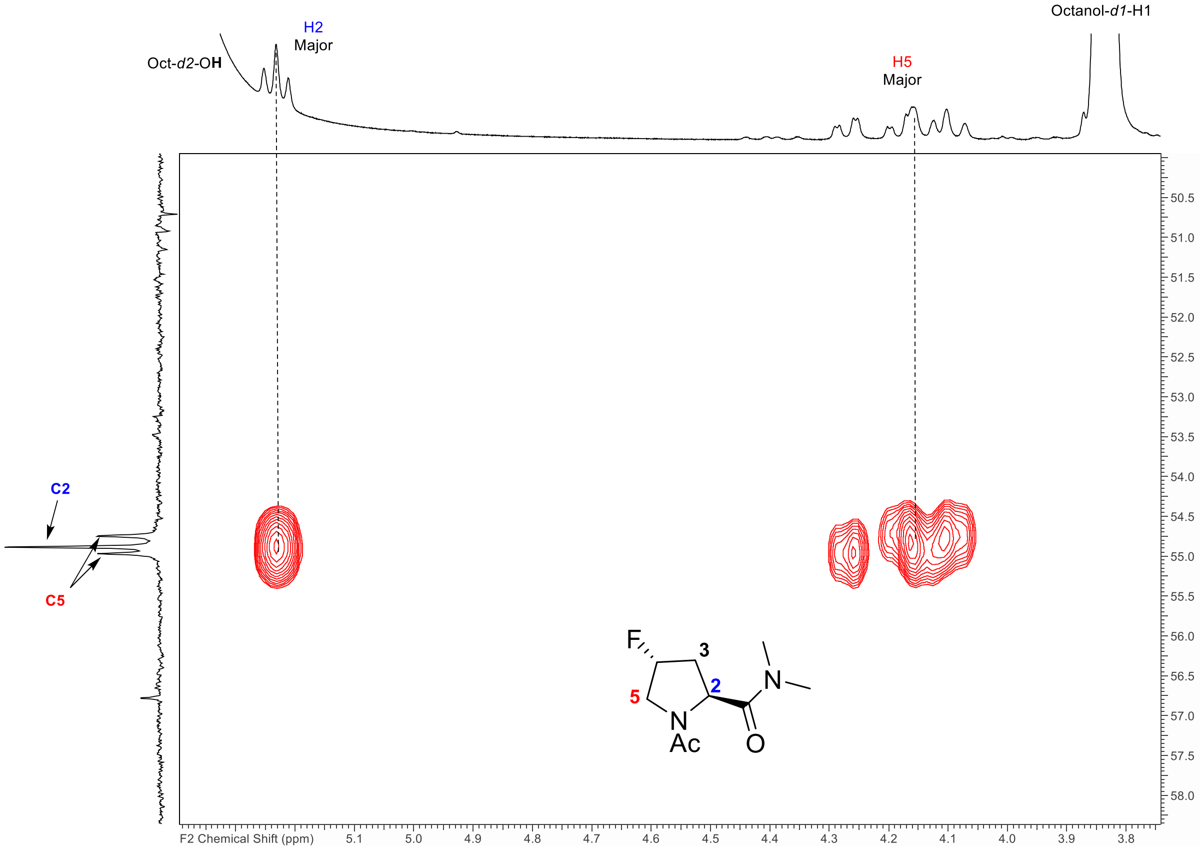

Figure S15. HSQC spectrum of N-acetyl-(2*S*,4*R*)-4-fluoroproline dimethyl amide **6b** in (1,1-D_2)-octan-1-ol.

In the NOESY spectrum (**Figure S16**), the major H5 signal shows a clear crosspeak with the corresponding major acetate resonance. In the case of the minor acetate resonance no cross peak can be observed, as the minor H2 signal was not identified through HSQC analysis and is likely to be within the oct-d2-OH signal. Hence through analysis of the major acetate resonance alone, the major rotamer for *N*-acetyl-(2*S,4R*)-4-fluoroproline dimethyl amide **6b** in octanol is established as the *trans*-conformation.

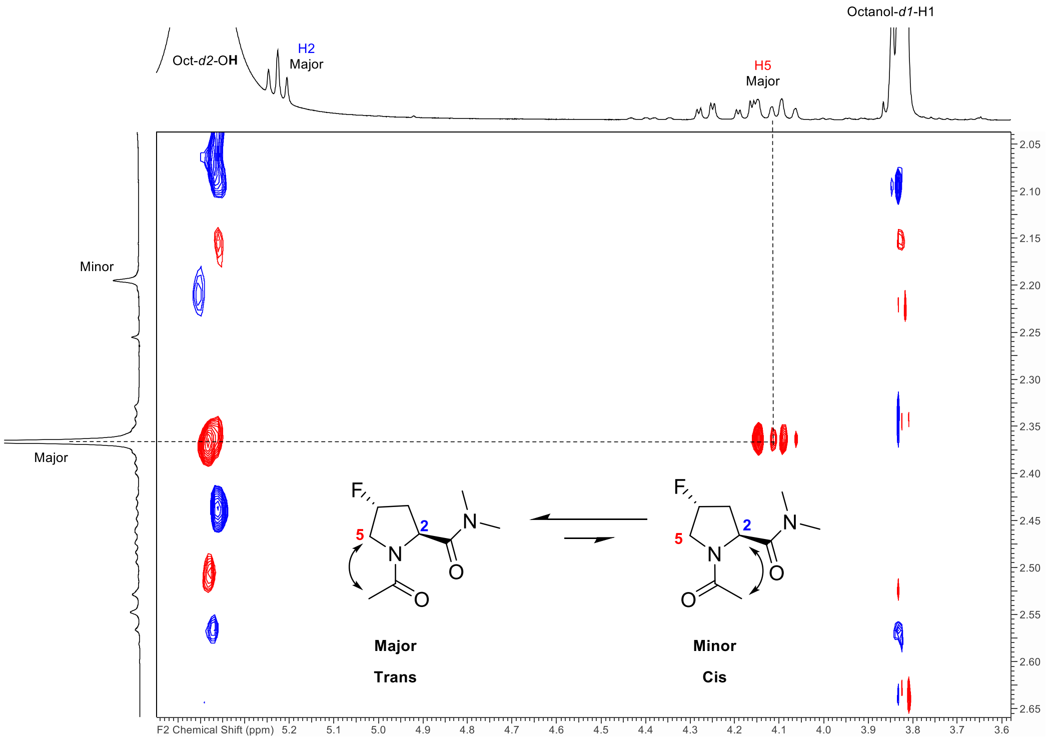

Figure S16. NOESY spectrum of N-acetyl-(2*S*,4*R*)-4-fluoroproline dimethyl amide **6b** in (1,1-D_2)-octan-1-ol.

4.2.2.7 Rotamer assignment of *N*-acetyl-(2*S*)-4,4-difluoroproline dimethyl amide **7b**

The HSQC spectrum in (1,1-D_2)-octan-1-ol is shown in **Figure S17**. Due to the fluorine-fluorine coupling and high rotamer ratio of ~1:0.19, only the major rotamer is easily observed in the $^{13}C\{^1H\}$ NMR spectrum. To add further complications, the C2 and C5 major also partially overlap (see **Figure S18** for expansion of signals). Despite this the C5 can be identified due to the $^2J_{C5-F}$ coupling constants (dd, J = 30.4, 34.1 Hz), while C2 can be recognised by its much smaller $^3J_{C2-F}$ coupling constants (d, J = 2.9 Hz). The respective crosspeaks indicate the chemical shift values of the corresponding proton resonances, with the minor H2 partially overlapping with residual oct-*d2*-OH signal.

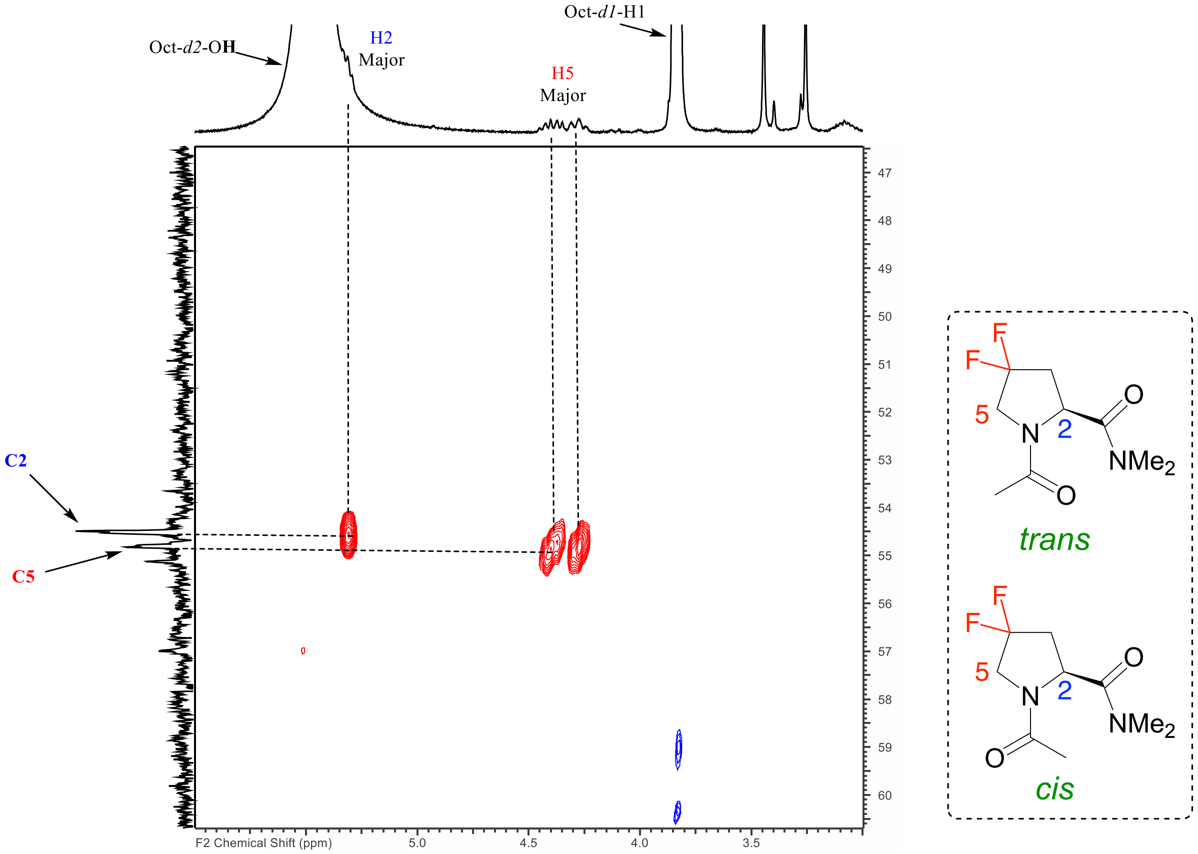

Figure S17. HSQC spectrum of N-acetyl-(2*S*)-4,4-difluoroproline dimethyl amide **7b** in (1,1-D_2)-octan-1-ol.

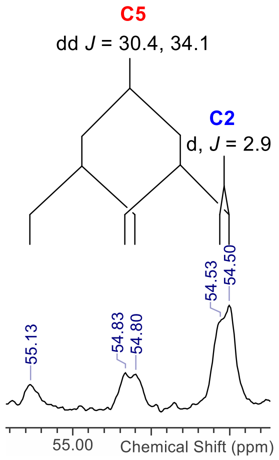

Figure S18. Expanded ^{13}C spectrum of C5 and C2 major for N-acetyl-(2*S*)-4,4-difluoroproline dimethyl amide **7b** in (1,1-D_2)-octan-1-ol.

In the NOESY spectrum (**Figure S19**), the major H5 signal shows a clear crosspeak with the corresponding major acetate resonance. In the case of the minor acetate resonance no cross peak can be observed, as the minor H2 signal was not identified through HSQC analysis and is likely to be within the oct-d2-OH signal. Hence through analysis of the major acetate resonance alone, the major rotamer for *N*-acetyl-(2*S*)-4,4-difluoroproline dimethyl amide **7b** in octanol is established as the *trans*-conformation.

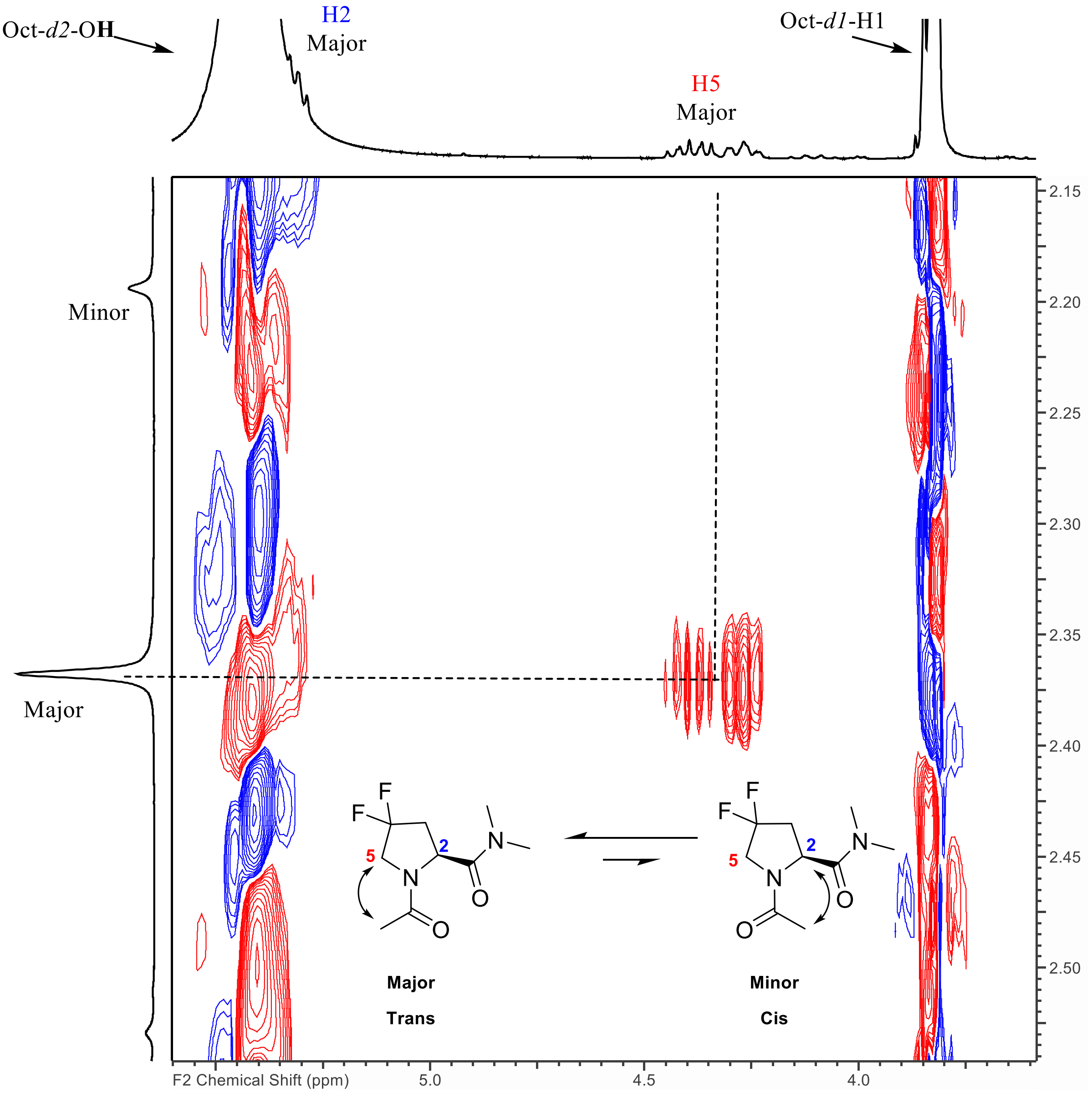

Figure S19. NOESY spectrum of N-acetyl-(2*S*)-4,4-difluoroproline dimethyl amide **7b** in (1,1-D_2)-octan-1-ol.

4.3 Detailed experimental data for the log*P* determinations (ρ_{oct} and ρ_{wat} values for each experiment)

4.3.1 Standard NMR settings

Standard NMR parameter setting: SW, 300 ppm; centered O1P; NS 64; D1 30 sec (octanol sample), D1 60 sec (water sample). Any change from standard setting was described for each compound. Unless specified otherwise 2,2,2-trifluoroethanol (log*P*: +0.36) was used as the reference compound.

4.3.2 N-Acetyl-3-fluoropyrrolidine **2**

log*P*
Reference - 1-Fluoropropan-2-ol

Compound	Experimental (octanol/water)	ρ_{oct}/ρ_{wat}	Log*P*	Average Log*P*	Error
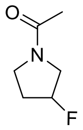	my2716bj6/ my2716b7	0.2483/0.4086	-0.57	-0.57	-0.568 (±0.002)
	my2716bj8/ my2716b9	0.5189/0.8615	-0.57		
	my2716bj10/ my2716b11	0.2140/0.3520	-0.57		

Ratio of *E*:*Z*-rotamers, and *K* value:

Octanol phase	Water phase
0.75:1 (1.33)	0.86:1 (1.16)

Conformer Specific Log*p*

Conformation	ρ_{oct}/ρ_{wat}	Log*P*	Average Log*P*	Error
E	0.1062/0.1892	-0.60	-0.60	-0.602 (±0.001)
	0.2228/0.3985	-0.60		
	0.0908/0.1631	-0.60		
Z	0.1424/0.2194	-0.54	-0.54	-0.538 (±0.003)
	0.2967/0.4626	-0.54		
	0.1234/0.1887	-0.53		

4.3.3 *N*-Acetyl-3,3-difluoropyrrolidine **3**

Compound	Experimental (octanol/water)	ρ_{oct}/ρ_{wat}	Log*P*	Average Log*P*	Error
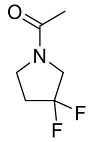	ju1016bj9/ ju1016bj10	0.0798/0.2410	-0.12		
	ju1016bj11/ ju2916bj4	0.1373/0.4144	-0.12	-0.12	-0.117 (±0.004)
	ju1016bj13/ ju1016bj14	0.0975/0.2887	-0.11		

Ratio of *E*:*Z*-rotamers, and *K* value:

Octanol phase	Water phase
0.72/1 (1.39)	0.84/1 (1.19)

Conformer Specific Log*p*

Conformation	ρ_{oct}/ρ_{wat}	Log*P*	Average Log*P*	Error
	0.0330/0.1093	-0.16	-0.15	
E	0.0579/0.1895	-0.15		-0.153 (±0.006)
	0.0411/0.1314	-0.15		
	0.0468/0.1319	-0.09		
Z	0.0794/0.2249	-0.09	-0.09	-0.089 (±0.003)
	0.0411/0.1314	-0.09		

4.3.4 *N*-Acetyl-3-fluoropiperdine **4**

Reference - 4-Fluorobutan-1-ol

Compound	Experimental (octanol/water)	ρ_{oct}/ρ_{wat}	Log*P*	Average Log*P*	Error
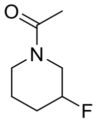	oc0616bj3/ oc0616bj4	1.9574/1.3281	-0.09		
	oc0616bj5/ oc0716bj3	1.5753/1.1101	-0.11	-0.10	-0.100 (±0.008)
	oc0616bj7/ oc0716bj2	2.6861/1.8871	-0.11		

Ratio of *E*:*Z*-rotamers, and *K* value:

Octanol phase	Water phase
1.43/1 (0.70)	0.95/1 (1.05)

Conformer Specific Log*p*

Conformation	ρ_{oct}/ρ_{wat}	Log*P*	Average Log*P*	Error
	1.1397/0.6440	-0.01	-0.02	
E	0.9366/0.5367	-0.02		-0.015 (±0.003)
	1.5800/0.9229	-0.03		
	0.8177/0.6841	-0.18		
Z	0.6387/0.5734	-0.21	-0.20	-0.198 (±0.015)
	1.1061/0.9642	-0.20		

4.3.5 *N*-Acetyl-3,3-difluoropiperdine **5**

Compound	Experimental (octanol/water)	ρ_{oct}/ρ_{wat}	Log*P*	Average Log *P*	Error
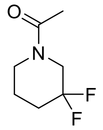	ju2916bj2/ ju2916bj3	0.5618/0.6711	+0.28	+0.27	+0.272 (±0.007)
	ju2916bj4/ ju2916bj5	0.4532/0.5554	+0.27		
	ju2916bj6/ ju2916bj7	0.7613/0.9494	+0.26		

Ratio of *E*:*Z*-rotamers, and *K* value:

Octanol phase	Water phase
1.17/1 (0.85)	0.73/1 (1.37)

Conformer Specific Log*p*

Conformation	ρ_{oct}/ρ_{wat}	Log*P*	Average Log*P*	Error
E	0.3027/0.2843	+0.39	+0.38	+0.378 (±0.008)
	0.2456/0.2340	+0.38		
	0.4097/0.4027	+0.37		
Z	0.2591/0.3868	+0.18	+0.17	+0.174 (±0.008)
	0.2076/0.3214	+0.17		
	0.3516/0.5467	+0.17		

4.3.6 N-acetyl-4*R*-FPro-OMe **6a**

Reference: 2-Fluoroethanol. Change from standard in NMR parameter setting: SW (160 ppm); octanol sample, NS (1024); water sample, NS (512). Capillary tubes containing acetone-d_6 were used for signal lock (NMR experiments).

Compound (Ref: ZW8456-85)	Experimental (octanol/water)	ρ_{oct}/ρ_{wat}	Log*P*	Average Log*P*	Error
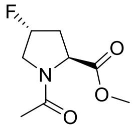	au2418zw3/au2418zw4	0.5724/0.4087	-0.604	-0.60	-0.6045 (±0.005)
	au2418zw7/au2418zw8	0.5717/0.4098	-0.605		

Ratio of trans/cis-rotamers, ($K_{t/c}$)

Octanol phase	Water phase
5.29/1	6.49/1

Conformer Specific Log*P*

Conformation	ρ_{oct}/ρ_{wat}	Log*P*	Average Log*P*	Error
Trans	0.4802/0.3547	-0.618	-0.62	-0.618 (±0.001)
	0.4818/0.3544	-0.617		
Cis	0.0922/0.0540	-0.518	-0.53	-0.529 (±0.011)
	0.0899/0.0554	-0.540		

4.3.7 N-acetyl-4*R*-FPro-NMe_2 **6b**

Reference - 2-fluoroethanol

Compound	Experimental (octanol/water)	ρ_{oct}/ρ_{wat}	Log*P*	Average Log*P*	Error
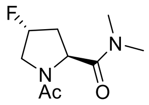	jy0618bj6/ jy0318bj16	0.3721/0.3524	-1.17	-1.16	-1.161 (±0.009)
	jy0618bj7/ jy0318bj18	0.3524/0.9124	-1.16		
	jy0618bj8/ jy0318bj20	0.5626/1.4096	-1.15		

Ratio of trans/cis-rotamers, and *K* value

Octanol phase	Water phase
8.29/1	4.44/1

Conformer Specific Log*P*

Conformation	ρ_{oct}/ρ_{wat}	Log*P*	Average Log*P*	Error
Trans	0.3319/0.7982	-1.13	-1.12	-1.122 (±0.009)
	0.3139/0.7447	-1.13		
	0.5033/1.1538	-1.11		
Cis	0.0402/0.1823	-1.41	-1.39	-1.393 (±0.009)
	0.0385/0.1677	-1.39		
	0.0593/0.2558	-1.38		

4.3.8 N-Acetyl-4,4-F_2Pro-OMe **7a**

Reference: 2,2,2-trifluoroethanol. Change from standard in NMR parameter setting: SW (140 ppm); octanol sample, D1 (15 sec), NS (1024); water sample, D1 (30 sec), NS (1536). Capillary tubes containing acetone-d_6 were used for signal lock (NMR experiments).

Compound (Ref: ZW8456-91)	Experimental (octanol/water)	ρ_{oct}/ρ_{wat}	Log*P*	Average Log*P*	Error
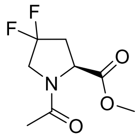	au3118zw5/au3118zw6	0.2427/0.8739	-0.196	-0.20	-0.196 (±0.001)
	au3118zw7/au3118zw8	0.2394/0.8616	-0.196		
	au3118zw9/au3118zw10	0.2377/0.8572	-0.197		

Ratio of trans/cis-rotamers (*K*)

Octanol phase	Water phase
2.09/1	3.48/1

Conformer Specific Log*P*

Conformation	ρ_{oct}/ρ_{wat}	Log*P*	Average Log*P*	Error
Trans	0.0822/0.3401	-0.257	-0.26	-0.256 (±0.001)
	0.0813/0.3350	-0.255		
	0.0803/0.3327	-0.257		
Cis	0.0395/0.0977	-0.033	-0.04	-0.035 (±0.001)
	0.0386/0.0961	-0.036		
	0.0386/0.0961	-0.036		

4.3.9 N-Acetyl-4,4-F_2Pro-NMe_2 **7b**

Reference - 2,2,3,3-tetrafluoro-1,4-butanediol

Compound	Experimental (octanol/water)	ρ_{oct}/ρ_{wat}	Log*P*	Average Log*P*	Error
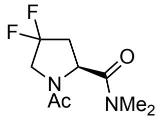 **9b**	jy1318bj20/ jy1318bj20	0.5739/3.5040	-0.90	-0.89	-0.888 (±0.007)
	au0318bj3/ au0318bj4	0.5979/3.5360	-0.88		
	au0318bj5/ au0318bj6	0.7126/4.2510	-0.89		

Ratio of trans/cis-rotamers

Octanol phase	Water phase
4.76/1 (0.21)	3.68/1 (0.27)

Conformer Specific Log*P*

Conformation	ρ_{oct}/ρ_{wat}	Log*P*	Average Log*P*	Error
Trans	0.4750/2.7508	-0.87	-0.87	-0.866 (±0.005)
	0.4939/2.7818	-0.86		
	0.5885/3.3455	-0.86		
Cis	0.0989/0.7532	-0.99	-0.98	-0.978 (±0.009)
	0.1040/0.7542	-0.97		
	0.1241/0.9055	-0.97		

5 Conformational analysis

5.1 General

All the calculations were performed with the Gaussian16 program[2] applying default procedures, integration grids, algorithms and parameters. The conformational analysis of compounds **2**-**9** was carried out with the MN15 functional[3] and the triple-zeta quality cc-pVTZ basis set. According to the chemical structure of the compounds, relevant five or six membered ring conformers, *E* and *Z* isomers for amides, were systematically considered. For monofluorinated pyrrolidines and piperidines, the conformers with the fluorine atom in axial or equatorial positions were also investigated. The solvent effects (octanol and water) were taken into account using the SMD solvation continuum model.[4] The vibrational frequencies of each optimized conformer were computed to confirm its nature of true minimum (no imaginary frequencies) and to obtain the required terms to get the free energies at the SMD/MN15/cc-pVTZ level of theory. Single point calculations at the SMD/MN15/aug-cc-pVTZ were finally carried out to obtain refined electronic energy values. The relative populations, p_i, of the various conformers were evaluated at 298K from the computed free energies through a Boltzmann distribution (Eq. (1)).

$$p_i = \frac{e^{-\Delta G_i/RT}}{\sum_{i=1}^{n} e^{-\Delta G_i/RT}} \quad (1)$$

The theoretical molecular dipole moments were computed for each conformer, and were weighted according to the previous relevant populations.

5.2 Pyrrolidines and piperidines **2–5**

The corresponding results are reported in Tables **1-7**, and the optimized structures of the energetic minima in *n*-octanol are illustrated in Figures 1-7, those in water being very similar.

5.2.1 N-Acetyl-3-fluoropyrrolidine **2**

There are four minimum-energy conformations in **2**, the amide *E* vs. *Z* and a ring conformation with *ax*-F vs. *eq*-F conformers (**Figure S20**).

Figure S20. Optimized structures of the conformers of *N*-acetyl-3-fluoropyrrolidine **2** in *n*-octanol medium. The dipole moment vectors are shown in blue.

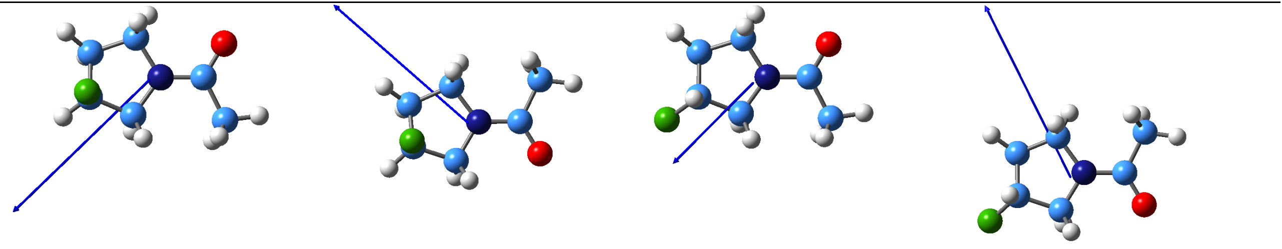

(*E*,*ax*)	(*Z*,*ax*)	(*E*, *eq*)	(*E*, *eq*)

In both octanol and water, the conformers of both *E* or *Z* isomers with the fluorine in axial position are found to be significantly preferred over these with the C–F in equatorial position (Table S1), as expected from the fluorine gauche effect. This leads to the C–F dipole to be almost perpendicular to the amide dipole (**Figure S21**). The experimentally determined *Z/E* ratios match the computational values, although the value in octanol was underestimated.

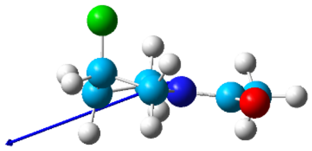

Figure S21. Side-view of the **2ax** conformation.

Table S1. Relative Gibbs energy populations and dipole moments of the energetic minima of *N*-acetyl-3-fluoropyrrolidine **2** identified at the SMD/MN15/aug-cc-pVTZ//MN15/cc-pVTZ level of theory in water and octanol medium.

	Octanol				Water			
	μ	ΔG	p_i	$p_{i,exp}$	μ	ΔG	p_i	$p_{i,exp}$
Conformer	D	kJ mol^{-1}	%	%	D	kJ mol^{-1}	%	%
ax-E	6.45	0.3	45.8	-	7.04	0.2	46.4	-
eq-E	3.66	8.8	1.5	-	4.12	9.1	1.3	-
E	**6.36**		**47.3**	**42.9**	**6.96**		**47.6**	**46.2**
ax-Z	6.19	0.0	51.4	-	6.76	0.0	51.0	-
eq-Z	6.40	9.1	1.3	-	6.95	9.1	1.3	-
Z	**6.20**		**52.7**	**57.1**	**6.76**		**52.4**	**53.8**
**Ratio *Z*/*E***	**0.97**		**1.11**	**1.33**	**0.97**		**1.10**	**1.16**
**Ratio *E*/*Z***	**1.03**		**0.90**	**0.75**	**1.03**		**0.91**	**0.86**

5.2.2 *N*-Acetyl-3,3-difluoropyrrolidine **3**

For compound **3**, two different minimum-energy conformers are found (**Figure S22**), the twist inversion of the pyrrolidine ring leading to equivalent conformations.

Figure S22. Optimized structures of the conformers of *N*-acetyl-3,3-difluoropyrrolidine **3** in *n*-octanol medium. The dipole moment vector is shown in blue.

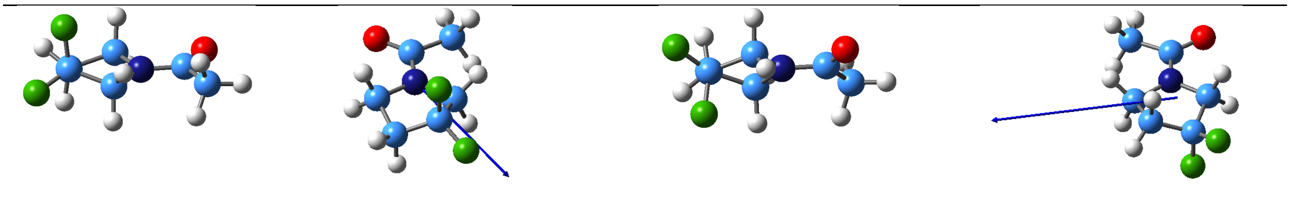

(*E*) (*Z*)

The *E*-isomer is calculated to be the major isomer in octanol, and with similar populations in water (**Table S2**). This does not correspond with the experimental values, which show the *Z*-isomer as the major isomer in both phases.

Table S2. Relative Gibbs energy, populations and dipole moments of the energetic minima of *N*-acetyl-3,3-difluoropyrrolidine **3** identified at the SMD/MN15/aug-cc-pVTZ//MN15/cc-pVTZ level of theory in water and octanol medium.

	Octanol				Water			
	μ	ΔG	p_i	$p_{i,exp}$	μ	ΔG	p_i	$p_{i,exp}$
Conformer	D	kJ mol^{-1}	%	%	D	kJ mol^{-1}	%	%
E	**3.83**	0.00	55.9	42	**4.29**	0.00	49.7	46
Z	**6.20**	0.59	44.1	58	**6.72**	-0.02	50.3	54
**Ratio *Z*/*E***	**1.62**		0.78	1.4	**1.57**		1.01	1.2
**Ratio *E*/*Z***	**0.62**		1.27	0.72	**0.64**		0.99	0.84

Hence, this leads to the more polar *Z*-isomer is also the most lipophilic, albeit only just.

5.2.3 *N*-Acetyl-3-fluoropiperidine **4**

There are four minimum-energy conformations in **4**, the amide *E* vs. *Z* and a ring conformation with *ax*-F vs. *eq*-F conformers (**Figure S23**).

Figure S23. Optimized structures of the conformers of *N*-acetyl-3-fluoropiperidine **4** in *n*-octanol medium. The dipole moment vector is shown in blue.

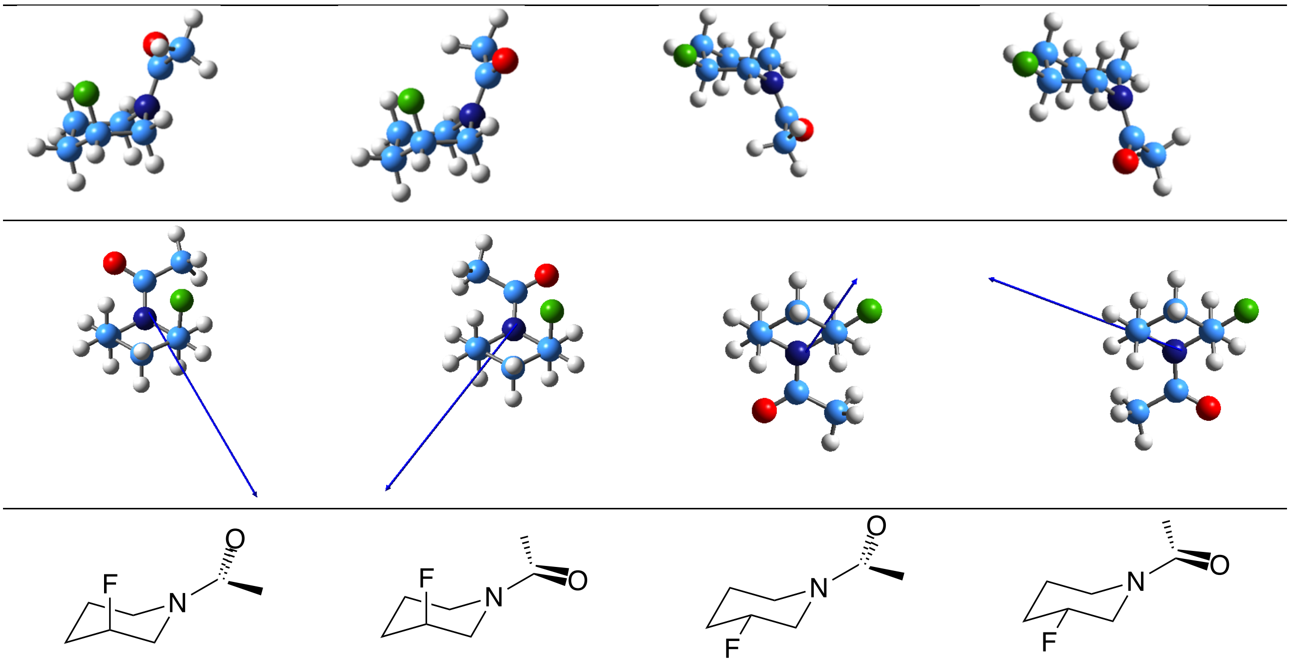

(*E*,*ax*) (*Z*,*ax*) (*E*, *eq*) (*E*, *eq*)

The conformers of both *E* or *Z* isomers with the fluorine in axial position are calculated to be preferred over those with the C–F in equatorial position (Table S3), in accordance with the fluorine gauche effect. The equatorial conformers are much more stabilized in octanol compared to water.

Table S3. Distribution of the conformer populations of compound **4** according to chair conformation. Relative Gibbs energy populations and dipole moments of the energetic minima of *N*-acetyl-3-fluoropiperidine identified at the SMD/MN15/aug-cc-pVTZ//MN15/cc-pVTZ level of theory in water and octanol medium.

	**Octanol**			**Water**		
	μ	ΔG	p_i	μ	ΔG	p_i
Conformer	D	kJ mol^{-1}	%	D	kJ mol^{-1}	%
ax-E	6.90	0.0	34.4	7.56	0.0	43.3
ax-Z	7.40	0.9	24.3	8.11	0.4	36.5
ax	**7.10**		**58.7**	**7.81**		**79.8**
eq-E	3.12	1.0	23.2	3.45	4.0	8.7
eq-Z	6.93	1.6	18.1	7.44	3.3	11.5
eq	**4.79**		**41.3**	**5.73**		**20.2**
**Ratio ax/eq**	**1.48**		**1.42**	**1.36**		**3.95**

It is worth mentioning that the boat conformations of **4** have also been investigated. In water, a strong destabilization is found for these structures compared to the chair ones (at least 49 kJ mol^{-1}), and the corresponding populations are therefore negligible. In octanol, the energetic difference is weaker (from 10 to 20 kJ mol^{-1}) but the corresponding populations are still very low (at the best 0.5% for the boat_ax_Z conformer).

Interestingly, the *ax-E*-rotamer is found to be slightly more stable than the *ax-Z*-rotamer.

Table S4. Relative Gibbs energy populations and dipole moments of the energetic minima of *N*-acetyl-3-fluoropiperidine **4** identified at the SMD/MN15/aug-cc-pVTZ//MN15/cc-pVTZ level of theory in water and octanol medium.

	Octanol				Water			
Conformer	μ D	ΔG kJ mol^{-1}	p_i %	$p_{i,exp}$ %	μ D	ΔG kJ mol^{-1}	p_i %	$p_{i,exp}$ %
ax-E	6.90	0.0	34.4		7.56	0.0	43.3	
eq-E	3.12	1.0	23.2		3.45	4.0	8.7	
E	**5.37**		**57.6**	**59**	**6.87**		**52.0**	**49**
ax-Z	7.40	0.9	24.3		8.11	0.4	36.5	
eq-Z	6.93	1.6	18.1		7.44	3.3	11.5	
Z	**7.20**		**42.4**	**41**	**7.95**		**48.0**	**51**
**Ratio *Z*/*E***	**1.34**		**0.73**	**0.70**	**1.16**		**0.92**	**1.05**
**Ratio *E*/*Z***	**0.75**		**1.36**	**1.43**	**0.86**		**1.08**	**0.95**
**Ratio Z_{ax}/E_{ax}**	**1.07**		**0.71**		**1.07**		**0.84**	
**Ratio Z_{eq}/E_{eq}**	**2.22**		**0.78**		**2.15**		**1.32**	

5.2.4 *N*-Acetyl-3,3-difluoropiperidine **5**

For compound **5**, two different minimum-energy conformers are found (**Figure S24**).

Figure S24. Optimized structures of the conformers of *N*-acetyl-3,3-difluoropiperidine **5** in *n*-octanol medium. The dipole moment vector is shown in blue.

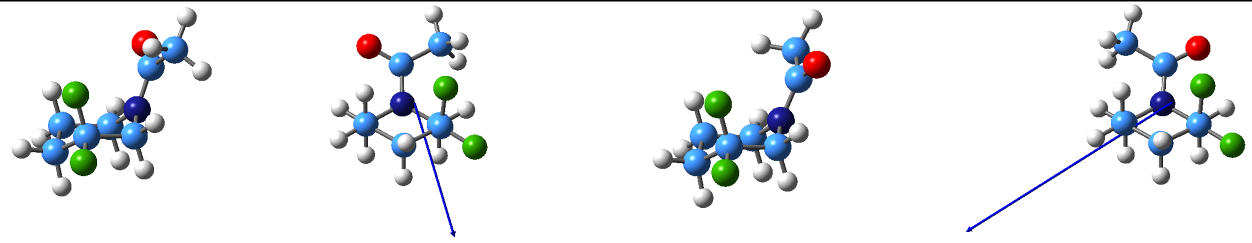

(*E*) (*Z*)

Table S5. Relative Gibbs energy, populations and dipole moments of the energetic minima of *N*-acetyl-3,3-difluoropiperidine **5** identified at the SMD/MN15/aug-cc-pVTZ//MN15/cc-pVTZ level of theory in water and octanol medium.

	Octanol				Water			
Conf.	μ D	ΔG kJ mol^{-1}	p_i %	$p_{i,exp}$ %	μ D	ΔG kJ mol^{-1}	p_i %	$p_{i,exp}$ %
E	**4.63**	0.00	58.2	54	**5.19**	0.00	49.6	42
Z	**8.00**	0.80	41.8	46	**8.73**	-0.04	50.4	58
**Ratio *Z*/*E***	**1.73**		0.72	0.85	**1.72**		1.02	1.37
**Ratio *E*/*Z***	**0.58**		1.39	1.17	**0.58**		0.98	0.73

The very similar free energy of solvation in water and equal calculated populations are not in agreement with the experiment: the measured *Z*/*E* ratio in water is 1.37. With the higher *Z*-rotamer polarity, this *Z*/*E* ratio drops considerably in octanol (0.85), which corresponds to the calculations.

It is worth mentioning that the boat conformations of **5** show a strong destabilization towards the chair ones (at least 48 kJ mol^{-1}), the corresponding populations being therefore negligible in both octanol and water.

5.3 Conformational analysis of the proline derivatives

5.3.1 N-Acetyl-(4*R*)-FPro-OMe **6a**

Table S6. Relative Gibbs energy, populations and dipole moments of the energetic minima of *N*-acetyl-(4*R*)-FPro-OMe **6a** identified at the SMD/MN15/aug-cc-pVTZ//MN15/cc-pVTZ level of theory in water and octanol medium

Conformer	Octanol μ (D)	Octanol ΔG (kJ mol^{-1})	Octanol p_i (%)	Octanol $p_{i,exp}$ (%)	Water μ (D)	Water ΔG (kJ mol^{-1})	Water p_i (%)	Water $p_{i,exp}$ (%)
t-endo ψ1	2.82	8.2	2.0		3.33	10.4	0.9	
t-exo ψ1	5.02	0.0	55.0		5.62	0.0	60.0	
t-endo ψ2	5.74	10.8	0.7		6.40	12.1	0.4	
t-exo ψ2	8.47	2.0	24.3		9.30	2.3	23.5	
trans	**6.00**		**82.0**	**84.1**	**6.62**		**84.9**	**86.6**
c-endo ψ1	8.14	11.1	0.6		8.86	10.9	0.7	
c-exo ψ1	7.31	3.5	13.6		8.06	4.1	11.7	
c-endo ψ2	3.81	13.4	0.3		4.04	14.6	0.2	
c-exo ψ2	5.37	6.8	3.5		5.67	7.9	2.5	
cis	**6.91**		**18.0**	**15.9**	**7.66**		**15.1**	**13.4**
**Ratio c/t**	**1.15**		**0.29**	**0.19**	**1.16**		**0.18**	**0.15**
**Ration t/c**	**0.87**		**4.56**	**5.29**	**0.86**		**5.62**	**6.49**

Good match between calculated and experimental populations.
Data organised by pucker show the prevalence of the *exo*-pucker (**Table 11**).

Table S7. Relative Gibbs energy, populations and dipole moments of the energetic minima of *N*-acetyl-(4*R*)-FPro-OMe **6a** identified at the SMD/MN15/aug-cc-pVTZ//MN15/cc-pVTZ level of theory in water and octanol medium.

Conformer	Octanol μ (D)	Octanol ΔG (kJ mol^{-1})	Octanol p_i (%)	Water μ (D)	Water ΔG (kJ mol^{-1})	Water p_i (%)
t-exo ψ1	5.02	0.0	55.0	5.62	0.0	60.0
t-exo ψ2	8.47	2.0	24.3	9.30	2.3	23.5
c-exo ψ1	7.31	3.5	13.6	8.06	4.1	11.7
c-exo ψ2	5.37	6.8	3.5	5.67	7.9	2.5
exo			**96.4**			**97.7**
c-endo ψ1	8.14	11.1	0.6	8.86	10.9	0.7
t-endo ψ1	2.82	8.2	2.0	3.33	10.4	0.9
c-endo ψ2	3.81	13.4	0.3	4.04	14.6	0.2
t-endo ψ2	5.74	10.8	0.7	6.40	12.1	0.4
endo			**3.6**			**2.2**

5.3.2 *N*-Acetyl-(4*R*)-FPro-NMe$_2$ **6b**

Table S8. Relative Gibbs energy, populations and dipole moments of the energetic minima of *N*-acetyl-(4*R*)-FPro-NMe$_2$ **6b** identified at the SMD/MN15/aug-cc-pVTZ//MN15/cc-pVTZ level of theory in water and octanol medium.

	**Octanol**				**Water**			
Conformer	μ D	ΔG kJ mol^{-1}	p_i %	$p_{i,exp}$ %	μ D	ΔG kJ mol^{-1}	p_i %	$p_{i,exp}$ %
t-endo ψ1	4.46	9.2	2.1		5.17	9.3	1.8	
t-exo ψ1	4.19	0.0	85.7		6.23	0.0	76.0	
t-endo ψ2	7.90	29.6	0.0		8.98	27.8	0.0	
t-exo ψ2	10.91	13.3	0.4		12.19	10.1	1.3	
trans	**4.22**		**88.2**	**89.2**	**6.30**		**79.0**	**81.6**
c-endo ψ1	11.09	11.7	0.8		12.20	9.9	1.4	
c-exo ψ1	9.87	5.3	10.1		10.84	3.4	19.4	
c-endo ψ2	3.52	30.9	0.0		3.73	29.5	0.0	
c-exo ψ2	6.25	11.2	0.9		6.73	14.2	0.2	
cis	**9.66**		**11.8**	**10.8**	**10.88**		**21.0**	**18.4**
**Ratio c/t**	**2.28**		**0.13**	**0.12**	**1.72**		**0.27**	**0.23**
**Ratio t/c**	**0.44**		**7.47**	**8.29**	**0.58**		**3.76**	**4.44**

Excellent match between calculated/experimental populations.
Data organised by pucker show the prevalence of the *exo*-pucker (**Table S9**).

Table S9. Relative Gibbs energy, populations and dipole moments of the energetic minima of *N*-acetyl-(4*R*)-FPro-NMe$_2$ **6b** identified at the SMD/MN15/aug-cc-pVTZ//MN15/cc-pVTZ level of theory in water and octanol medium.

	**Octanol**			**Water**		
Conformer	μ D	ΔG kJ mol^{-1}	p_i %	μ D	ΔG kJ mol^{-1}	p_i %
t-exo ψ1	4.19	0.0	85.7	6.23	0.0	76.0
t-exo ψ2	10.91	13.3	0.4	12.19	10.1	1.3
c-exo ψ1	9.87	5.3	10.1	10.84	3.4	19.4
c-exo ψ2	6.25	11.2	0.9	6.73	14.2	0.2
exo			**97.1**			**96.9**
c-endo ψ1	11.09	11.7	0.8	12.20	9.9	1.4
c-endo ψ2	3.52	30.9	0.0	3.73	29.5	0.0
t-endo ψ1	4.46	9.2	2.1	5.17	9.3	1.8
t-endo ψ2	7.90	29.6	0.0	8.98	27.8	0.0
endo			**2.9**			**3.2**

5.3.3 *N*-Acetyl-4,4-F_2Pro-OMe **7a**

Table S10. Relative Gibbs energy, populations and dipole moments of the energetic minima of *N*-acetyl-4,4-F_2Pro-OMe **7a** identified at the SMD/MN15/aug-cc-pVTZ//MN15/cc-pVTZ level of theory in water and octanol medium.

	**Octanol**				**Water**			
Conformer	μ D	ΔG kJ mol^{-1}	p_i %	$p_{i,exp}$ %	μ D	ΔG kJ mol^{-1}	p_i %	$p_{i,exp}$ %
t-exo ψ1	2.18	0.02	27.0		2.75	2.2	14.8	
t-exo ψ2	6.21	2.7	9.3		6.85	5.0	4.8	
t-exo			36.3				19.6	
t-endo ψ1	3.95	0.0	27.3		4.65	0.0	35.7	
t-endo ψ2	5.03	1.4	15.2		5.61	2.5	13.0	
t-endo			42.5				48.7	
trans	**3.82**		**78.9**	**67.6**	**4.57**		**68.3**	**77.7**
c-exo ψ1	7.54	6.1	2.3		8.29	4.0	7.0	
c-exo ψ2	4.65	6.7	1.8		4.89	7.7	1.6	
c-exo			4.1				8.6	
c-endo ψ1	8.03	2.3	10.6		8.84	1.8	17.2	
c-endo ψ2	3.48	3.6	6.4		3.77	4.5	5.8	
c-endo			17.0				23.0	
cis	**6.31**		**21.1**	**32.4**	**7.58**		**31.7**	**22.3**
**Δμ(c-t)**	**2.49**				**3.01**			

Trends in population do not match the experiment.
Data organised by pucker show the presence of both puckers (**Table S11**).

Table S11. Relative Gibbs energy, populations and dipole moments of the energetic minima of *N*-acetyl-4,4-F_2Pro-OMe **7a** identified at the SMD/MN15/aug-cc-pVTZ//MN15/cc-pVTZ level of theory in water and octanol medium.

	**Octanol**			**Water**		
Conformer	μ D	ΔG kJ mol^{-1}	p_i %	μ D	ΔG kJ mol^{-1}	p_i %
t-exo ψ1	2.18	0.02	27.0	2.75	2.2	14.8
c-exo ψ1	7.54	6.1	2.3	8.29	4.0	7.0
t-exo ψ2	6.21	2.7	9.3	6.85	5.0	4.8
c-exo ψ2	4.65	6.7	1.8	4.89	7.7	1.6
**exo**			**40.4**			**28.2**
t-endo ψ1	3.95	0.0	27.3	4.65	0.0	35.7
c-endo ψ1	8.03	2.3	10.6	8.84	1.8	17.2
t-endo ψ2	5.03	1.4	15.2	5.61	2.5	13.0
c-endo ψ2	3.48	3.6	6.4	3.77	4.5	5.8
endo			**59.5**			**71.7**

5.3.4 *N*-Acetyl-4,4-F_2Pro-NMe_2 **7b**

Table S12. Relative Gibbs energy, populations and dipole moments of the energetic minima of N-acetyl-4,4-F_2Pro-NMe_2 **7b** identified at the SMD/MN15/aug-cc-pVTZ//MN15/cc-pVTZ level of theory in water and octanol medium.

	**Octanol**				**Water**			
Conformer	ΔG kJ mol^{-1}	μ D	p_i %	$p_{i,exp}$ %	ΔG kJ mol^{-1}	μ D	p_i %	$p_{i,exp}$ %
t-exo ψ1	0.0	2.88	64.2		0.7	4.52	26.9	
t-exo ψ2	13.4	8.90	0.3		8.1	9.98	1.4	
t-exo								
t-endo ψ1	3.2	5.54	17.9		0.0	6.46	35.1	
t-endo ψ2	19.8	7.17	0.0		20.5	8.05	0.0	
t-endo								
trans		**3.48**	**82.5**	**82.6**		**5.71**	**63.4**	**78.6**
c-exo ψ1	7.8	10.41	2.8		2.0	11.41	15.8	
c-exo ψ2	13.9	5.09	0.2		14.9	5.48	0.1	
c-exo								
c-endo ψ1	3.7	11.15	14.5		1.3	12.31	20.6	
c-endo ψ2	19.5	2.19	0.0		20.4	2.22	0.0	
c-endo								
cis		**10.94**	**17.5**	**17.4**		**11.91**	**36.6**	**21.4**
**Δμ(c-t)**		**7.46**				**6.20**		

Excellent match between calculated/experimental populations in Oct, OK in water
Data organised by pucker show the presence of both puckers (**Table S13**).

Table S13. Relative Gibbs energy, populations and dipole moments of the energetic minima of *N*-acetyl-4,4-F_2Pro-NMe_2 **7b** identified at the SMD/MN15/aug-cc-pVTZ//MN15/cc-pVTZ level of theory in water and octanol medium.

	**Octanol**			**Water**		
Conformer	ΔG kJ mol^{-1}	μ D	p_i %	ΔG kJ mol^{-1}	μ D	p_i %
t-exo ψ1	0.0	2.88	64.2	0.7	4.52	26.9
t-exo ψ2	13.4	8.90	0.3	8.1	9.98	1.4
c-exo ψ1	7.8	10.41	2.8	2.0	11.41	15.8
c-exo ψ2	13.9	5.09	0.2	14.9	5.48	0.1
exo			**67.5**			**44.2**
t-endo ψ1	3.2	5.54	17.9	0.0	6.46	35.1
t-endo ψ2	19.8	7.17	0.0	20.5	8.05	0.0
c-endo ψ1	3.7	11.15	14.5	1.3	12.31	20.6
c-endo ψ2	19.5	2.19	0.0	20.4	2.22	0.0
endo			**32.4**			**55.7**

6 Comparison experimental and calculated ($K_{t/c}^{oct}$) / ($K_{t/c}^{wat}$) values

Table S14. Comparison experimental and calculated ($K_{t/c}^{oct}$) / ($K_{t/c}^{wat}$) values

	Experimental ($K_{t/c}^{oct}$) / ($K_{t/c}^{wat}$) values		Calculated ($K_{t/c}^{oct}$) / ($K_{t/c}^{wat}$) values	
**2**	0.75 / 0.86	0.87	0.90 / 0.91	0.99
**3**	0.72 / 0.84	0.86	1.27 / 0.99	1.28
**4**	1.43 / 0.95	1.51	1.36 / 1.08	1.26
**5**	1.17 / 1.73	1.60	1.39 / 0.98	1.42
**6a**	5.29 / 6.49	0.82	4.56 / 5.62	0.81
**6b**	8.29 / 4.44	1.92	7.47 / 3.76	1.99
**7a**	2.09 / 3.48	0.60	3.74 / 2.15	1.74
**7b**	4.76 / 3.68	1.29	4.71 / 1.73	2.72

7 Structural coordinates and total energies

7.1 *N*-Acetyl-3-fluoropyrrolidine **2**, ax-*E*-rotamer, solvent = n-octanol

SCF energy (Hartree) = -464.174680

center number	atomlabel	x	y	z
1	N	-0.38313000	0.13699400	0.15044200
2	C	0.42665700	-1.02723800	0.49564900
3	C	1.83742600	-0.53455600	0.25554400
4	C	0.41868800	1.35083400	0.00312700
5	C	1.76310400	0.93896700	0.59121900
6	C	-1.70849700	0.13584200	-0.07235900
7	O	-2.30334700	1.16580700	-0.40652600
8	C	-2.41655600	-1.17864500	0.10021900
9	H	-0.04496700	2.18288900	0.52891400
10	H	0.50404500	1.62107700	-1.05135300
11	H	-2.11902300	-1.86824900	-0.69159300
12	H	-3.48841500	-1.01275800	0.04378200
13	H	-2.16397400	-1.64260100	1.05397000
14	H	0.20229500	-1.88682800	-0.13406300
15	H	0.30150200	-1.31334300	1.54250500
16	H	1.75626600	1.04818000	1.67612900
17	H	2.60469700	1.49473500	0.18563200
18	F	2.11522100	-0.65830700	-1.11028900
19	H	2.59434200	-1.09498200	0.79738700

7.2 *N*-Acetyl-3-fluoropyrrolidine **2**, eq-*E*-rotamer, solvent = n-octanol

SCF energy (Hartree) = -464.171451

center number	atomlabel	x	y	z
1	N	0.48507300	0.14374400	-0.00623100
2	C	-0.48951700	-0.93645300	-0.02890000
3	C	-1.76678100	-0.21606900	0.37953400
4	C	-0.14142100	1.46670500	0.04031900
5	C	-1.60703300	1.15988100	-0.23299000
6	C	1.82242300	0.00477400	-0.00351600
7	O	2.56402400	0.99079200	0.05215200
8	C	2.35404400	-1.39902700	-0.06737300
9	H	0.30325800	2.12784200	-0.70054800
10	H	0.00511800	1.91802900	1.02392500
11	H	2.12697700	-1.92774700	0.85989000
12	H	3.43102900	-1.36317400	-0.20280100
13	H	1.89698900	-1.95634500	-0.88549100
14	H	-0.22213900	-1.74182900	0.65178900
15	H	-0.62276200	-1.34855500	-1.03372600
16	H	-1.78297800	1.07270100	-1.30683000
17	H	-2.28522700	1.90019300	0.18244900
18	H	-1.86085000	-0.16417300	1.46453600
19	F	-2.88082300	-0.88092800	-0.10547000

7.3 *N*-Acetyl-3-fluoropyrrolidine **2**, ax-*Z*-rotamer, solvent = n-octanol

SCF energy (Hartree) = -464.174915

center number	atomlabel	x	y	z
1	N	0.38672100	-0.00075200	-0.14967500
2	C	-0.55807700	-1.07496400	-0.43600200
3	C	-1.90094900	-0.40517100	-0.24553700
4	C	-0.26971400	1.29966000	-0.01851300
5	C	-1.64199500	1.03586700	-0.62835800
6	C	1.69744500	-0.24953000	0.01047800
7	O	2.15250600	-1.39059500	-0.12269000
8	C	2.56812300	0.92750800	0.34836100
9	H	0.27967000	2.07686900	-0.54572300
10	H	-0.35513900	1.58632800	1.03217400
11	H	2.61002500	1.61833500	-0.49526000
12	H	3.57047700	0.57314400	0.57042900
13	H	2.17211100	1.47522200	1.20398100
14	H	-0.42703900	-1.91372600	0.24603700
15	H	-0.44682000	-1.44050800	-1.45872700
16	H	-1.59406000	1.10387700	-1.71560700
17	H	-2.41635300	1.70593300	-0.26353100
18	F	-2.21963300	-0.44105100	1.11749600
19	H	-2.71227000	-0.88621300	-0.78456400

7.4 *N*-Acetyl-3-fluoropyrrolidine **2**, eq-*Z*-rotamer, solvent = n-octanol

SCF energy (Hartree) = -464.171381

center number	atomlabel	x	y	z
1	N	0.47679200	0.01526700	0.02119600
2	C	-0.59596200	-0.96791500	0.03268400
3	C	-1.81279700	-0.12027000	0.37369500
4	C	-0.02117500	1.39158900	0.08563700
5	C	-1.49690800	1.22594000	-0.24562200
6	C	1.76790800	-0.35740700	-0.02107200
7	O	2.08834100	-1.54832400	-0.08750200
8	C	2.78501900	0.74808000	0.00781300
9	H	0.49500800	2.03410400	-0.62445700
10	H	0.11654800	1.80550000	1.08768200
11	H	2.73528600	1.32812700	-0.91510800
12	H	3.77567100	0.31324000	0.10260000
13	H	2.60114600	1.43143400	0.83735600
14	H	-0.39843200	-1.75757200	0.75459600
15	H	-0.73499500	-1.42277800	-0.95193100
16	H	-1.63324900	1.14077600	-1.32534100
17	H	-2.11500800	2.03533100	0.13334300
18	H	-1.95361100	-0.04155900	1.45197500
19	F	-2.96546200	-0.67855300	-0.15532000

7.5 *N*-Acetyl-3-fluoropyrrolidine **2**, ax-*E*-rotamer, solvent = water

SCF energy (Hartree) = -464.173326

center number	atomlabel	x	y	z
1	N	-0.38606100	0.13833000	0.14556400
2	C	0.42497000	-1.02965600	0.48517200
3	C	1.83583400	-0.53450200	0.25859900
4	C	0.41996500	1.35144500	0.00212000
5	C	1.75918000	0.93737600	0.59854700
6	C	-1.70809100	0.13255800	-0.07248800
7	O	-2.31019600	1.16594500	-0.40674800
8	C	-2.41337300	-1.18031100	0.10269100
9	H	-0.04342300	2.18445600	0.52554800
10	H	0.51323000	1.62090500	-1.05143200
11	H	-2.10731300	-1.87219900	-0.68336900
12	H	-3.48549900	-1.02011300	0.04258200
13	H	-2.15890200	-1.63659700	1.05918100
14	H	0.20240300	-1.88295100	-0.15267600
15	H	0.29265300	-1.32388400	1.52765600
16	H	1.74282400	1.04135900	1.68326500
17	H	2.60292300	1.49496500	0.20119300
18	F	2.12513500	-0.65181000	-1.10756400
19	H	2.58797300	-1.09698700	0.80332600

7.6 *N*-Acetyl-3-fluoropyrrolidine **2**, eq-*E*-rotamer, solvent = water

SCF energy (Hartree) = -464.169794

center number	atomlabel	x	y	z
1	N	0.48700700	0.14641000	-0.01131600
2	C	-0.48825000	-0.93581600	-0.02489200
3	C	-1.76544100	-0.21556600	0.38146500
4	C	-0.14293700	1.46889700	0.04123200
5	C	-1.60740300	1.16036200	-0.23089000
6	C	1.81961800	0.00197900	-0.00545900
7	O	2.56867300	0.99003400	0.05113900
8	C	2.34811400	-1.40065300	-0.06647000
9	H	0.29705200	2.13370200	-0.69849400
10	H	0.00252900	1.91582800	1.02622100
11	H	2.11499800	-1.92502900	0.86139500
12	H	3.42497700	-1.37036800	-0.20103500
13	H	1.88696500	-1.95631700	-0.88282600
14	H	-0.21632900	-1.73543900	0.65961700
15	H	-0.62082100	-1.35309700	-1.02663700
16	H	-1.78248900	1.07251400	-1.30444300
17	H	-2.28430100	1.90026300	0.18605600
18	H	-1.86423400	-0.16510900	1.46531600
19	F	-2.87766600	-0.88192400	-0.10944300

7.7 *N*-Acetyl-3-fluoropyrrolidine **2**, ax-*Z*-rotamer, solvent = water

SCF energy (Hartree) = -464.173423

center number	atomlabel	x	y	z
1	N	0.38857700	-0.00289600	-0.15621900
2	C	-0.56097800	-1.07515600	-0.44187800
3	C	-1.90132500	-0.40382200	-0.24481700
4	C	-0.26758600	1.29972500	-0.02157600
5	C	-1.64272400	1.03769900	-0.62342700
6	C	1.69529800	-0.24668900	0.00975600
7	O	2.15664100	-1.39246200	-0.11992500
8	C	2.56229800	0.92936100	0.35020600
9	H	0.28100400	2.07488300	-0.55117600
10	H	-0.34390000	1.58529200	1.02928800
11	H	2.59825800	1.62184700	-0.49178900
12	H	3.56588200	0.57991300	0.57257300
13	H	2.16041300	1.47267200	1.20543700
14	H	-0.43002400	-1.91593700	0.23702900
15	H	-0.45706300	-1.43665500	-1.46591300
16	H	-1.60177000	1.10851000	-1.71020600
17	H	-2.41357500	1.70698100	-0.25114400
18	F	-2.21420600	-0.44460300	1.12119000
19	H	-2.71444000	-0.88281400	-0.78146500

7.8 *N*-Acetyl-3-fluoropyrrolidine **2**, eq-*Z*-rotamer, solvent = water

SCF energy (Hartree) = -464.169850

center number	atomlabel	x	y	z
1	N	0.47803200	0.01263900	0.01091800
2	C	-0.59752400	-0.96881600	0.02749500
3	C	-1.81037500	-0.11986200	0.37675100
4	C	-0.02000600	1.39116600	0.07850800
5	C	-1.49729000	1.22650000	-0.24236100
6	C	1.76675500	-0.35433600	-0.02199400
7	O	2.09241800	-1.55044700	-0.08646500
8	C	2.78106900	0.75030300	0.01485400
9	H	0.49274800	2.03137800	-0.63514500
10	H	0.12630800	1.80304100	1.07896200
11	H	2.72666100	1.33737200	-0.90287000
12	H	3.77322700	0.31947900	0.10760800
13	H	2.59066800	1.42590700	0.84877600
14	H	-0.39855200	-1.75857000	0.74815400
15	H	-0.74450000	-1.42116300	-0.95646400
16	H	-1.64117000	1.14322300	-1.32091900
17	H	-2.11021200	2.03557700	0.14405300
18	H	-1.94678800	-0.04406700	1.45496500
19	F	-2.96552600	-0.67853500	-0.15015000

7.9 *N*-Acetyl-3,3-difluoropyrrolidine **3**, *E*-rotamer, solvent = n-octanol

SCF energy (Hartree) = -563.3898509

center number	atomlabel	x	y	z
1	N	-0.69009300	0.15783400	-0.14273300
2	C	0.27918500	-0.91594500	-0.28559700
3	C	1.57935200	-0.19144700	0.00661900
4	C	-0.07101000	1.48501900	-0.07915400
5	C	1.37013600	1.21580200	-0.49297900
6	C	-2.01994400	0.00180300	0.00521800
7	O	-2.75568400	0.97655900	0.17572200
8	C	-2.54031200	-1.40614900	-0.04399800
9	H	0.11989300	-1.72599600	0.42346800
10	H	0.31253100	-1.31862700	-1.29978400
11	F	2.65212800	-0.81642800	-0.53593000
12	F	1.79411700	-0.17288700	1.35845200
13	H	1.46919700	1.20387300	-1.57773500
14	H	2.09048300	1.90707200	-0.06469800
15	H	-0.13215300	1.87886200	0.93682500
16	H	-0.58107900	2.17856100	-0.74342000
17	H	-2.16488000	-1.93621100	-0.91968600
18	H	-3.62578000	-1.38112400	-0.06607400
19	H	-2.21272600	-1.95438600	0.84111600

7.10 *N*-Acetyl-3,3-difluoropyrrolidine **3**, *Z*-rotamer, solvent = n-octanol

SCF energy (Hartree) = -563.390063

center number	atomlabel	x	y	z
1	N	0.68813200	0.01871200	-0.10234900
2	C	-0.37074700	-0.97366700	-0.18930000
3	C	-1.61157800	-0.12402200	0.00126200
4	C	0.18215600	1.39394300	-0.05819500
5	C	-1.25356300	1.23730300	-0.54146000
6	C	1.97876100	-0.35516500	-0.01322400
7	O	2.29764400	-1.54621100	-0.04928300
8	C	2.98655400	0.74955100	0.12130800
9	H	0.76544700	2.05016800	-0.69968100
10	H	0.21227500	1.78442300	0.96121500
11	H	3.01216600	1.34850800	-0.79034000
12	H	3.96663200	0.31400800	0.29135000
13	H	2.72980900	1.41447900	0.94649500
14	H	-0.28448900	-1.73401800	0.58505000
15	H	-0.39818500	-1.45904200	-1.16592600
16	H	-1.29182100	1.18601900	-1.62892800
17	H	-1.92960800	2.00767700	-0.18132500
18	F	-1.89549800	-0.02249000	1.33739500
19	F	-2.71003500	-0.67097400	-0.57401200

7.11 *N*-Acetyl-3,3-difluoropyrrolidine **3**, *E*-rotamer, solvent = water

SCF energy (Hartree) = - 563.3880889

center number	atomlabel	x	y	z
1	N	-0.69308300	0.15943000	-0.15193300
2	C	0.27797500	-0.91727000	-0.28004300
3	C	1.57733100	-0.19104000	0.00710800
4	C	-0.07003100	1.48587300	-0.08366000
5	C	1.36965600	1.21407600	-0.49722500
6	C	-2.01756700	-0.00086300	0.00423400
7	O	-2.75720300	0.97681100	0.18413700
8	C	-2.53690400	-1.40658500	-0.04592500
9	H	0.11415100	-1.71846600	0.43719100
10	H	0.31220000	-1.33054700	-1.28879000
11	F	2.64973600	-0.81906000	-0.53335200
12	F	1.79613100	-0.16731500	1.35927800
13	H	1.46647900	1.19696100	-1.58162800
14	H	2.08968000	1.90646400	-0.07131600
15	H	-0.12986700	1.87668600	0.93303800
16	H	-0.57562500	2.18247300	-0.74722700
17	H	-2.15980700	-1.93218200	-0.92301400
18	H	-3.62211700	-1.38555600	-0.06437200
19	H	-2.20144300	-1.95410800	0.83627600

7.12 *N*-Acetyl-3,3-difluoropyrrolidine **3**, *Z*-rotamer, solvent = water

SCF energy (Hartree) = -563. 3883054

center number	atomlabel	x	y	z
1	N	0.68980300	0.01707700	-0.11111300
2	C	-0.37238500	-0.97336600	-0.19668600
3	C	-1.61048000	-0.12269000	0.00185600
4	C	0.18344600	1.39433000	-0.06137200
5	C	-1.25442700	1.23991400	-0.53631500
6	C	1.97720700	-0.35230400	-0.01470500
7	O	2.29971000	-1.54858700	-0.04859400
8	C	2.98293100	0.75040800	0.12594400
9	H	0.76381200	2.05035600	-0.70439900
10	H	0.22194000	1.78100100	0.95827500
11	H	3.00064100	1.35793400	-0.77966400
12	H	3.96481200	0.31766400	0.29002000
13	H	2.72305000	1.40643300	0.95691100
14	H	-0.28553500	-1.73588700	0.57483300
15	H	-0.40714100	-1.45392800	-1.17470400
16	H	-1.29922600	1.19257200	-1.62326700
17	H	-1.92638800	2.00930400	-0.16787900
18	F	-1.89077600	-0.02848100	1.34000400
19	F	-2.71144700	-0.66863600	-0.57066100

7.13 *N*-Acetyl-3-fluoropiperidine **4**, ax_*E*-rotamer, solvent = n-octanol

SCF energy (Hartree) = -503.4415043

center number	atomlabel	x	y	z
1	N	-0.49889400	0.34848500	0.65014200
2	C	0.29397300	1.56462900	0.51588400
3	C	1.38532900	1.34231300	-0.52055900
4	C	2.25044500	0.15353000	-0.11451300
5	C	1.40557900	-1.07310400	0.15588500
6	C	0.27599100	-0.78193800	1.12604300
7	F	0.81985700	-1.48288100	-1.05098000
8	H	2.00992200	-1.90461800	0.51744100
9	C	-1.66202900	0.22843200	-0.03800700
10	C	-2.36716600	-1.10175500	-0.00345300
11	O	-2.14746400	1.18040600	-0.65361500
12	H	2.99097400	-0.08223300	-0.87912100
13	H	0.91424300	1.15236300	-1.48802500
14	H	1.99692300	2.23898700	-0.62131900
15	H	0.73970100	1.78243800	1.49065400
16	H	-0.36463000	2.38279100	0.24298100
17	H	-0.34924900	-1.66123300	1.24676400
18	H	0.70799300	-0.53681900	2.09948400
19	H	2.79155600	0.38720700	0.80687700
20	H	-2.51965100	-1.44742700	1.01872800
21	H	-3.32965500	-0.98298600	-0.49226900
22	H	-1.78760600	-1.85782500	-0.53314000

7.14 *N*-Acetyl-3-fluoropiperidine **4**, ax_*Z*-rotamer, solvent = n-octanol

SCF energy (Hartree) = -503.4407333

center number	atomlabel	x	y	z
1	N	0.52234300	0.15568800	-0.65543200
2	C	-0.04442300	1.49405100	-0.55972200
3	C	-1.16045200	1.50617000	0.47465600
4	C	-2.21723600	0.47007600	0.10733800
5	C	-1.59392500	-0.89308200	-0.10658600
6	C	-0.43928800	-0.84217900	-1.08763900
7	F	-1.08210300	-1.33906700	1.11960400
8	H	-2.33350000	-1.62444400	-0.43154700
9	C	1.67425800	-0.23235300	-0.05489000
10	C	2.52980100	0.84124800	0.56371300
11	O	2.02758000	-1.41313000	-0.02342300
12	H	-2.98793500	0.39745500	0.87501800
13	H	-0.73583300	1.27558200	1.45508800
14	H	-1.60636400	2.49927400	0.53181300
15	H	-0.44984000	1.75382900	-1.54246800
16	H	0.73070600	2.21641600	-0.32482900
17	H	0.05062400	-1.80892400	-1.14892800
18	H	-0.82603500	-0.57331600	-2.07365800
19	H	-2.70840300	0.75754800	-0.82657800
20	H	1.99235800	1.35394200	1.36250600
21	H	3.41700000	0.36897700	0.97489900
22	H	2.82669200	1.58690200	-0.17356300

7.15 *N*-Acetyl-3-fluoropiperidine **4**, eq_*E*-rotamer, solvent = n-octanol

SCF energy (Hartree) = -503.4395306

center number	atomlabel	x	y	z
1	N	-0.66831100	0.14512900	0.54271000
2	C	-0.21543000	1.52749400	0.63928400
3	C	0.83246500	1.79346600	-0.43199100
4	C	1.98109800	0.79726500	-0.30181800
5	C	1.44616200	-0.61544000	-0.32843600
6	C	0.39625100	-0.81828300	0.75025100
7	H	1.02014100	-0.85622200	-1.30640400
8	F	2.48793600	-1.51144100	-0.09266600
9	C	-1.85263000	-0.14624800	-0.05094100
10	C	-2.18356100	-1.59792600	-0.27386100
11	O	-2.64268600	0.73815600	-0.38770500
12	H	2.71491000	0.93096800	-1.09675600
13	H	0.36063000	1.69770300	-1.41373000
14	H	1.20622500	2.81349700	-0.34444100
15	H	0.21906900	1.67165500	1.63230600
16	H	-1.07423700	2.18250300	0.53552700
17	H	0.02080400	-1.83659900	0.73626100
18	H	0.85690500	-0.63578800	1.72552500
19	H	2.48822300	0.94273000	0.65734300
20	H	-2.15009200	-2.16114200	0.65840500
21	H	-3.18317000	-1.65406400	-0.69430300
22	H	-1.47729700	-2.05537700	-0.96799000

7.16 *N*-Acetyl-3-fluoropiperidine **4**, eq_*Z*-rotamer, solvent = n-octanol

SCF energy (Hartree) = -503.4396041

center number	atomlabel	x	y	z
1	N	-0.64940400	-0.01744000	0.58287700
2	C	-0.37443100	1.41028100	0.68385000
3	C	0.60573800	1.81732400	-0.40769600
4	C	1.87173800	0.97010100	-0.31693400
5	C	1.51846000	-0.49957700	-0.33882700
6	C	0.53539300	-0.83687900	0.76728200
7	H	1.09471100	-0.78986800	-1.30380400
8	F	2.67488700	-1.25391500	-0.14445400
9	C	-1.73447000	-0.54228300	-0.04085600
10	C	-2.86958700	0.39072900	-0.36675300
11	O	-1.80297200	-1.74086400	-0.32097900
12	H	2.55943800	1.19906900	-1.13114700
13	H	0.12705000	1.67298800	-1.38087400
14	H	0.85172300	2.87505700	-0.31418700
15	H	0.06768100	1.59600800	1.66658400
16	H	-1.29750500	1.97796800	0.62581400
17	H	0.26314700	-1.88730200	0.74387100
18	H	0.98831100	-0.59863900	1.73324500
19	H	2.38394000	1.17536200	0.62846600
20	H	-2.55617600	1.14281200	-1.09218600
21	H	-3.67783200	-0.19754800	-0.79076800
22	H	-3.22590600	0.91014000	0.52238100

7.17 *N*-Acetyl-3-fluoropiperidine **4**, ax_*E*-rotamer, solvent = water

SCF energy (Hartree) = -503.4395687

center number	atomlabel	x	y	z
1	N	-0.49051600	0.37088100	0.70954800
2	C	0.34061400	1.56230800	0.55115000
3	C	1.37861800	1.31482500	-0.53163600
4	C	2.22634800	0.10272600	-0.16036900
5	C	1.35750100	-1.09945700	0.13741400
6	C	0.27531800	-0.78423300	1.15185200
7	F	0.71368700	-1.48093100	-1.05202000
8	H	1.94820200	-1.95172100	0.46940000
9	C	-1.61773700	0.25195400	-0.03417300
10	C	-2.36018100	-1.05546600	0.00636400
11	O	-2.05413000	1.20001500	-0.70073100
12	H	2.93166700	-0.15113100	-0.95141100
13	H	0.86275000	1.13938800	-1.47891300
14	H	2.00863300	2.19497700	-0.65563600
15	H	0.83505900	1.74707600	1.50750800
16	H	-0.29483800	2.41085500	0.32074100
17	H	-0.36837900	-1.64595800	1.29681500
18	H	0.75184900	-0.55175500	2.10566400
19	H	2.80370800	0.31933900	0.74219200
20	H	-2.52447500	-1.38913700	1.02994300
21	H	-3.31611500	-0.91565800	-0.48924300
22	H	-1.79747400	-1.83011000	-0.51348400

7.18 *N*-Acetyl-3-fluoropiperidine **4**, ax_*Z*-rotamer, solvent = water

SCF energy (Hartree) = -503.4390919

center number	atomlabel	x	y	z
1	N	0.52086900	0.16565200	-0.72363600
2	C	-0.04924000	1.50365400	-0.58742700
3	C	-1.10702800	1.49972000	0.50468200
4	C	-2.18340400	0.47207900	0.17315200
5	C	-1.57557600	-0.88768400	-0.09375600
6	C	-0.46745900	-0.82381000	-1.12528300
7	F	-1.01005300	-1.35766800	1.10277000
8	H	-2.33019200	-1.61167100	-0.39740300
9	C	1.63543000	-0.23166100	-0.06497500
10	C	2.47949500	0.83258000	0.57950300
11	O	1.97352500	-1.42163100	-0.00678500
12	H	-2.91660400	0.38736100	0.97498600
13	H	-0.62932400	1.25204700	1.45629500
14	H	-1.54645500	2.49195600	0.60189900
15	H	-0.50833900	1.76228700	-1.54484500
16	H	0.73398000	2.22850000	-0.39225700
17	H	0.00840100	-1.79276100	-1.23448100
18	H	-0.89984200	-0.53106500	-2.08346300
19	H	-2.71548500	0.77549300	-0.73216500
20	H	1.91694600	1.34525100	1.36074700
21	H	3.35162800	0.35664600	1.01690700
22	H	2.79818000	1.57919300	-0.14679200

7.19 *N*-Acetyl-3-fluoropiperidine **4**, eq_*E*-rotamer, solvent = water

SCF energy (Hartree) = -503.4372993

center number	atomlabel	x	y	z
1	N	-0.67890100	0.14290000	0.66795700
2	C	-0.24604700	1.53905000	0.69484600
3	C	0.70707900	1.78959100	-0.46401100
4	C	1.88823800	0.82659100	-0.38439600
5	C	1.39050000	-0.59812600	-0.31718000
6	C	0.43045700	-0.78257300	0.84362200
7	H	0.89720400	-0.88762900	-1.24919700
8	F	2.47186300	-1.45952700	-0.12818800
9	C	-1.78362000	-0.18718800	-0.04843100
10	C	-2.04686400	-1.64538800	-0.29799300
11	O	-2.56342500	0.67675000	-0.46833600
12	H	2.55479500	0.94620300	-1.23810300
13	H	0.16283000	1.64044600	-1.40072900
14	H	1.05830500	2.82056500	-0.44518800
15	H	0.26348700	1.71011700	1.64491500
16	H	-1.11859300	2.18171300	0.64819000
17	H	0.08504100	-1.80967900	0.90487300
18	H	0.94705400	-0.53669100	1.77370900
19	H	2.45991300	1.02240900	0.52789200
20	H	-2.04935400	-2.21427900	0.63072600
21	H	-3.01175700	-1.73934000	-0.78640100
22	H	-1.27444400	-2.06413200	-0.94474700

7.20 *N*-Acetyl-3-fluoropiperidine **4**, eq_*Z*-rotamer, solvent = water

SCF energy (Hartree) = -503.4374572

center number	atomlabel	x	y	z
1	N	-0.65300200	-0.03088900	0.67973300
2	C	-0.40915700	1.40986600	0.72557900
3	C	0.49964900	1.80600900	-0.42817000
4	C	1.79376000	0.99885500	-0.37472100
5	C	1.48046300	-0.47867400	-0.33229000
6	C	0.57013300	-0.80424000	0.83670300
7	H	1.01216600	-0.80971100	-1.26270500
8	F	2.66597400	-1.19923400	-0.17924000
9	C	-1.67308300	-0.57114500	-0.03392500
10	C	-2.81188100	0.33071700	-0.41655500
11	O	-1.68149900	-1.76882000	-0.34508500
12	H	2.43123000	1.21886500	-1.23069100
13	H	-0.02503200	1.61088700	-1.36821900
14	H	0.71710400	2.87255700	-0.38372000
15	H	0.07861000	1.63056200	1.67707400
16	H	-1.34980900	1.95017200	0.70279800
17	H	0.34031600	-1.86408900	0.87257000
18	H	1.05879400	-0.50656200	1.76630100
19	H	2.34440700	1.24910500	0.53714600
20	H	-2.47165000	1.09001000	-1.12207000
21	H	-3.58319800	-0.27287800	-0.88473200
22	H	-3.22299500	0.84263600	0.45222200

7.21 *N*-Acetyl-3,3-difluoropiperidine **5**, *E*-rotamer, solvent = n-octanol

SCF energy (Hartree) = - 602.6725384

center number	atomlabel	x	y	z
1	C	0.41191000	1.66247700	-0.60626000
2	C	-0.64290300	1.86910400	0.47126800
3	C	-1.84746600	0.97114700	0.20377200
4	C	-1.40003100	-0.45597000	0.02982100
5	C	-0.29765900	-0.62074400	-0.99952000
6	H	-2.57759500	1.00819200	1.01217500
7	H	-0.20518800	1.62979100	1.44301900
8	H	-0.95590900	2.91212400	0.49585800
9	H	0.00743300	1.92846500	-1.58654600
10	H	1.29825800	2.25973100	-0.42000300
11	H	0.00578000	-1.66300200	-1.03216900
12	H	-0.69836800	-0.33971100	-1.97538500
13	H	-2.34152100	1.26805800	-0.72378300
14	N	0.79750600	0.25618100	-0.64792200
15	C	1.92080000	-0.15863400	-0.00260200
16	O	2.70593300	0.64733000	0.49645900
17	C	2.19059000	-1.63796200	0.05534600
18	H	2.15668700	-2.08811600	-0.93638000
19	H	3.17708900	-1.78213400	0.48553600
20	H	1.45166000	-2.14051700	0.67977800
21	F	-0.94270100	-0.94164400	1.22950000
22	F	-2.45282900	-1.25183400	-0.31719600

7.22 *N*-Acetyl-3,3-difluoropiperidine **5**, *Z*-rotamer, solvent = n-octanol

SCF energy (Hartree) = -602.6722209

center number	atomlabel	x	y	z
1	C	0.55685500	1.53490000	-0.62381000
2	C	-0.47646500	1.87975900	0.43972600
3	C	-1.76855500	1.11039200	0.18186000
4	C	-1.47533900	-0.36068700	0.04393800
5	C	-0.39899400	-0.66710500	-0.97747100
6	H	-2.49463800	1.24510700	0.98334500
7	H	-0.07655400	1.61802100	1.42232600
8	H	-0.67476200	2.95089600	0.43532200
9	H	0.18448000	1.82589000	-1.61050000
10	H	1.49067600	2.06146500	-0.45749700
11	H	-0.17617500	-1.72992500	-0.96533300
12	H	-0.76603400	-0.37511300	-1.96307300
13	H	-2.22183900	1.43815400	-0.75593500
14	N	0.78472700	0.09527400	-0.64128900
15	C	1.84362900	-0.52878800	-0.06026000
16	O	1.89052800	-1.75381600	0.04951300
17	C	2.97318000	0.33581100	0.42959900
18	H	2.63204600	1.01119900	1.21515600
19	H	3.74600800	-0.31466500	0.82776400
20	H	3.38846400	0.93966000	-0.37691900
21	F	-1.07590000	-0.85983700	1.25724300
22	F	-2.61019400	-1.04380500	-0.28649100

7.23 *N*-Acetyl-3,3-difluoropiperidine **5**, *E*-rotamer, solvent = water

SCF energy (Hartree) = - 602.6701879

center number	atomlabel	x	y	z
1	C	0.41216800	1.67221000	-0.63542000
2	C	-0.59174500	1.86037700	0.49129500
3	C	-1.81151600	0.97545700	0.25211100
4	C	-1.37697000	-0.44850900	0.03363800
5	C	-0.31842400	-0.60013000	-1.04160700
6	H	-2.51008900	0.99957900	1.08786400
7	H	-0.11489600	1.59548100	1.43777900
8	H	-0.89634600	2.90378700	0.55195600
9	H	-0.03984800	1.94570100	-1.59098100
10	H	1.30200800	2.27381200	-0.48623000
11	H	-0.02249600	-1.64243600	-1.11034800
12	H	-0.75419100	-0.29317600	-1.99262400
13	H	-2.33488800	1.29318800	-0.65153800
14	N	0.79936700	0.26452500	-0.71696100
15	C	1.87418900	-0.17004900	-0.00673600
16	O	2.63752800	0.62648200	0.54968700
17	C	2.13498400	-1.64927400	0.04664000
18	H	2.10864200	-2.09430000	-0.94688500
19	H	3.11371800	-1.80333400	0.49036200
20	H	1.38316400	-2.14756100	0.65815900
21	F	-0.87354500	-0.95564600	1.20788100
22	F	-2.44497600	-1.23710600	-0.28186000

7.24 *N*-Acetyl-3,3-difluoropiperidine **5**, *Z*-rotamer, solvent = water

SCF energy (Hartree) = - 602.6703583

center number	atomlabel	x	y	z
1	C	0.57851600	1.53987300	-0.65298700
2	C	-0.38778000	1.86250600	0.47631500
3	C	-1.70801400	1.13031900	0.25584800
4	C	-1.45392400	-0.33868400	0.04799100
5	C	-0.43614700	-0.62795300	-1.03590300
6	H	-2.39051900	1.24807700	1.09684400
7	H	0.05926800	1.55282400	1.42423400
8	H	-0.56227100	2.93611800	0.52364900
9	H	0.15664800	1.86236400	-1.60752300
10	H	1.52926500	2.04549000	-0.52469800
11	H	-0.24579600	-1.69554000	-1.08271100
12	H	-0.84078800	-0.28216000	-1.98700000
13	H	-2.19676800	1.50234300	-0.64641000
14	N	0.78355600	0.09432800	-0.72858500
15	C	1.78608000	-0.54825900	-0.07436300
16	O	1.79067500	-1.77673900	0.05908200
17	C	2.91118500	0.28943000	0.46241100
18	H	2.54344600	0.97179700	1.22971800
19	H	3.65289400	-0.37417000	0.89565000
20	H	3.36982800	0.88694600	-0.32429700
21	F	-1.00794800	-0.89343200	1.22270800
22	F	-2.61705200	-0.98922000	-0.25002900

7.25 *N*-Acetyl-(4*R*)-FPro-OMe **6a**, *trans*-rotamer, *exo*-pucker, psi1-rotamer, solvent=octanol

SCF energy (Hartree) = -691.906471

center number	atomlabel	x	y	z
1	C	-3.780310	-0.365840	-0.133254
2	C	-1.476024	-0.282939	0.227852
3	O	-1.658348	0.139003	1.348036
4	C	-0.107372	-0.590508	-0.342478
5	N	0.841756	0.445399	0.028087
6	C	0.489212	-1.847319	0.303169
7	C	1.972437	-1.548646	0.319051
8	C	2.035831	-0.080856	0.678486
9	C	0.566571	1.735454	-0.247752
10	O	-0.485115	2.038061	-0.817731
11	C	1.591145	2.748206	0.168621
12	H	-3.917476	0.688276	0.099574
13	H	-4.446248	-0.664917	-0.935788
14	H	-3.966505	-0.963852	0.756749
15	H	-0.187059	-0.680133	-1.425807
16	H	0.142822	-1.932112	1.334267
17	H	0.251564	-2.760528	-0.234773
18	H	2.555377	-2.188195	0.975750
19	F	2.465751	-1.702711	-0.978444
20	H	2.944275	0.380135	0.296024
21	H	1.996629	0.044942	1.762575
22	H	1.201114	3.744713	-0.015346
23	H	1.836253	2.636768	1.225326
24	H	2.510592	2.607970	-0.401883
25	O	-2.450829	-0.591035	-0.613986

7.26 *N*-Acetyl-(4*R*)-FPro-OMe **6a**, *trans*-rotamer, *exo*-pucker, psi2-rotamer, solvent=octanol

SCF energy (Hartree) = -691.905796

center number	atomlabel	x	y	z
1	C	-3.229766	-0.270058	1.026199
2	C	-1.429215	-0.724437	-0.389011
3	O	-2.184498	-1.209288	-1.200852
4	C	0.071853	-0.677669	-0.567023
5	N	0.672610	0.522749	-0.008922
6	C	0.761518	-1.802303	0.217295
7	C	2.107900	-1.200290	0.555684
8	C	1.781147	0.237119	0.894504
9	C	0.194069	1.737501	-0.343285
10	O	-0.759769	1.839313	-1.118993
11	C	0.877262	2.922764	0.271263
12	H	-3.603063	-1.292135	1.005293
13	H	-3.377065	0.165408	2.009049

14	H	-3.741945	0.321676	0.269786
15	H	0.264034	-0.750996	-1.637527
16	H	0.224421	-1.995312	1.147038
17	H	0.845528	-2.726147	-0.347463
18	H	2.653599	-1.722721	1.336305
19	F	2.894742	-1.209202	-0.597780
20	H	2.633194	0.889013	0.712564
21	H	1.482575	0.319600	1.941977
22	H	0.333651	3.824112	0.004651
23	H	0.920708	2.824450	1.356465
24	H	1.902150	2.995888	-0.095749
25	O	-1.819150	-0.250652	0.788137

7.27 *N*-Acetyl-(4R)-FPro-OMe **6a**, *trans*-rotamer, *endo*-pucker, psi1-rotamer, solvent=octanol

SCF energy (Hartree) = -691.903378

center number	atomlabel	x	y	z
1	C	-3.707297	-0.456917	0.317656
2	C	-1.393914	-0.188143	0.128962
3	O	-1.310117	0.006104	1.321213
4	C	-0.205824	-0.256020	-0.813656
5	N	0.875377	0.576132	-0.317694
6	C	0.405644	-1.659189	-0.810152
7	C	1.398185	-1.583481	0.335812
8	C	1.998601	-0.190465	0.201386
9	C	0.680613	1.907423	-0.199787
10	O	-0.383086	2.411510	-0.565721
11	C	1.802389	2.707793	0.390576
12	H	-3.847917	0.509198	0.797947
13	H	-4.535888	-0.671564	-0.348850
14	H	-3.619284	-1.232831	1.075663
15	H	-0.535253	0.054905	-1.802515
16	H	-0.334849	-2.447417	-0.697850
17	H	0.959957	-1.815935	-1.735905
18	H	0.911631	-1.711807	1.301941
19	F	2.377652	-2.554180	0.224318
20	H	2.830350	-0.227917	-0.507349
21	H	2.352783	0.207264	1.149183
22	H	2.756365	2.451638	-0.069883
23	H	1.594689	3.764702	0.252510
24	H	1.879696	2.495117	1.458374
25	O	-2.532944	-0.430948	-0.502124

7.28 *N*-Acetyl-(4*R*)-FPro-OMe **6a**, *trans*-rotamer, *endo*-pucker, psi2-rotamer, solvent=octanol

SCF energy (Hartree) = -691.902398

center number	atomlabel	x	y	z
1	C	-2.548658	-1.154122	1.502562

2	C	-1.280605	-0.859254	-0.437406
3	O	-2.125706	-1.437993	-1.081875
4	C	0.013628	-0.345669	-1.040950
5	N	0.530369	0.819646	-0.341447
6	C	1.143066	-1.370087	-0.895327
7	C	1.815161	-0.956382	0.401384
8	C	1.783290	0.564963	0.353251
9	C	-0.243177	1.921604	-0.241994
10	O	-1.354056	1.943886	-0.776262
11	C	0.307027	3.074517	0.542545
12	H	-2.639008	-2.230022	1.366586
13	H	-2.454490	-0.912401	2.556024
14	H	-3.417955	-0.657547	1.076150
15	H	-0.209527	-0.122975	-2.082246
16	H	0.788471	-2.397724	-0.897881
17	H	1.858990	-1.237338	-1.706858
18	H	1.285117	-1.335616	1.274031
19	F	3.117502	-1.418404	0.466969
20	H	2.646513	0.920731	-0.215757
21	H	1.794477	1.017900	1.341448
22	H	1.337251	3.291272	0.261432
23	H	-0.316273	3.947295	0.372046
24	H	0.295423	2.830812	1.606524
25	O	-1.354925	-0.673354	0.874577

7.29 *N*-Acetyl-(4*R*)-FPro-OMe **6a**, *cis*-rotamer, *exo*-pucker, psi1-rotamer, solvent=octanol

SCF energy (Hartree) = -691.904304

center number	atomlabel	x	y	z
1	C	-3.760870	-0.535470	-0.099312
2	C	-1.464622	-0.252417	0.231660
3	O	-1.670207	0.231271	1.321398
4	C	-0.087743	-0.508944	-0.342633
5	N	0.888471	0.474980	0.095540
6	C	0.464225	-1.824036	0.233590
7	C	1.953820	-1.571111	0.280522
8	C	2.054041	-0.132715	0.729355
9	C	0.814011	1.809763	-0.102574
10	O	1.696809	2.558396	0.318273
11	C	-0.383735	2.337003	-0.841133
12	H	-4.405182	-0.926699	-0.879326
13	H	-3.884916	-1.106522	0.818491
14	H	-3.986243	0.512341	0.089010
15	H	-0.155483	-0.556685	-1.429724
16	H	0.105126	-1.961003	1.254564
17	H	0.198932	-2.691955	-0.363114
18	H	2.508439	-2.269044	0.901239
19	F	2.453848	-1.661865	-1.020827
20	H	2.971940	0.340014	0.387238
21	H	2.004372	-0.062535	1.817599
22	H	-1.198102	2.501880	-0.133696

23	H	-0.117997	3.292915	-1.284455
24	H	-0.735201	1.659304	-1.618746
25	O	-2.419649	-0.668731	-0.586830

7.30 *N*-Acetyl-(4*R*)-FPro-OMe **6a**, *cis*-rotamer, *exo*-pucker, psi2-rotamer, solvent=octanol

SCF energy (Hartree) = -691.904187

center number	atomlabel	x	y	z
1	C	3.191967	-0.516791	-1.062921
2	C	1.385010	-0.741336	0.401391
3	O	2.121467	-1.180060	1.255822
4	C	-0.108969	-0.589401	0.588196
5	N	-0.679178	0.571849	-0.079064
6	C	-0.839086	-1.764735	-0.083661
7	C	-2.165832	-1.156232	-0.477266
8	C	-1.793113	0.226698	-0.955888
9	C	-0.294946	1.855371	0.096132
10	O	-0.833914	2.765783	-0.534336
11	C	0.815375	2.114651	1.074948
12	H	3.349560	-0.166996	-2.077561
13	H	3.754830	0.094264	-0.359192
14	H	3.496944	-1.556922	-0.967420
15	H	-0.298874	-0.571596	1.661752
16	H	-0.311798	-2.062150	-0.991073
17	H	-0.943990	-2.624805	0.571147
18	H	-2.730348	-1.738030	-1.200328
19	F	-2.946069	-1.027214	0.674227
20	H	-2.611024	0.934525	-0.841243
21	H	-1.489241	0.204541	-2.004370
22	H	1.778641	1.934998	0.592029
23	H	0.774240	3.158784	1.372327
24	H	0.752258	1.479078	1.957515
25	O	1.788101	-0.387354	-0.810705

7.31 *N*-Acetyl-(4*R*)-FPro-OMe **6a**, *cis*-rotamer, *endo*-pucker, psi1-rotamer, solvent=octanol

SCF energy (Hartree) = -691.901827

center number	atomlabel	x	y	z
1	C	3.727009	-0.144671	-0.303594
2	C	1.403020	0.074185	-0.151153
3	O	1.339505	0.361293	-1.324673
4	C	0.202991	-0.064678	0.773357
5	N	-1.002397	0.464249	0.166739
6	C	-0.142969	-1.543506	0.962808
7	C	-0.991141	-1.827687	-0.263813
8	C	-1.835887	-0.571360	-0.430151
9	C	-1.316742	1.772079	0.033848
10	O	-2.353121	2.111750	-0.537116
11	C	-0.361362	2.766242	0.629798

12	H	3.667908	-0.870181	-1.112398
13	H	3.863440	0.852673	-0.716534
14	H	4.543100	-0.391234	0.367061
15	H	0.441885	0.418157	1.719110
16	H	0.733005	-2.180259	1.051759
17	H	-0.767376	-1.654270	1.849615
18	H	-0.375842	-2.013735	-1.143865
19	F	-1.793380	-2.939837	-0.076164
20	H	-2.775149	-0.687418	0.115561
21	H	-2.061961	-0.338898	-1.468637
22	H	-0.730951	3.767287	0.428901
23	H	-0.281643	2.620935	1.707856
24	H	0.636103	2.659399	0.201379
25	O	2.533012	-0.190206	0.488277

7.32 *N*-Acetyl-(4*R*)-FPro-OMe **6a**, *cis*-rotamer, *endo*-pucker, psi2-rotamer, solvent=octanol

SCF energy (Hartree) = -691.901207

center number	atomlabel	x	y	z
1	C	-2.412535	-1.523240	1.453163
2	C	-1.150882	-0.983831	-0.439329
3	O	-1.805731	-1.753216	-1.106180
4	C	0.013771	-0.191433	-1.013351
5	N	0.448369	0.910096	-0.176024
6	C	1.263312	-1.072804	-1.073897
7	C	1.854990	-0.889395	0.312156
8	C	1.619109	0.583066	0.627852
9	C	-0.220150	2.071456	0.000824
10	O	0.196555	2.923324	0.785437
11	C	-1.470527	2.260519	-0.810174
12	H	-3.355205	-1.279000	0.967708
13	H	-2.219433	-2.590123	1.362023
14	H	-2.436704	-1.229276	2.496863
15	H	-0.291961	0.138052	-2.004293
16	H	1.043879	-2.105882	-1.330019
17	H	1.957690	-0.655029	-1.803229
18	H	1.375732	-1.538110	1.044704
19	F	3.207769	-1.183262	0.324361
20	H	2.488762	1.166685	0.318201
21	H	1.430622	0.771351	1.683036
22	H	-1.896000	3.231472	-0.575258
23	H	-1.249570	2.209374	-1.876834
24	H	-2.204495	1.484654	-0.584356
25	O	-1.345116	-0.776547	0.854607

7.33 *N*-acetyl-(4*R*)-FPro-OMe **6a**, *trans*-rotamer, *exo*-pucker, psi1-rotamer, solvent = water

SCF energy (Hartree) = -691.905670

center number	atomlabel	x	y	z

1	C	-3.783425	-0.370063	-0.149162
2	C	-1.479046	-0.278653	0.231156
3	O	-1.675234	0.151020	1.348706
4	C	-0.107238	-0.591300	-0.325138
5	N	0.846133	0.449916	0.024491
6	C	0.488115	-1.834742	0.345083
7	C	1.973419	-1.550214	0.320057
8	C	2.061167	-0.076029	0.642227
9	C	0.572945	1.738891	-0.241822
10	O	-0.490802	2.051054	-0.795428
11	C	1.608751	2.744044	0.157116
12	H	-3.927596	0.682606	0.083807
13	H	-4.439889	-0.671807	-0.957457
14	H	-3.970838	-0.971976	0.737099
15	H	-0.184325	-0.706389	-1.405925
16	H	0.163465	-1.882936	1.385104
17	H	0.228619	-2.759702	-0.160631
18	H	2.565128	-2.178737	0.978203
19	F	2.433544	-1.742575	-0.986151
20	H	2.957044	0.369926	0.216043
21	H	2.063809	0.075432	1.722599
22	H	1.219795	3.744143	-0.005983
23	H	1.883088	2.616957	1.204338
24	H	2.511011	2.602258	-0.439314
25	O	-2.446746	-0.586776	-0.619662

7.34 *N*-Acetyl-(4*R*)-FPro-OMe **6a**, *trans*-rotamer, *exo*-pucker, psi2-rotamer, solvent = water

SCF energy (Hartree) = -691.905080

center number	atomlabel	x	y	z
1	C	-3.235982	-0.291947	1.025464
2	C	-1.424914	-0.723029	-0.387564
3	O	-2.177864	-1.199553	-1.211223
4	C	0.075770	-0.678669	-0.561781
5	N	0.678075	0.528815	-0.018551
6	C	0.763436	-1.793582	0.237162
7	C	2.114188	-1.192498	0.556752
8	C	1.797950	0.250225	0.877231
9	C	0.189615	1.739329	-0.339604
10	O	-0.770233	1.840415	-1.116639
11	C	0.862943	2.924034	0.281361
12	H	-3.596485	-1.317546	0.994378
13	H	-3.386485	0.132091	2.011908
14	H	-3.751700	0.302750	0.274521
15	H	0.270394	-0.767888	-1.630368
16	H	0.231360	-1.965770	1.173122
17	H	0.838554	-2.726384	-0.313036
18	H	2.663110	-1.705422	1.340229
19	F	2.892082	-1.223364	-0.604546
20	H	2.648398	0.897657	0.675464
21	H	1.511397	0.349796	1.925402

22	H	0.316468	3.824824	0.020526
23	H	0.905513	2.815119	1.365240
24	H	1.888461	3.000857	-0.081919
25	O	-1.823438	-0.257698	0.788008

7.35 *N*-Acetyl-(4*R*)-FPro-OMe **6a**, *trans*-rotamer, *endo*-pucker, psi1-rotamer, solvent = water

SCF energy (Hartree) = -691.902433

center number	atomlabel	x	y	z
1	C	-3.699696	-0.463478	0.332040
2	C	-1.388123	-0.176991	0.121593
3	O	-1.301132	0.044387	1.311471
4	C	-0.205192	-0.258639	-0.824481
5	N	0.883918	0.575710	-0.345990
6	C	0.399712	-1.663675	-0.808504
7	C	1.387016	-1.583350	0.340979
8	C	1.994373	-0.194318	0.202602
9	C	0.685806	1.901601	-0.205637
10	O	-0.381870	2.411138	-0.569617
11	C	1.799944	2.697643	0.399480
12	H	-3.845612	0.507775	0.799029
13	H	-4.529881	-0.695599	-0.325347
14	H	-3.596052	-1.228749	1.097863
15	H	-0.539025	0.038430	-1.815447
16	H	-0.345188	-2.446555	-0.693519
17	H	0.957046	-1.825561	-1.730985
18	H	0.896865	-1.707476	1.305301
19	F	2.365175	-2.557348	0.237796
20	H	2.837280	-0.241210	-0.490453
21	H	2.332804	0.212607	1.151557
22	H	2.757823	2.438187	-0.049581
23	H	1.597159	3.755437	0.263985
24	H	1.863111	2.477576	1.466518
25	O	-2.529919	-0.436957	-0.497551

7.36 *N*-Acetyl-(4*R*)-FPro-OMe **6a**, *trans*-rotamer, *endo*-pucker, psi2-rotamer, solvent = water

SCF energy (Hartree) = -691.901520

center number	atomlabel	x	y	z
1	C	-2.503387	-1.203171	1.515325
2	C	-1.259423	-0.874011	-0.437687
3	O	-2.110403	-1.452887	-1.080184
4	C	0.023604	-0.348613	-1.052227
5	N	0.523032	0.836220	-0.370209
6	C	1.168765	-1.352114	-0.894996
7	C	1.829084	-0.919567	0.401335
8	C	1.768079	0.600351	0.350666
9	C	-0.283462	1.908032	-0.243492
10	O	-1.402250	1.904867	-0.773977

11	C	0.230360	3.063914	0.556669
12	H	-2.576718	-2.278383	1.369546
13	H	-2.397102	-0.969710	2.568674
14	H	-3.384444	-0.714698	1.105263
15	H	-0.203452	-0.146036	-2.096174
16	H	0.830556	-2.384730	-0.891309
17	H	1.883736	-1.209942	-1.705118
18	H	1.309623	-1.309719	1.274545
19	F	3.142525	-1.352811	0.466450
20	H	2.633883	0.972513	-0.201531
21	H	1.750335	1.054078	1.337665
22	H	1.254776	3.310930	0.281526
23	H	-0.416645	3.921210	0.399072
24	H	0.224924	2.800461	1.615756
25	O	-1.324238	-0.698623	0.874403

7.37 *N*-acetyl-(4*R*)-FPro-OMe **6a**, *cis*-rotamer, *exo*-pucker, psi1-rotamer, solvent = water

SCF energy (Hartree) = -691.903486

center number	atomlabel	x	y	z
1	C	-3.770410	-0.529127	-0.065067
2	C	-1.472730	-0.188341	0.210194
3	O	-1.674169	0.390963	1.255854
4	C	-0.102827	-0.492178	-0.352614
5	N	0.907062	0.462013	0.078433
6	C	0.397406	-1.821136	0.240061
7	C	1.893832	-1.621642	0.301664
8	C	2.043002	-0.182849	0.733108
9	C	0.868693	1.795714	-0.110544
10	O	1.765593	2.520575	0.338320
11	C	-0.289897	2.365775	-0.874680
12	H	-4.416825	-0.992700	-0.801526
13	H	-3.874104	-1.024270	0.897580
14	H	-4.006592	0.527599	0.036149
15	H	-0.168881	-0.549583	-1.438753
16	H	0.024155	-1.939088	1.257884
17	H	0.107436	-2.682391	-0.353732
18	H	2.415013	-2.329818	0.938246
19	F	2.406418	-1.747899	-0.992999
20	H	2.982112	0.249220	0.395991
21	H	1.984616	-0.099857	1.819320
22	H	-1.088354	2.618640	-0.176328
23	H	0.039840	3.279032	-1.362484
24	H	-0.687714	1.677756	-1.619047
25	O	-2.431463	-0.685893	-0.556434

7.38 *N*-Acetyl-(4*R*)-FPro-OMe **6a**, *cis*-rotamer, *exo*-pucker, psi2-rotamer, solvent = water

SCF energy (Hartree) = -691.903181

center number	atomlabel	x	y	z
1	C	3.182794	-0.534598	-1.055862
2	C	1.360779	-0.778542	0.388344
3	O	2.086782	-1.252879	1.236375
4	C	-0.128388	-0.600030	0.581009
5	N	-0.677318	0.588858	-0.058747
6	C	-0.883651	-1.744795	-0.113845
7	C	-2.199707	-1.103397	-0.487941
8	C	-1.805209	0.283180	-0.935417
9	C	-0.237095	1.850919	0.112360
10	O	-0.747221	2.787129	-0.515062
11	C	0.888947	2.067950	1.080666
12	H	3.348758	-0.170840	-2.063464
13	H	3.736466	0.067548	-0.337854
14	H	3.483069	-1.576423	-0.972636
15	H	-0.314708	-0.599494	1.654761
16	H	-0.366146	-2.030076	-1.030132
17	H	-1.001909	-2.616837	0.521742
18	H	-2.776347	-1.656594	-1.222615
19	F	-2.975205	-0.987735	0.669884
20	H	-2.610799	1.000762	-0.798891
21	H	-1.509452	0.278114	-1.985603
22	H	1.840887	1.867263	0.584100
23	H	0.879921	3.108212	1.392608
24	H	0.814771	1.421672	1.954120
25	O	1.775780	-0.405477	-0.812532

7.39 *N*-acetyl-(4*R*)-FPro-OMe **6a**, *cis*-rotamer, *endo*-pucker, psi1-rotamer, solvent = water

SCF energy (Hartree) = -691.901148

center number	atomlabel	x	y	z
1	C	3.686318	-0.331242	-0.340380
2	C	1.377233	-0.011080	-0.138450
3	O	1.301795	0.279048	-1.313772
4	C	0.188362	-0.097259	0.803989
5	N	-0.990512	0.516809	0.222451
6	C	-0.243149	-1.555406	0.964086
7	C	-1.103150	-1.768995	-0.268359
8	C	-1.855678	-0.454636	-0.439057
9	C	-1.166624	1.842565	0.042846
10	O	-2.143612	2.266590	-0.584348
11	C	-0.147878	2.756740	0.656749
12	H	3.579324	-1.058376	-1.141921
13	H	3.852680	0.658410	-0.759259
14	H	4.504213	-0.607434	0.315225
15	H	0.466232	0.350986	1.754954
16	H	0.594824	-2.242190	1.040479
17	H	-0.873616	-1.643873	1.848447
18	H	-0.500193	-2.001174	-1.144951
19	F	-1.988852	-2.818983	-0.087431

20	H	-2.825981	-0.523302	0.055655
21	H	-2.012943	-0.186551	-1.481624
22	H	-0.441290	3.784733	0.468934
23	H	-0.079558	2.586519	1.731181
24	H	0.839842	2.582460	0.227722
25	O	2.506456	-0.324531	0.476411

7.40 *N*-Acetyl-(4*R*)-FPro-OMe **6a**, *cis*-rotamer, *endo*-pucker, psi2-rotamer, solvent = water

SCF energy (Hartree) = -691.900286

center number	atomlabel	x	y	z
1	C	-2.310911	-1.629050	1.458750
2	C	-1.065799	-1.056026	-0.435887
3	O	-1.646804	-1.901839	-1.082820
4	C	0.039644	-0.196849	-1.028302
5	N	0.401788	0.945938	-0.209367
6	C	1.344003	-0.993234	-1.072943
7	C	1.903326	-0.771267	0.320709
8	C	1.554216	0.677155	0.644571
9	C	-0.368284	2.036618	-0.015824
10	O	-0.039638	2.900215	0.805110
11	C	-1.610650	2.148749	-0.848522
12	H	-3.262410	-1.473137	0.955734
13	H	-2.030926	-2.678282	1.403014
14	H	-2.370000	-1.305237	2.491438
15	H	-0.287464	0.095265	-2.023425
16	H	1.195198	-2.038392	-1.328353
17	H	2.016269	-0.529844	-1.794647
18	H	1.467953	-1.459181	1.043791
19	F	3.275693	-0.960309	0.347279
20	H	2.396634	1.321553	0.388294
21	H	1.303128	0.829887	1.692292
22	H	-2.101933	3.089961	-0.623002
23	H	-1.367235	2.106869	-1.909985
24	H	-2.295178	1.326999	-0.631765
25	O	-1.297441	-0.818604	0.845381

7.41 *N*-Acetyl-(4*R*)-FPro-NMe_2 **6b**, *trans*-rotamer, *exo*-pucker, psi1-rotamer, solvent=octanol

SCF energy (Hartree) = -711.311911

center number	atomlabel	x	y	z
1	C	-3.657458	0.054879	0.567859
2	N	-2.426140	-0.323368	-0.101969
3	C	-2.532014	-0.658028	-1.515781
4	C	-1.252833	-0.150496	0.526282
5	O	-1.155204	0.263866	1.684169
6	C	0.022102	-0.560131	-0.207066
7	N	1.062139	0.433014	0.024370
8	C	0.611009	-1.830638	0.419769

9	C	2.104354	-1.597253	0.354907
10	C	2.244427	-0.124003	0.665834
11	C	0.851734	1.715267	-0.322069
12	O	-0.205209	2.050785	-0.866967
13	C	1.953048	2.688581	-0.019691
14	H	-4.030624	1.006069	0.182145
15	H	-4.414070	-0.710868	0.400050
16	H	-3.475316	0.153363	1.632797
17	H	-3.587550	-0.752007	-1.759271
18	H	-2.103887	0.124033	-2.144773
19	H	-2.052883	-1.608686	-1.744996
20	H	-0.137536	-0.673193	-1.276524
21	H	0.317754	-1.892025	1.468584
22	H	0.310580	-2.741107	-0.092516
23	H	2.692305	-2.242340	1.001891
24	F	2.529066	-1.808547	-0.959906
25	H	3.164006	0.284494	0.251911
26	H	2.231924	0.033888	1.747106
27	H	1.612945	3.693034	-0.253357
28	H	2.240952	2.631395	1.030416
29	H	2.834906	2.457070	-0.619003

7.42 *N*-Acetyl-(4R)-FPro-NMe$_2$ **6b**, *trans*-rotamer, *exo*-pucker, psi2-rotamer, solvent=octanol

SCF energy (Hartree) = -711.308943

center number	atomlabel	x	y	z
1	C	-3.475439	-0.234627	0.414939
2	N	-2.025274	-0.267274	0.486688
3	C	-1.471355	0.366260	1.668753
4	C	-1.326830	-0.782165	-0.542320
5	O	-1.871537	-1.361927	-1.485385
6	C	0.197496	-0.726864	-0.557125
7	N	0.824010	0.497090	-0.073193
8	C	0.846822	-1.815617	0.305868
9	C	2.206606	-1.229225	0.618237
10	C	1.921146	0.238826	0.851529
11	C	0.434250	1.703687	-0.532572
12	O	-0.474133	1.798851	-1.360348
13	C	1.150755	2.898267	0.027110
14	H	-3.913677	-0.929270	1.134231
15	H	-3.823768	0.772504	0.648285
16	H	-3.800264	-0.504325	-0.584025
17	H	-2.159720	0.190028	2.495301
18	H	-0.511959	-0.054570	1.951286
19	H	-1.368242	1.447201	1.537801
20	H	0.450783	-0.875439	-1.607685
21	H	0.299527	-1.958136	1.238879
22	H	0.914943	-2.770469	-0.207711
23	H	2.731979	-1.709733	1.438830
24	F	3.003218	-1.339843	-0.523359

25	H	2.794398	0.848526	0.628928
26	H	1.628421	0.406378	1.892442
27	H	0.647280	3.800740	-0.306511
28	H	1.170593	2.866416	1.117425
29	H	2.184243	2.913375	-0.322350

7.43 *N*-Acetyl-(4R)-FPro-NMe$_2$ **6b**, *trans*-rotamer, *endo*-pucker, psi1-rotamer, solvent=octanol

SCF energy (Hartree) = -711.309470

center number	atomlabel	x	y	z
1	C	-3.491032	0.147044	1.002082
2	N	-2.429700	-0.105804	0.045255
3	C	-2.854423	-0.413792	-1.311419
4	C	-1.143972	0.038511	0.404276
5	O	-0.782786	0.300896	1.553827
6	C	-0.093376	-0.197886	-0.685206
7	N	1.142807	0.474697	-0.328093
8	C	0.323315	-1.668877	-0.722929
9	C	1.379751	-1.735388	0.365461
10	C	2.147011	-0.426068	0.219883
11	C	1.176491	1.822725	-0.288750
12	O	0.198930	2.484851	-0.645445
13	C	2.446124	2.451390	0.203376
14	H	-4.159034	0.918292	0.615623
15	H	-4.071472	-0.760320	1.175969
16	H	-3.061546	0.483538	1.939670
17	H	-3.864498	-0.816496	-1.265551
18	H	-2.867638	0.476304	-1.943538
19	H	-2.221987	-1.170382	-1.770577
20	H	-0.452722	0.163807	-1.644847
21	H	-0.504787	-2.355210	-0.560517
22	H	0.799085	-1.893447	-1.678507
23	H	0.935988	-1.827486	1.355047
24	F	2.224794	-2.818627	0.184431
25	H	2.978454	-0.578128	-0.473318
26	H	2.535922	-0.065940	1.170153
27	H	3.322132	1.993183	-0.255335
28	H	2.424682	3.514983	-0.015224
29	H	2.524031	2.310756	1.283232

7.44 *N*-Acetyl-(4*R*)-FPro-NMe$_2$ **6b**, *trans*-rotamer, *endo*-pucker, psi2-rotamer, solvent=octanol

SCF energy (Hartree) = -711.303022

center number	atomlabel	x	y	z
1	C	-3.306241	-0.550056	0.791128
2	N	-1.886745	-0.298562	0.587050
3	C	-1.241269	0.414948	1.679157
4	C	-1.327891	-0.770417	-0.540398
5	O	-1.953867	-1.504976	-1.312804

6	C	0.097328	-0.439937	-0.992637
7	N	0.804806	0.649625	-0.341035
8	C	1.050219	-1.623156	-0.816374
9	C	1.740221	-1.305215	0.494321
10	C	1.950313	0.203715	0.439997
11	C	0.393761	1.926401	-0.495296
12	O	-0.602544	2.188271	-1.171538
13	C	1.191285	2.975205	0.221116
14	H	-3.466818	-1.449296	1.389576
15	H	-3.737951	0.301327	1.315294
16	H	-3.804334	-0.671844	-0.165369
17	H	-1.773503	0.162319	2.595420
18	H	-0.208989	0.115514	1.820449
19	H	-1.296217	1.497394	1.543484
20	H	-0.042639	-0.207682	-2.047686
21	H	0.537722	-2.580967	-0.826504
22	H	1.801525	-1.608579	-1.606855
23	H	1.129981	-1.594763	1.350881
24	F	2.952777	-1.960659	0.612420
25	H	2.895134	0.412946	-0.068032
26	H	1.971062	0.661923	1.428367
27	H	2.253547	2.889397	-0.008978
28	H	0.828328	3.957571	-0.065545
29	H	1.076672	2.847945	1.300257

7.45 *N*-Acetyl-(4*R*)-FPro-NMe$_2$ **6b**, *cis*-rotamer, *exo*-pucker, psi1-rotamer, solvent=octanol

SCF energy (Hartree) = -711.310676

center number	atomlabel	x	y	z
1	C	3.664183	0.138432	-0.506820
2	N	2.411121	-0.310095	0.073595
3	C	2.514138	-1.094857	1.295636
4	C	1.244236	0.029389	-0.496677
5	O	1.158016	0.670182	-1.545599
6	C	-0.030927	-0.455913	0.189936
7	N	-1.145453	0.450113	-0.049166
8	C	-0.499524	-1.762546	-0.471012
9	C	-2.006784	-1.663070	-0.411842
10	C	-2.273443	-0.204739	-0.698424
11	C	-1.185462	1.756627	0.281749
12	O	-2.163532	2.452525	-0.001188
13	C	0.004814	2.327357	1.000768
14	H	4.243821	-0.712483	-0.867846
15	H	3.461005	0.810122	-1.333960
16	H	4.250752	0.663056	0.248413
17	H	2.319820	-0.492031	2.185185
18	H	1.839301	-1.948373	1.284629
19	H	3.528768	-1.481805	1.361513
20	H	0.115935	-0.591461	1.260624
21	H	-0.195376	-1.774169	-1.518768
22	H	-0.116452	-2.651360	0.024538

23	H	-2.529902	-2.348153	-1.073073
24	F	-2.413820	-1.936580	0.897470
25	H	-3.216412	0.132975	-0.274402
26	H	-2.282290	-0.019664	-1.774894
27	H	0.747443	2.642612	0.266339
28	H	-0.319192	3.200917	1.560222
29	H	0.474223	1.613178	1.677661

7.46 *N*-Acetyl-(4R)-FPro-NMe$_2$ **6b**, *cis*-rotamer, *exo*-pucker, psi2-rotamer, solvent=octanol

SCF energy (Hartree) = -711.309137

center number	atomlabel	x	y	z
1	C	-3.416868	-0.452681	0.441118
2	N	-1.965852	-0.420433	0.511948
3	C	-1.438979	0.142528	1.743312
4	C	-1.250009	-0.824010	-0.552398
5	O	-1.770897	-1.356798	-1.536338
6	C	0.265851	-0.650952	-0.585525
7	N	0.820024	0.571752	-0.012378
8	C	0.997727	-1.760978	0.183520
9	C	2.317466	-1.111511	0.536530
10	C	1.939108	0.309708	0.885270
11	C	0.440463	1.836060	-0.297121
12	O	0.968248	2.793184	0.273187
13	C	-0.653493	2.023674	-1.309919
14	H	-3.809975	0.495481	0.810494
15	H	-3.733755	-0.599582	-0.586056
16	H	-3.818902	-1.258795	1.057517
17	H	-2.107977	-0.145073	2.553981
18	H	-0.454127	-0.241619	1.985019
19	H	-1.398653	1.234687	1.705144
20	H	0.516227	-0.716313	-1.645401
21	H	0.469849	-2.018479	1.102634
22	H	1.121645	-2.662153	-0.410451
23	H	2.879392	-1.622006	1.313575
24	F	3.108686	-1.081679	-0.614701
25	H	2.754495	1.006123	0.702913
26	H	1.645531	0.388406	1.935649
27	H	-1.624989	1.934479	-0.817994
28	H	-0.570094	3.026795	-1.719312
29	H	-0.612442	1.291807	-2.115906

7.47 *N*-Acetyl-(4*R*)-FPro-NMe$_2$ **6b**, *cis*-rotamer, *endo*-pucker, psi1-rotamer, solvent=octanol

SCF energy (Hartree) = -711.308526

center number	atomlabel	x	y	z
1	C	-3.432444	-0.276893	-1.025930
2	N	-2.405468	-0.095424	-0.014780

3	C	-2.886321	0.415342	1.255664
4	C	-1.114973	-0.213943	-0.372082
5	O	-0.768104	-0.514584	-1.516058
6	C	-0.044073	0.061784	0.692896
7	N	1.254674	-0.401751	0.240000
8	C	0.187712	1.567944	0.834958
9	C	1.114453	1.858242	-0.332773
10	C	2.040292	0.649598	-0.390848
11	C	1.637221	-1.693429	0.154501
12	O	2.720703	-1.996917	-0.347429
13	C	0.695839	-2.719512	0.718816
14	H	-3.086731	-0.969869	-1.786576
15	H	-4.325681	-0.677938	-0.549018
16	H	-3.687381	0.672603	-1.502242
17	H	-2.094269	0.498463	1.991470
18	H	-3.334737	1.402364	1.119914
19	H	-3.650354	-0.255169	1.650187
20	H	-0.310635	-0.406999	1.637152
21	H	-0.727576	2.154842	0.813304
22	H	0.726177	1.767792	1.762204
23	H	0.562480	1.983259	-1.263135
24	F	1.835748	3.022966	-0.121975
25	H	2.951279	0.858472	0.174293
26	H	2.315854	0.371183	-1.406491
27	H	1.132626	-3.704654	0.584910
28	H	0.524604	-2.541504	1.781162
29	H	-0.268854	-2.682114	0.210778

7.48 *N*-Acetyl-(4*R*)-FPro-NMe$_2$ **6b**, *cis*-rotamer, *endo*-pucker, psi2-rotamer, solvent=octanol

SCF energy (Hartree) = -711.303036

center number	atomlabel	x	y	z
1	C	-3.245377	-0.853558	0.772011
2	N	-1.854826	-0.461991	0.587672
3	C	-1.253971	0.203360	1.736542
4	C	-1.258854	-0.797011	-0.569402
5	O	-1.820713	-1.508651	-1.408625
6	C	0.133753	-0.303789	-0.986095
7	N	0.789157	0.743066	-0.218293
8	C	1.161522	-1.437209	-0.947754
9	C	1.810048	-1.252479	0.410107
10	C	1.921873	0.258719	0.558970
11	C	0.464254	2.054184	-0.222956
12	O	1.105659	2.861033	0.451703
13	C	-0.699507	2.462803	-1.079748
14	H	-3.730812	-0.968628	-0.191699
15	H	-3.319661	-1.795049	1.319885
16	H	-3.753110	-0.076489	1.341499
17	H	-1.328597	1.291124	1.669688
18	H	-1.800448	-0.117968	2.621746
19	H	-0.217808	-0.079880	1.883100
20	H	-0.027905	0.020695	-2.013517

21	H	0.713646	-2.415199	-1.098837
22	H	1.920616	-1.264412	-1.711513
23	H	1.208361	-1.694287	1.205774
24	F	3.059952	-1.843791	0.464704
25	H	2.867875	0.599329	0.131730
26	H	1.867322	0.601081	1.591744
27	H	-0.892865	3.520085	-0.925168
28	H	-0.473823	2.287672	-2.132868
29	H	-1.594697	1.889346	-0.834235

7.49 *N*-Acetyl-(4*R*)-FPro-NMe_2 **6b**, *trans*-rotamer, *exo*-pucker, psi1-rotamer, solvent = water

SCF energy (Hartree) = -711.311801

center number	atomlabel	x	y	z
1	C	-3.685485	0.381654	0.461508
2	N	-2.458493	-0.168340	-0.089109
3	C	-2.609167	-0.996855	-1.279217
4	C	-1.275497	0.114816	0.470051
5	O	-1.153401	0.776467	1.509532
6	C	-0.035931	-0.480005	-0.188635
7	N	1.120411	0.381323	0.008687
8	C	0.370440	-1.776713	0.525249
9	C	1.881208	-1.752097	0.466334
10	C	2.220490	-0.295832	0.686540
11	C	1.099929	1.656363	-0.410980
12	O	0.110192	2.109402	-1.004648
13	C	2.321444	2.479497	-0.133786
14	H	-4.240631	0.890637	-0.326204
15	H	-4.312155	-0.412510	0.868139
16	H	-3.449122	1.089926	1.247638
17	H	-3.646388	-1.318628	-1.331131
18	H	-2.373359	-0.445306	-2.189980
19	H	-1.989938	-1.889487	-1.227131
20	H	-0.195105	-0.634626	-1.254298
21	H	0.067223	-1.731053	1.572173
22	H	-0.052700	-2.666935	0.067425
23	H	2.372280	-2.427112	1.160527
24	F	2.277885	-2.104311	-0.827763
25	H	3.183932	-0.040267	0.251328
26	H	2.237577	-0.072754	1.755205
27	H	2.132356	3.508245	-0.424076
28	H	2.584196	2.433593	0.923080
29	H	3.168531	2.092271	-0.701321

7.50 *N*-Acetyl-(4*R*)-FPro-NMe_2 **6b**, *trans*-rotamer, *endo*-pucker, psi1 rotamer, solvent = water

SCF energy (Hartree) = -711.309056

center number	atomlabel	x	y	z
1	C	-3.482814	0.160982	1.003070
2	N	-2.421392	-0.109496	0.048382

3	C	-2.853898	-0.486026	-1.291189
4	C	-1.137099	0.045352	0.393215
5	O	-0.769435	0.348693	1.536446
6	C	-0.093680	-0.214837	-0.694970
7	N	1.143647	0.474478	-0.366379
8	C	0.332522	-1.682902	-0.702793
9	C	1.391586	-1.720372	0.383233
10	C	2.149876	-0.409680	0.211893
11	C	1.158086	1.819433	-0.304599
12	O	0.165337	2.476493	-0.646480
13	C	2.418215	2.461424	0.188362
14	H	-4.183820	0.876479	0.573408
15	H	-4.025064	-0.754573	1.240497
16	H	-3.061161	0.574739	1.912243
17	H	-3.858557	-0.895696	-1.215465
18	H	-2.881230	0.373075	-1.962754
19	H	-2.216516	-1.255672	-1.718839
20	H	-0.465435	0.117780	-1.659995
21	H	-0.490749	-2.370443	-0.526142
22	H	0.807406	-1.920050	-1.655171
23	H	0.952175	-1.800356	1.375487
24	F	2.245327	-2.800232	0.218796
25	H	2.987224	-0.570671	-0.470318
26	H	2.528471	-0.022662	1.155046
27	H	3.298670	2.005794	-0.262755
28	H	2.389973	3.523604	-0.034396
29	H	2.490902	2.324143	1.268912

7.51 *N*-Acetyl-(4*R*)-FPro-NMe$_2$ **6b**, *trans*-rotamer, *exo*-pucker, psi2-rotamer, solvent = water

SCF energy (Hartree) = -711.309522

center number	atomlabel	x	y	z
1	C	-3.459884	-0.279411	0.444591
2	N	-2.006907	-0.314830	0.500905
3	C	-1.446185	0.339460	1.671741
4	C	-1.312194	-0.791663	-0.543811
5	O	-1.861187	-1.349188	-1.505644
6	C	0.211673	-0.731936	-0.567191
7	N	0.828231	0.506195	-0.106045
8	C	0.875387	-1.802406	0.308515
9	C	2.224653	-1.193065	0.621411
10	C	1.921084	0.273140	0.833391
11	C	0.395431	1.703685	-0.537414
12	O	-0.517069	1.781668	-1.371580
13	C	1.064486	2.909894	0.048057
14	H	-3.887224	-0.946916	1.193498
15	H	-3.801195	0.735222	0.651950
16	H	-3.801224	-0.579229	-0.539412
17	H	-2.128954	0.171236	2.503192
18	H	-0.484125	-0.073186	1.953613
19	H	-1.350721	1.417994	1.523213
20	H	0.461524	-0.895223	-1.615965

21	H	0.329050	-1.946411	1.240754
22	H	0.961734	-2.759420	-0.197470
23	H	2.750133	-1.655776	1.450924
24	F	3.030060	-1.310397	-0.515917
25	H	2.786242	0.892891	0.609292
26	H	1.615349	0.451932	1.867447
27	H	0.549123	3.803733	-0.289094
28	H	1.052379	2.861305	1.137802
29	H	2.107440	2.949831	-0.268454

7.52 *N*-Acetyl-(4*R*)-FPro-NMe$_2$ **6b**, *trans*-rotamer, *endo*-pucker, psi2-rotamer, solvent = water

SCF energy (Hartree) = -711.303507

center number	atomlabel	x	y	z
1	C	-3.289244	-0.595356	0.802223
2	N	-1.869474	-0.309610	0.615051
3	C	-1.213176	0.426773	1.689253
4	C	-1.334578	-0.731208	-0.538488
5	O	-2.000972	-1.419269	-1.329832
6	C	0.093284	-0.437277	-1.001265
7	N	0.823491	0.649093	-0.369889
8	C	1.023637	-1.636324	-0.807842
9	C	1.737093	-1.311095	0.489198
10	C	1.960646	0.194859	0.422098
11	C	0.400565	1.921549	-0.489469
12	O	-0.607567	2.188882	-1.156697
13	C	1.189913	2.962380	0.241602
14	H	-3.456716	-1.653712	1.003184
15	H	-3.651150	-0.012167	1.643679
16	H	-3.852698	-0.323350	-0.087667
17	H	-1.728832	0.188135	2.616219
18	H	-0.178469	0.132721	1.818543
19	H	-1.271578	1.505749	1.535758
20	H	-0.042015	-0.223029	-2.060240
21	H	0.492024	-2.583333	-0.792714
22	H	1.765442	-1.650461	-1.606586
23	H	1.142637	-1.591011	1.358700
24	F	2.947740	-1.976113	0.586439
25	H	2.908294	0.392976	-0.083121
26	H	1.978719	0.661640	1.405592
27	H	2.253832	2.874572	0.023037
28	H	0.831238	3.948172	-0.037108
29	H	1.060957	2.819031	1.317005

7.53 *N*-Acetyl-(4*R*)-FPro-NMe$_2$ **6b**, *cis*-rotamer, *endo*-pucker, psi1-rotamer, solvent = water

SCF energy (Hartree) = -711.308979

center number	atomlabel	x	y	z
1	C	-3.370489	-0.296068	-1.075689

2	N	-2.388814	-0.118914	-0.015068
3	C	-2.893240	0.431336	1.232896
4	C	-1.095636	-0.233297	-0.342624
5	O	-0.736269	-0.552715	-1.484420
6	C	-0.040052	0.067459	0.726542
7	N	1.267822	-0.388202	0.284852
8	C	0.175571	1.576420	0.848434
9	C	1.080621	1.864418	-0.336132
10	C	2.006163	0.655745	-0.417009
11	C	1.626233	-1.682032	0.162474
12	O	2.683080	-1.992857	-0.400670
13	C	0.708412	-2.704799	0.764275
14	H	-3.168191	-1.207988	-1.631735
15	H	-4.356110	-0.365507	-0.623184
16	H	-3.357247	0.544632	-1.770831
17	H	-2.133968	0.440386	2.006817
18	H	-3.250604	1.451632	1.083298
19	H	-3.726180	-0.177391	1.580772
20	H	-0.308539	-0.391155	1.674352
21	H	-0.746367	2.151835	0.834984
22	H	0.724970	1.789130	1.765624
23	H	0.511965	1.994959	-1.255034
24	F	1.810214	3.027451	-0.137458
25	H	2.948019	0.882333	0.085049
26	H	2.220251	0.354982	-1.441098
27	H	1.142748	-3.690375	0.628976
28	H	0.565292	-2.511034	1.827339
29	H	-0.271007	-2.674187	0.285026

7.54 *N*-Acetyl-(4*R*)-FPro-NMe$_2$ **6b**, *cis*-rotamer, *exo*-pucker, psi1-rotamer, solvent = water

SCF energy (Hartree) = -711.310726

center number	atomlabel	x	y	z
1	C	3.666432	0.111856	-0.520834
2	N	2.406786	-0.315204	0.065877
3	C	2.503457	-1.068269	1.311600
4	C	1.241834	0.034073	-0.492211
5	O	1.150115	0.676418	-1.546699
6	C	-0.029329	-0.452540	0.196680
7	N	-1.150287	0.450800	-0.035252
8	C	-0.491969	-1.758297	-0.469781
9	C	-1.999198	-1.667205	-0.414667
10	C	-2.273996	-0.208874	-0.691377
11	C	-1.180811	1.757657	0.279834
12	O	-2.155525	2.461291	-0.020700
13	C	0.002356	2.328942	1.005823
14	H	4.255538	-0.754975	-0.819719
15	H	3.475202	0.732954	-1.388435
16	H	4.237263	0.682132	0.212032
17	H	2.259067	-0.450146	2.176627
18	H	1.859984	-1.945397	1.301351

19	H	3.529441	-1.412135	1.414125
20	H	0.121242	-0.591064	1.265390
21	H	-0.187323	-1.765109	-1.517088
22	H	-0.103075	-2.645089	0.023686
23	H	-2.515945	-2.348298	-1.083791
24	F	-2.411598	-1.953041	0.891080
25	H	-3.220699	0.118051	-0.268124
26	H	-2.281598	-0.019788	-1.766525
27	H	0.746125	2.653172	0.276805
28	H	-0.327159	3.196971	1.570112
29	H	0.471453	1.611196	1.678252

7.55 *N*-Acetyl-(4*R*)-FPro-NMe_2 **6b**, *cis*-rotamer, *exo*-pucker, psi2-rotamer, solvent = water

SCF energy (Hartree) = -711.308414

center number	atomlabel	x	y	z
1	C	-3.385902	-0.490072	0.466828
2	N	-1.933275	-0.512085	0.538808
3	C	-1.404136	0.118898	1.737405
4	C	-1.222701	-0.861495	-0.545511
5	O	-1.742047	-1.380371	-1.543409
6	C	0.289300	-0.661247	-0.589749
7	N	0.819332	0.591430	-0.054740
8	C	1.050306	-1.732368	0.205317
9	C	2.353137	-1.043062	0.542166
10	C	1.942619	0.377086	0.852367
11	C	0.359874	1.830775	-0.310626
12	O	0.847723	2.812875	0.264271
13	C	-0.761137	1.974301	-1.297976
14	H	-3.735728	0.532434	0.623184
15	H	-3.716717	-0.840486	-0.503700
16	H	-3.811673	-1.122947	1.245064
17	H	-2.060503	-0.144215	2.565355
18	H	-0.410043	-0.235133	1.985085
19	H	-1.392482	1.208210	1.646149
20	H	0.535469	-0.746276	-1.648558
21	H	0.531843	-1.979817	1.131412
22	H	1.197455	-2.643564	-0.366942
23	H	2.924176	-1.518807	1.333280
24	F	3.147299	-1.025784	-0.609422
25	H	2.744359	1.084637	0.654877
26	H	1.641588	0.474646	1.898360
27	H	-1.716819	1.849205	-0.782529
28	H	-0.726859	2.978889	-1.709958
29	H	-0.705717	1.242932	-2.102975

7.56 *N*-Acetyl-(4*R*)-FPro-NMe_2 **6b**, *cis*-rotamer, *endo*-pucker, psi2-rotamer, solvent = water

SCF energy (Hartree) = -711.302402

center number	atomlabel	x	y	z
1	C	-3.243843	-0.856470	0.763966
2	N	-1.846127	-0.457402	0.619837
3	C	-1.238314	0.242624	1.746765
4	C	-1.283474	-0.729377	-0.564822
5	O	-1.897998	-1.381416	-1.424496
6	C	0.120491	-0.286886	-0.992862
7	N	0.825172	0.733414	-0.231471
8	C	1.101782	-1.461405	-0.958169
9	C	1.765546	-1.303993	0.395886
10	C	1.933187	0.201000	0.551789
11	C	0.524470	2.046526	-0.208065
12	O	1.183665	2.829785	0.486255
13	C	-0.622962	2.498179	-1.061613
14	H	-3.831244	-0.498460	-0.079464
15	H	-3.340740	-1.941000	0.811168
16	H	-3.635261	-0.423798	1.679326
17	H	-1.302312	1.327105	1.640642
18	H	-1.782869	-0.041452	2.643247
19	H	-0.205054	-0.048348	1.898363
20	H	-0.032756	0.044485	-2.018708
21	H	0.613965	-2.420707	-1.103549
22	H	1.861453	-1.318676	-1.726884
23	H	1.156054	-1.726728	1.194852
24	F	2.995865	-1.938499	0.436375
25	H	2.894049	0.506793	0.132960
26	H	1.882436	0.537735	1.586143
27	H	-0.785971	3.559052	-0.900115
28	H	-0.399511	2.319976	-2.114242
29	H	-1.534964	1.950291	-0.820986

N-Acetyl-(4,4)-F_2Pro-OMe **7a** (octanol)

7.57 *N*-Acetyl-(4,4)-F_2Pro-OMe **7a**, *trans*-rotamer, *exo*-pucker, psi1-rotamer, solvent=octanol

SCF energy (Hartree) = -791.120248

center number	atomlabel	x	y	z
1	C	-3.814654	-1.154539	-0.043229
2	C	-1.573937	-0.561864	0.218598
3	O	-1.762042	-0.317046	1.388632
4	C	-0.216145	-0.459021	-0.449276
5	N	0.459496	0.765298	-0.037482
6	C	0.725452	-1.559030	0.045658
7	C	2.067325	-0.868380	0.004613
8	C	1.799636	0.547092	0.478130
9	C	-0.172483	1.951109	-0.175556
10	O	-1.310817	1.987567	-0.644949
11	C	0.577210	3.171206	0.264720

12	H	-4.173930	-0.191747	0.314777
13	H	-4.448658	-1.520973	-0.843585
14	H	-3.792329	-1.865978	0.779946
15	H	-0.349289	-0.485450	-1.530469
16	H	0.506664	-1.798613	1.086563
17	H	0.707060	-2.459267	-0.560785
18	F	3.017805	-1.490960	0.738362
19	F	2.531869	-0.840018	-1.280600
20	H	2.525509	1.242510	0.061852
21	H	1.838527	0.577277	1.568442
22	H	-0.075923	4.035298	0.190886
23	H	0.926853	3.059112	1.291514
24	H	1.452049	3.322412	-0.369400
25	O	-2.506454	-1.007060	-0.607330

7.58 *N*-Acetyl-(4,4)-F_2Pro-OMe **7a**, *trans*-rotamer, *exo*-pucker, psi2-rotamer, solvent=octanol

SCF energy (Hartree) = -791.119532

center number	atomlabel	x	y	z
1	C	-3.183527	-1.033379	1.193772
2	C	-1.504684	-0.912556	-0.423993
3	O	-2.215330	-1.483672	-1.218623
4	C	-0.082109	-0.491772	-0.729709
5	N	0.288479	0.764014	-0.088779
6	C	0.928445	-1.490154	-0.155869
7	C	2.114863	-0.603320	0.136457
8	C	1.524510	0.687155	0.670245
9	C	-0.496552	1.852507	-0.243664
10	O	-1.523547	1.778335	-0.919107
11	C	-0.048252	3.108679	0.439190
12	H	-3.316695	-2.107593	1.081726
13	H	-3.306716	-0.743603	2.232030
14	H	-3.900987	-0.507841	0.566460
15	H	-0.003699	-0.413508	-1.813875
16	H	0.572002	-1.893283	0.792187
17	H	1.176190	-2.298689	-0.836451
18	F	3.007324	-1.157950	0.988164
19	F	2.795011	-0.348884	-1.021104
20	H	2.186804	1.528412	0.477385
21	H	1.351296	0.589111	1.743572
22	H	-0.814992	3.868662	0.323985
23	H	0.137261	2.926702	1.498265
24	H	0.883142	3.463494	-0.004819
25	O	-1.851140	-0.657341	0.830089

7.59 *N*-Acetyl-(4,4)-F_2Pro-OMe **7a**, *trans*-rotamer, *endo*-pucker, psi1-rotamer, solvent=octanol

SCF energy (Hartree) = -791.120023

center number	atomlabel	x	y	z

1	C	-3.772984	-0.149911	0.275549
2	C	-1.468487	0.176284	0.056907
3	O	-1.400986	0.559112	1.200554
4	C	-0.287514	0.050218	-0.895014
5	N	0.896682	0.661282	-0.324705
6	C	0.135228	-1.404734	-1.105172
7	C	1.115951	-1.617400	0.024553
8	C	1.863615	-0.303216	0.177520
9	C	0.955465	2.004742	-0.167709
10	O	0.039415	2.719345	-0.571515
11	C	2.178993	2.542686	0.510527
12	H	-3.951536	0.871680	0.604587
13	H	-4.587453	-0.492014	-0.354145
14	H	-3.664861	-0.799645	1.141769
15	H	-0.571803	0.529359	-1.829832
16	H	-0.686426	-2.115172	-1.071030
17	H	0.670174	-1.501282	-2.047848
18	F	0.435018	-1.899087	1.175676
19	F	1.953080	-2.661874	-0.181005
20	H	2.770661	-0.330827	-0.427712
21	H	2.124463	-0.132917	1.220755
22	H	3.087130	2.142441	0.059449
23	H	2.177257	3.626197	0.440785
24	H	2.172843	2.249234	1.561694
25	O	-2.592392	-0.205881	-0.534107

7.60 *N*-Acetyl-(4,4)-F_2Pro-OMe **7a**, *trans*-rotamer, *endo*-pucker, psi2-rotamer, solvent=octanol

SCF energy (Hartree) = -791.119378

center number	atomlabel	x	y	z
1	C	-2.451182	-1.717707	1.291646
2	C	-1.057676	-1.158816	-0.491968
3	O	-1.359418	-2.185523	-1.059334
4	C	-0.077148	-0.166771	-1.102159
5	N	0.129682	1.026271	-0.302128
6	C	1.326137	-0.766207	-1.194967
7	C	1.908000	-0.384178	0.144880
8	C	1.383288	1.011544	0.437611
9	C	-0.826749	1.980915	-0.224510
10	O	-1.877360	1.866190	-0.853390
11	C	-0.522967	3.152546	0.660098
12	H	-1.973062	-2.682809	1.446828
13	H	-2.734760	-1.277871	2.241783
14	H	-3.324145	-1.839234	0.653854
15	H	-0.477599	0.084138	-2.082642
16	H	1.335104	-1.840460	-1.357558
17	H	1.893965	-0.263887	-1.975624
18	F	1.452904	-1.254415	1.099133
19	F	3.258427	-0.446654	0.191122
20	H	2.092006	1.750447	0.061684

21	H	1.242246	1.151228	1.508361
22	H	0.444093	3.591051	0.411745
23	H	-1.305764	3.896312	0.546731
24	H	-0.481043	2.827199	1.701013
25	O	-1.527098	-0.802960	0.690553

7.61 *N*-Acetyl-(4,4)-F_2Pro-OMe **7a**, *cis*-rotamer, *exo*-pucker, psi1-rotamer, solvent=octanol

SCF energy (Hartree) = -791.117856

center number	atomlabel	x	y	z
1	C	3.745631	-1.326642	0.042351
2	C	1.551003	-0.575955	-0.230933
3	O	1.759829	-0.341947	-1.398669
4	C	0.204834	-0.382372	0.441692
5	N	-0.481327	0.808900	-0.046808
6	C	-0.738551	-1.523500	0.039871
7	C	-2.080823	-0.833423	0.022077
8	C	-1.811958	0.535557	-0.563172
9	C	-0.010317	2.076953	0.029422
10	O	-0.671142	3.016710	-0.408224
11	C	1.341073	2.270592	0.655234
12	H	4.352217	-1.717952	0.851848
13	H	3.662476	-2.057437	-0.759378
14	H	4.176761	-0.405664	-0.345222
15	H	0.343348	-0.348806	1.522169
16	H	-0.521956	-1.852653	-0.976487
17	H	-0.713657	-2.367322	0.722492
18	F	-3.026556	-1.520312	-0.658495
19	F	-2.546260	-0.702444	1.301117
20	H	-2.532603	1.272728	-0.217444
21	H	-1.834160	0.483819	-1.652768
22	H	2.117841	2.055202	-0.081078
23	H	1.433151	3.310632	0.955091
24	H	1.498687	1.625138	1.519174
25	O	2.453461	-1.062569	0.605193

7.62 *N*-Acetyl-(4,4)-F_2Pro-OMe **7a**, *cis*-rotamer, *exo*-pucker, psi2-rotamer, solvent=octanol

SCF energy (Hartree) = -791.117708

center number	atomlabel	x	y	z
1	C	3.084215	-1.274808	-1.201798
2	C	1.474517	-0.898205	0.450058
3	O	2.193264	-1.396815	1.285220
4	C	0.082471	-0.379775	0.753080
5	N	-0.289892	0.803795	-0.015642
6	C	-0.961148	-1.423688	0.329542
7	C	-2.129602	-0.561906	-0.084229

8	C	-1.513567	0.632296	-0.779311
9	C	0.378535	1.981998	-0.025241
10	O	-0.007825	2.913828	-0.727956
11	C	1.599990	2.083115	0.842609
12	H	3.183653	-1.087815	-2.265571
13	H	3.856316	-0.742560	-0.648991
14	H	3.154727	-2.340479	-0.993878
15	H	0.034893	-0.191273	1.825265
16	H	-0.622470	-1.968210	-0.551796
17	H	-1.218673	-2.119712	1.121622
18	F	-3.026722	-1.214846	-0.858188
19	F	-2.809657	-0.145623	1.025927
20	H	-2.149630	1.511756	-0.716343
21	H	-1.321627	0.392825	-1.826732
22	H	2.448155	1.611987	0.340512
23	H	1.831742	3.134547	0.986646
24	H	1.464689	1.601048	1.810402
25	O	1.789863	-0.784340	-0.830957

7.63 *N*-Acetyl-(4,4)-F_2Pro-OMe **7a**, *cis*-rotamer, *endo*-pucker, psi1-rotamer, solvent=octanol

SCF energy (Hartree) = -791.119412

center number	atomlabel	x	y	z
1	C	-3.656423	-0.655794	-0.250002
2	C	-1.327411	-0.580822	-0.059816
3	O	-1.205841	-1.037034	-1.172038
4	C	-0.164652	-0.224389	0.861013
5	N	1.109027	-0.402368	0.193288
6	C	-0.147706	1.263572	1.213714
7	C	0.555873	1.843090	0.008744
8	C	1.625384	0.835081	-0.374584
9	C	1.745033	-1.576705	-0.027630
10	O	2.793674	-1.600539	-0.669401
11	C	1.127408	-2.809299	0.566262
12	H	-3.662688	-0.067307	-1.164990
13	H	-3.669772	-1.715659	-0.495727
14	H	-4.508631	-0.400373	0.370578
15	H	-0.251017	-0.844880	1.751816
16	H	-1.131277	1.699159	1.362238
17	H	0.474297	1.425330	2.091888
18	F	-0.346304	1.972094	-1.014243
19	F	1.059799	3.080779	0.221957
20	H	2.580449	1.111218	0.071889
21	H	1.738224	0.774897	-1.456065
22	H	1.738186	-3.666494	0.299621
23	H	1.077655	-2.725494	1.652798
24	H	0.114363	-2.956844	0.191878
25	O	-2.491517	-0.345083	0.525868

7.64 *N*-Acetyl-(4,4)-F_2Pro-OMe **7a**, *cis*-rotamer, *endo*-pucker, psi2-rotamer, solvent=octanol

SCF energy (Hartree) = -791.119120

center number	atomlabel	x	y	z
1	C	1.861755	-2.344976	-1.251269
2	C	0.709827	-1.332152	0.506988
3	O	0.683190	-2.381362	1.109985
4	C	0.083164	-0.060755	1.071097
5	N	0.130567	1.077641	0.173014
6	C	-1.419428	-0.258828	1.272298
7	C	-1.942751	0.076527	-0.104736
8	C	-1.091827	1.232002	-0.603021
9	C	1.195468	1.881391	-0.053756
10	O	1.125760	2.779312	-0.891152
11	C	2.423461	1.635549	0.773999
12	H	2.650257	-2.723501	-0.604191
13	H	1.100766	-3.110413	-1.388724
14	H	2.269863	-2.039086	-2.208427
15	H	0.586617	0.141934	2.014884
16	H	-1.691839	-1.262796	1.585019
17	H	-1.797610	0.476302	1.979778
18	F	-1.768001	-1.009767	-0.923325
19	F	-3.265925	0.355586	-0.129734
20	H	-1.583010	2.181232	-0.390008
21	H	-0.911551	1.153340	-1.674316
22	H	3.205132	2.314226	0.446445
23	H	2.210001	1.814246	1.828830
24	H	2.769390	0.606808	0.671190
25	O	1.274218	-1.170537	-0.676787

N-Acetyl-(4,4)-F_2Pro-OMe **7a** (water)

7.65 *N*-acetyl-(4,4)-F_2Pro-OMe **7a**, *trans*-rotamer, *exo*-pucker, psi1-rotamer, solvent = water

SCF energy (Hartree) = -791.119006

center number	atomlabel	x	y	z
1	C	-3.821482	-1.162624	-0.044193
2	C	-1.587511	-0.531923	0.218035
3	O	-1.792160	-0.254222	1.380398
4	C	-0.225117	-0.448759	-0.439962
5	N	0.470185	0.769408	-0.041011
6	C	0.696481	-1.558338	0.069927
7	C	2.050522	-0.894605	0.008013
8	C	1.816392	0.533899	0.457465
9	C	-0.136806	1.964908	-0.177929
10	O	-1.278967	2.025619	-0.648514
11	C	0.636398	3.169226	0.258589
12	H	-4.198937	-0.200484	0.294673
13	H	-4.443741	-1.554496	-0.840686
14	H	-3.787390	-1.859420	0.790154

15	H	-0.352903	-0.489146	-1.520969
16	H	0.480174	-1.773761	1.116218
17	H	0.655112	-2.467511	-0.520889
18	F	2.993268	-1.522710	0.747332
19	F	2.508798	-0.901422	-1.280237
20	H	2.546758	1.208341	0.016565
21	H	1.873285	0.585359	1.545229
22	H	-0.003070	4.044420	0.204401
23	H	1.006376	3.039784	1.275696
24	H	1.500892	3.308472	-0.391893
25	O	-2.511337	-1.001513	-0.605003

7.66 *N*-Acetyl-(4,4)-F_2Pro-OMe **7a**, *trans*-rotamer, *exo*-pucker, psi2-rotamer, solvent = water

SCF energy (Hartree) = -791.118359

center number	atomlabel	x	y	z
1	C	-3.176428	-1.054053	1.198026
2	C	-1.501287	-0.908299	-0.425247
3	O	-2.222912	-1.455130	-1.231769
4	C	-0.077828	-0.493946	-0.731807
5	N	0.292467	0.770650	-0.105695
6	C	0.931117	-1.485732	-0.145306
7	C	2.118587	-0.597077	0.135299
8	C	1.533358	0.702369	0.649905
9	C	-0.505070	1.848621	-0.238443
10	O	-1.545749	1.767501	-0.901718
11	C	-0.062349	3.103596	0.445484
12	H	-3.311849	-2.124775	1.063943
13	H	-3.290719	-0.785665	2.242177
14	H	-3.894151	-0.511721	0.586195
15	H	0.003824	-0.429544	-1.816213
16	H	0.573704	-1.873681	0.808099
17	H	1.177007	-2.302584	-0.815818
18	F	3.008412	-1.141800	0.996230
19	F	2.803494	-0.363767	-1.024824
20	H	2.194145	1.540487	0.441686
21	H	1.362589	0.621761	1.724155
22	H	-0.831000	3.861830	0.335718
23	H	0.126977	2.915158	1.502466
24	H	0.868380	3.459294	0.001771
25	O	-1.843079	-0.675857	0.832933

7.67 *N*-Acetyl-(4,4)-F_2Pro-OMe **7a**, *trans*-rotamer, *endo*-pucker, psi1-rotamer, solvent = water

SCF energy (Hartree) = -791.119434

center number	atomlabel	x	y	z
1	C	-3.773971	-0.213808	0.274768
2	C	-1.472812	0.147221	0.052461
3	O	-1.410743	0.525710	1.201072

4	C	-0.292482	0.034676	-0.899583
5	N	0.886725	0.676317	-0.348336
6	C	0.153182	-1.416579	-1.090303
7	C	1.154112	-1.591437	0.026942
8	C	1.885234	-0.266318	0.139276
9	C	0.912174	2.014205	-0.165673
10	O	-0.034736	2.714033	-0.538770
11	C	2.131876	2.574218	0.496834
12	H	-3.967669	0.801779	0.611964
13	H	-4.582925	-0.563828	-0.356367
14	H	-3.650937	-0.870216	1.133158
15	H	-0.591684	0.489778	-1.841251
16	H	-0.655958	-2.138957	-1.030845
17	H	0.676103	-1.519105	-2.038420
18	F	0.494270	-1.854410	1.196708
19	F	2.000039	-2.630327	-0.166096
20	H	2.773109	-0.287599	-0.492540
21	H	2.173974	-0.073563	1.170308
22	H	3.039031	2.195243	0.026915
23	H	2.105810	3.657708	0.438856
24	H	2.149336	2.267305	1.543794
25	O	-2.593012	-0.242643	-0.538683

7.68 *N*-Acetyl-(4,4)-F_2Pro-OMe **7a**, *trans*-rotamer, *endo*-pucker, psi2-rotamer, solvent = water

SCF energy (Hartree) = -791.118239

center number	atomlabel	x	y	z
1	C	-2.401487	-1.743517	1.308605
2	C	-1.034485	-1.168292	-0.494190
3	O	-1.338688	-2.197721	-1.060840
4	C	-0.064975	-0.174636	-1.116279
5	N	0.121165	1.036825	-0.337612
6	C	1.346869	-0.756082	-1.189857
7	C	1.914781	-0.344559	0.146676
8	C	1.368153	1.045893	0.417712
9	C	-0.868485	1.949316	-0.229060
10	O	-1.930306	1.798841	-0.841651
11	C	-0.596723	3.121376	0.661327
12	H	-1.911573	-2.703472	1.454094
13	H	-2.673202	-1.307186	2.262960
14	H	-3.281888	-1.869253	0.682912
15	H	-0.459557	0.049309	-2.105125
16	H	1.371762	-1.832267	-1.334700
17	H	1.910901	-0.256939	-1.974503
18	F	1.468936	-1.209625	1.111600
19	F	3.265511	-0.386947	0.201518
20	H	2.069189	1.790970	0.042376
21	H	1.210891	1.195501	1.484194
22	H	0.364457	3.576600	0.423583
23	H	-1.393030	3.850156	0.548814
24	H	-0.554830	2.786517	1.699138

25	O	-1.492655	-0.819315	0.693975

7.69 *N*-Acetyl-(4,4)-F_2Pro-OMe **7a**, *cis*-rotamer, *exo*-pucker, psi1-rotamer, solvent = water

SCF energy (Hartree) = -791.116592

center number	atomlabel	x	y	z
1	C	3.717646	-1.416205	-0.021157
2	C	1.597229	-0.443613	-0.193292
3	O	1.882728	0.025253	-1.272986
4	C	0.236188	-0.331534	0.460361
5	N	-0.523525	0.820193	-0.012317
6	C	-0.622724	-1.530780	0.033781
7	C	-2.007182	-0.933245	-0.005003
8	C	-1.822601	0.461597	-0.560598
9	C	-0.130283	2.111056	0.053518
10	O	-0.841089	3.003720	-0.419563
11	C	1.182852	2.417522	0.709511
12	H	4.250273	-2.022536	0.702433
13	H	3.615458	-1.949150	-0.963508
14	H	4.236202	-0.474252	-0.183440
15	H	0.364156	-0.311027	1.542083
16	H	-0.368942	-1.841335	-0.979599
17	H	-0.551646	-2.373544	0.713495
18	F	-2.887604	-1.668718	-0.722260
19	F	-2.513867	-0.860314	1.263008
20	H	-2.601188	1.138354	-0.218394
21	H	-1.820578	0.425800	-1.650536
22	H	1.960700	2.446626	-0.053971
23	H	1.111673	3.402929	1.162143
24	H	1.467559	1.689535	1.467111
25	O	2.421800	-1.169004	0.544917

7.70 *N*-Acetyl-(4,4)-F_2Pro-OMe **7a**, *cis*-rotamer, *exo*-pucker, psi2-rotamer, solvent = water

SCF energy (Hartree) = -791.116268

center number	atomlabel	x	y	z
1	C	3.094081	-1.244482	-1.197313
2	C	1.452453	-0.926141	0.436378
3	O	2.161325	-1.447098	1.270060
4	C	0.062597	-0.406012	0.740916
5	N	-0.290424	0.810865	0.012462
6	C	-0.987611	-1.419937	0.268078
7	C	-2.150943	-0.529781	-0.092965
8	C	-1.532106	0.691869	-0.735203
9	C	0.424814	1.956803	0.000103
10	O	0.059835	2.913612	-0.689581
11	C	1.664084	2.007519	0.843536
12	H	3.203850	-1.039080	-2.255936
13	H	3.843759	-0.701908	-0.624448

14	H	3.181986	-2.310947	-1.004092
15	H	0.004781	-0.255118	1.818152
16	H	-0.654763	-1.916173	-0.643027
17	H	-1.245482	-2.154446	1.023847
18	F	-3.060695	-1.134993	-0.890907
19	F	-2.820972	-0.163810	1.041361
20	H	-2.158423	1.572039	-0.614708
21	H	-1.362625	0.503823	-1.796127
22	H	2.493696	1.546570	0.302668
23	H	1.913121	3.048888	1.024904
24	H	1.541015	1.488644	1.793216
25	O	1.784196	-0.785031	-0.836348

7.71 *N*-acetyl-(4,4)-F_2Pro-OMe **7a**, *cis*-rotamer, *endo*-pucker, psi1-rotamer, solvent = water

SCF energy (Hartree) = -791.118763

center number	atomlabel	x	y	z
1	C	3.697801	0.245115	-0.266371
2	C	1.372570	0.399180	-0.057944
3	O	1.283593	0.816115	-1.191872
4	C	0.191945	0.182557	0.880433
5	N	-1.062266	0.533372	0.241211
6	C	-0.005338	-1.298087	1.206215
7	C	-0.800431	-1.764495	0.009659
8	C	-1.729883	-0.618879	-0.351404
9	C	-1.490036	1.787971	-0.022084
10	O	-2.508354	1.968016	-0.696650
11	C	-0.700851	2.917406	0.568502
12	H	3.641588	-0.387110	-1.149406
13	H	3.803538	1.285937	-0.563051
14	H	4.527311	-0.056010	0.363108
15	H	0.373758	0.768043	1.779660
16	H	0.919198	-1.854483	1.325733
17	H	-0.624372	-1.396020	2.095117
18	F	0.056768	-2.010335	-1.031682
19	F	-1.465639	-2.923602	0.223558
20	H	-2.713552	-0.781008	0.086921
21	H	-1.829261	-0.523098	-1.431475
22	H	-1.175458	3.855222	0.297876
23	H	-0.665347	2.825384	1.654207
24	H	0.324609	2.910585	0.199203
25	O	2.514641	0.080841	0.529112

7.72 *N*-Acetyl-(4,4)-F_2Pro-OMe **7a**, *cis*-rotamer, *endo*-pucker, psi2-rotamer, solvent = water

SCF energy (Hartree) = -791.117953

center number	atomlabel	x	y	z
1	C	-1.716000	2.418520	-1.262219
2	C	-0.635767	1.350752	0.511765

3	O	-0.561517	2.401832	1.112316
4	C	-0.076871	0.054060	1.086426
5	N	-0.183435	-1.085866	0.193757
6	C	1.434268	0.175414	1.279652
7	C	1.931863	-0.171119	-0.103785
8	C	1.011382	-1.263753	-0.621752
9	C	-1.301431	-1.802051	-0.054730
10	O	-1.298600	-2.671438	-0.931674
11	C	-2.502787	-1.512239	0.793643
12	H	-2.491106	2.834613	-0.623207
13	H	-0.915156	3.142868	-1.391950
14	H	-2.128148	2.130842	-2.222504
15	H	-0.588691	-0.118120	2.030968
16	H	1.759524	1.160969	1.599175
17	H	1.777203	-0.584930	1.977441
18	F	1.826580	0.937698	-0.905750
19	F	3.235285	-0.531216	-0.137997
20	H	1.461991	-2.242035	-0.462037
21	H	0.807581	-1.129831	-1.683245
22	H	-3.318502	-2.154555	0.477423
23	H	-2.276418	-1.699701	1.843527
24	H	-2.804251	-0.468972	0.698907
25	O	-1.192972	1.216365	-0.678276

7.73 *N*-Acetyl-(4,4)-F_2Pro-NMe_2 **7b**, *trans*-rotamer, *exo*-pucker, psi1-rotamer, solvent=octanol

SCF energy (Hartree) = -810.526208

center number	atomlabel	x	y	z
1	C	3.768606	-0.634059	-0.708438
2	N	2.543161	-0.679200	0.069996
3	C	2.691283	-0.882366	1.505314
4	C	1.382156	-0.333530	-0.505260
5	O	1.269773	-0.030621	-1.695215
6	C	0.113170	-0.388201	0.346283
7	N	-0.725310	0.769458	0.046229
8	C	-0.755559	-1.577736	-0.076828
9	C	-2.153263	-1.011269	-0.010131
10	C	-2.020953	0.416716	-0.499248
11	C	-0.230698	2.010323	0.228283
12	O	0.904325	2.161432	0.686535
13	C	-1.122172	3.156791	-0.144325
14	H	4.356589	0.248692	-0.448610
15	H	4.363597	-1.523304	-0.503153
16	H	3.526491	-0.598654	-1.765378
17	H	3.722005	-1.169094	1.698532
18	H	2.480613	0.032616	2.061289
19	H	2.050184	-1.683582	1.870195
20	H	0.330868	-0.405658	1.410748
21	H	-0.555680	-1.836720	-1.116567
22	H	-0.638964	-2.452820	0.556094
23	F	-3.059828	-1.723776	-0.718871
24	F	-2.595014	-1.010962	1.284275
25	H	-2.823332	1.041609	-0.113527
26	H	-2.031365	0.424962	-1.591574
27	H	-0.576358	4.086710	-0.017383
28	H	-1.455332	3.062853	-1.178533
29	H	-2.008901	3.166730	0.491188

7.74 *N*-Acetyl-(4,4)-F_2Pro-NMe_2 **7b**, *trans*-rotamer, *exo*-pucker, psi2-rotamer, solvent=octanol

SCF energy (Hartree) = -810.523088

center number	atomlabel	x	y	z
1	C	-3.528771	-0.880215	0.598808
2	N	-2.099694	-0.623105	0.540826
3	C	-1.531651	-0.084451	1.763330
4	C	-1.444738	-0.853579	-0.610238
5	O	-1.978583	-1.403882	-1.576251
6	C	0.029994	-0.487215	-0.759964
7	N	0.472978	0.760549	-0.141251
8	C	0.967393	-1.538875	-0.159985
9	C	2.203865	-0.726186	0.140711
10	C	1.696163	0.618000	0.628645
11	C	-0.180732	1.917717	-0.390478
12	O	-1.173496	1.926272	-1.117633

13	C	0.362004	3.150834	0.268960
14	H	-3.738197	-1.808268	1.134673
15	H	-4.014711	-0.057470	1.123433
16	H	-3.932215	-0.952076	-0.405742
17	H	-1.626683	1.003837	1.810027
18	H	-2.082276	-0.508997	2.602457
19	H	-0.490145	-0.360923	1.892209
20	H	0.171920	-0.425681	-1.839377
21	H	0.579263	-1.932005	0.779467
22	H	1.175109	-2.360866	-0.838268
23	F	3.038203	-1.316111	1.028418
24	F	2.926369	-0.548198	-1.005380
25	H	2.416838	1.404923	0.418418
26	H	1.514693	0.568446	1.705893
27	H	-0.330735	3.971436	0.109001
28	H	0.507647	2.990681	1.338150
29	H	1.330827	3.408337	-0.162379

7.75 *N*-Acetyl-(4,4)-F_2Pro-NMe_2 **7b**, *trans*-rotamer, *endo*-pucker, psi1-rotamer, solvent=octanol

SCF energy (Hartree) = -810.525836

center number	atomlabel	x	y	z
1	C	3.465856	-0.505552	1.009256
2	N	2.452159	-0.288989	-0.005985
3	C	2.976482	0.127353	-1.292497
4	C	1.152022	-0.456967	0.293457
5	O	0.759357	-0.829470	1.398602
6	C	0.127426	-0.132352	-0.807167
7	N	-1.199561	-0.512732	-0.364365
8	C	-0.026928	1.370720	-1.043976
9	C	-0.991641	1.770323	0.049910
10	C	-1.929770	0.586567	0.248562
11	C	-1.532653	-1.820717	-0.267653
12	O	-0.777233	-2.695586	-0.689763
13	C	-2.857087	-2.121879	0.367141
14	H	4.053592	-1.395669	0.776795
15	H	4.137183	0.353997	1.039453
16	H	2.995621	-0.630168	1.978552
17	H	3.379471	1.140889	-1.233081
18	H	3.786607	-0.544284	-1.580607
19	H	2.224297	0.096010	-2.073157
20	H	0.379014	-0.674001	-1.715029
21	H	0.896245	1.941380	-0.981280
22	H	-0.506359	1.545461	-2.005779
23	F	-0.304025	2.025646	1.202359
24	F	-1.667454	2.912727	-0.233873
25	H	-2.875317	0.780960	-0.257510
26	H	-2.110266	0.429163	1.312006
27	H	-3.653885	-1.526398	-0.079269
28	H	-3.076197	-3.179105	0.252109

29	H	-2.814118	-1.876073	1.429955

7.76 *N*-Acetyl-(4,4)-F_2Pro-NMe_2 **7b**, *trans*-rotamer, *endo*-pucker, psi2-rotamer, solvent=octanol

SCF energy (Hartree) = -810.520422

center number	atomlabel	x	y	z
1	C	-3.282736	-0.966329	0.684748
2	N	-1.938297	-0.438377	0.493489
3	C	-1.447504	0.376156	1.596689
4	C	-1.293199	-0.818379	-0.620584
5	O	-1.771699	-1.666745	-1.381944
6	C	0.033027	-0.221228	-1.107714
7	N	0.629392	0.890569	-0.383071
8	C	1.152405	-1.263723	-1.115846
9	C	1.847363	-0.984168	0.191870
10	C	1.839685	0.527491	0.338926
11	C	0.074564	2.123775	-0.421879
12	O	-0.952819	2.329245	-1.066842
13	C	0.759333	3.181408	0.389613
14	H	-3.260582	-1.936775	1.184741
15	H	-3.843415	-0.265287	1.300027
16	H	-3.781403	-1.079482	-0.273256
17	H	-1.742154	1.421526	1.483686
18	H	-1.896722	-0.005878	2.512513
19	H	-0.374128	0.306515	1.719744
20	H	-0.210267	0.095455	-2.121013
21	H	0.793695	-2.286549	-1.184285
22	H	1.851694	-1.058910	-1.924469
23	F	1.129335	-1.547624	1.215129
24	F	3.092887	-1.503185	0.277611
25	H	2.736314	0.940368	-0.124811
26	H	1.810486	0.814254	1.390495
27	H	1.823173	3.234498	0.157026
28	H	0.289726	4.140935	0.195734
29	H	0.664434	2.941261	1.451623

7.77 *N*-Acetyl-(4,4)-F_2Pro-NMe_2 **7b**, *cis*-rotamer, *exo*-pucker, psi1-rotamer, solvent=octanol

SCF energy (Hartree) = -810.524659

center number	atomlabel	x	y	z
1	C	3.781732	-0.602351	-0.641005
2	N	2.506909	-0.701524	0.047314
3	C	2.524725	-1.382222	1.334574
4	C	1.406184	-0.156241	-0.491650
5	O	1.380313	0.394702	-1.592378
6	C	0.110154	-0.277897	0.311423
7	N	-0.801059	0.826282	0.024921
8	C	-0.667089	-1.512337	-0.164577
9	C	-2.101568	-1.056402	-0.061691

10	C	-2.083950	0.392149	-0.496834
11	C	-0.531318	2.138456	0.204826
12	O	-1.354571	2.997861	-0.110544
13	C	0.801891	2.497657	0.796480
14	H	4.127446	-1.590896	-0.946636
15	H	3.673402	0.026373	-1.518247
16	H	4.524755	-0.165014	0.026868
17	H	2.545921	-0.677191	2.168053
18	H	1.675250	-2.052692	1.449361
19	H	3.426616	-1.988917	1.379471
20	H	0.304639	-0.329745	1.381837
21	H	-0.456698	-1.706755	-1.216704
22	H	-0.473044	-2.403937	0.425447
23	F	-2.958694	-1.813519	-0.785320
24	F	-2.520070	-1.140629	1.238238
25	H	-2.904903	0.956971	-0.062027
26	H	-2.132908	0.451775	-1.585838
27	H	1.535544	2.581835	-0.006472
28	H	0.709596	3.465578	1.281861
29	H	1.161811	1.761269	1.515218

7.78 *N*-Acetyl-(4,4)-F_2Pro-NMe_2 **7b**, *cis*-rotamer, *exo*-pucker, psi2-rotamer, solvent=octanol

SCF energy (Hartree) = -810.523071

center number	atomlabel	x	y	z
1	C	-3.447188	-1.048341	0.601343
2	N	-2.024902	-0.754369	0.543898
3	C	-1.460724	-0.273155	1.794020
4	C	-1.381067	-0.873721	-0.629386
5	O	-1.908763	-1.358788	-1.633152
6	C	0.069096	-0.413955	-0.773798
7	N	0.464483	0.809107	-0.073771
8	C	1.063459	-1.461156	-0.258946
9	C	2.263868	-0.614874	0.090487
10	C	1.701467	0.664898	0.673564
11	C	-0.159686	2.008539	-0.140006
12	O	0.260814	2.964853	0.510691
13	C	-1.378561	2.109639	-1.010880
14	H	-3.849561	-1.125241	-0.403245
15	H	-3.628567	-1.984844	1.131650
16	H	-3.953364	-0.243036	1.135354
17	H	-1.982686	-0.774867	2.608228
18	H	-0.406866	-0.511845	1.891860
19	H	-1.599044	0.804818	1.913355
20	H	0.199061	-0.286156	-1.849105
21	H	0.707075	-1.943971	0.650675
22	H	1.298851	-2.218645	-1.000436
23	F	3.131669	-1.231446	0.926576
24	F	2.965590	-0.326640	-1.046853
25	H	2.367485	1.508819	0.510341
26	H	1.533352	0.544176	1.746691

27	H	-2.254130	1.765943	-0.454874
28	H	-1.526624	3.154788	-1.268230
29	H	-1.297763	1.513896	-1.919359

7.79 *N*-Acetyl-(4,4)-F_2Pro-NMe_2 **7b**, *cis*-rotamer, *endo*-pucker, psi1-rotamer, solvent=octanol

SCF energy (Hartree) = -810.525592

center number	atomlabel	x	y	z
1	C	-3.455636	-0.389132	-0.989535
2	N	-2.416614	-0.242128	0.013253
3	C	-2.902822	0.133566	1.327503
4	C	-1.130169	-0.447079	-0.317517
5	O	-0.771805	-0.815110	-1.435819
6	C	-0.071191	-0.188556	0.769614
7	N	1.251828	-0.519906	0.276311
8	C	0.077323	1.300837	1.092416
9	C	0.990926	1.774924	-0.016089
10	C	1.931080	0.619192	-0.320801
11	C	1.732264	-1.770030	0.085656
12	O	2.808231	-1.942006	-0.485875
13	C	0.912223	-2.903795	0.629384
14	H	-3.007511	-0.480997	-1.972898
15	H	-4.061192	-1.274100	-0.784789
16	H	-4.104820	0.487106	-0.967000
17	H	-2.142787	0.026853	2.094175
18	H	-3.259531	1.165797	1.326672
19	H	-3.739397	-0.514448	1.592950
20	H	-0.304862	-0.773266	1.656612
21	H	-0.852278	1.863830	1.101469
22	H	0.595335	1.421811	2.042212
23	F	0.246256	2.083607	-1.120551
24	F	1.658714	2.911744	0.305167
25	H	2.903727	0.789974	0.138886
26	H	2.059309	0.497897	-1.396402
27	H	1.436006	-3.835777	0.439417
28	H	0.756995	-2.786151	1.702561
29	H	- 0.065610	-2.935094	0.147401

7.80 *N*-Acetyl-(4,4)-F_2Pro-NMe_2 **7b**, *cis*-rotamer, *endo*-pucker, psi2-rotamer, solvent=octanol

SCF energy (Hartree) = -810.520818

center number	atomlabel	x	y	z
1	C	3.150376	-1.332975	-0.652660
2	N	1.872385	-0.652232	-0.491046
3	C	1.444027	0.105701	-1.660374
4	C	1.198836	-0.867030	0.650733
5	O	1.587449	-1.683369	1.492347
6	C	-0.051008	-0.079909	1.076962
7	N	-0.563720	0.991769	0.236727

8	C	-1.264101	-1.003147	1.211677
9	C	-1.927310	-0.839434	-0.132598
10	C	-1.772400	0.627567	-0.487621
11	C	-0.052919	2.239753	0.127790
12	O	-0.582247	3.063046	-0.617660
13	C	1.160603	2.552182	0.954278
14	H	3.606527	-1.508947	0.316267
15	H	3.023604	-2.290155	-1.162333
16	H	3.804931	-0.701104	-1.250689
17	H	0.371122	0.079680	-1.801780
18	H	1.786042	1.142444	-1.622963
19	H	1.891847	-0.364362	-2.534656
20	H	0.233004	0.309089	2.054111
21	H	-1.003741	-2.035636	1.424781
22	H	-1.940176	-0.621060	1.974451
23	F	-1.267345	-1.609799	-1.056323
24	F	-3.217092	-1.245950	-0.159491
25	H	-2.634797	1.192950	-0.134226
26	H	-1.673638	0.770936	-1.564014
27	H	1.529413	3.534591	0.675214
28	H	0.900572	2.553094	2.014110
29	H	1.946329	1.809791	0.807677

7.81 *N*-Acetyl-(4,4)-F_2Pro-NMe_2 **7b**, *trans*-rotamer, *exo*-pucker, psi1-rotamer, solvent = water

SCF energy (Hartree) = -810.525569

center number	atomlabel	x	y	z
1	C	3.844400	-0.340923	-0.628771
2	N	2.577215	-0.552529	0.050480
3	C	2.644137	-1.282924	1.310498
4	C	1.441222	-0.073449	-0.468195
5	O	1.373404	0.491015	-1.567034
6	C	0.161581	-0.304942	0.331223
7	N	-0.800648	0.762140	0.078753
8	C	-0.554953	-1.562531	-0.173890
9	C	-2.010303	-1.172930	-0.088781
10	C	-2.052288	0.288628	-0.484252
11	C	-0.471991	2.042822	0.330962
12	O	0.629073	2.318836	0.823309
13	C	-1.498418	3.081061	-0.002696
14	H	4.567745	0.065159	0.077698
15	H	4.232833	-1.281855	-1.019425
16	H	3.711421	0.359056	-1.446103
17	H	3.570222	-1.852813	1.315893
18	H	2.647296	-0.610279	2.168839
19	H	1.823781	-1.990165	1.407613
20	H	0.370961	-0.356569	1.397854
21	H	-0.321095	-1.733075	-1.224914
22	H	-0.333164	-2.453219	0.406901
23	F	-2.820013	-1.941697	-0.854179

24	F	-2.455384	-1.313619	1.196513
25	H	-2.917332	0.789070	-0.056622
26	H	-2.077029	0.365825	-1.572786
27	H	-1.072016	4.067193	0.151441
28	H	-1.828100	2.975527	-1.036306
29	H	-2.373046	2.959030	0.637500

7.82 *N*-Acetyl-(4,4)-F_2Pro-NMe_2 **7b**, *trans*-rotamer, *exo*-pucker, psi2-rotamer, solvent = water

SCF energy (Hartree) = -810.523433

center number	atomlabel	x	y	z
1	C	-3.478599	-0.952083	0.607283
2	N	-2.043494	-0.694064	0.589983
3	C	-1.460293	-0.081963	1.773889
4	C	-1.427656	-0.859223	-0.589354
5	O	-2.021437	-1.350578	-1.559825
6	C	0.044953	-0.507233	-0.776683
7	N	0.478378	0.770645	-0.212065
8	C	0.998980	-1.523497	-0.142801
9	C	2.223906	-0.684004	0.129934
10	C	1.701137	0.670285	0.567659
11	C	-0.246701	1.891653	-0.398198
12	O	-1.266657	1.866483	-1.096364
13	C	0.243632	3.127027	0.291120
14	H	-3.689798	-1.993074	0.368160
15	H	-3.859028	-0.731318	1.599989
16	H	-3.992868	-0.323648	-0.120285
17	H	-1.562311	1.006015	1.765189
18	H	-1.983128	-0.466458	2.646249
19	H	-0.413240	-0.342679	1.887707
20	H	0.176317	-0.490704	-1.858475
21	H	0.620142	-1.891061	0.809878
22	H	1.219646	-2.362885	-0.794571
23	F	3.063467	-1.231808	1.038980
24	F	2.948718	-0.541472	-1.020991
25	H	2.411751	1.458360	0.330682
26	H	1.513104	0.658849	1.643854
27	H	-0.474533	3.927497	0.145061
28	H	0.377909	2.936588	1.357045
29	H	1.210788	3.424968	-0.115374

7.83 *N*-Acetyl-(4,4)-F_2Pro-NMe_2 **7b**, *trans*-rotamer, *endo*-pucker, psi1-rotamer, solvent = water

SCF energy (Hartree) = -810.526507

center number	atomlabel	x	y	z
1	C	3.437572	-0.438335	1.028703
2	N	2.471693	-0.233470	-0.041021
3	C	2.981520	0.365194	-1.263918
4	C	1.176499	-0.397616	0.258740

5	O	0.810433	-0.783802	1.375326
6	C	0.141032	-0.083453	-0.827541
7	N	-1.167692	-0.559711	-0.414918
8	C	-0.091319	1.418433	-1.001923
9	C	-1.111228	1.718643	0.071829
10	C	-1.988397	0.481691	0.190280
11	C	-1.394131	-1.884919	-0.287534
12	O	-0.557991	-2.707778	-0.675976
13	C	-2.696122	-2.282803	0.335237
14	H	4.435794	-0.417441	0.599941
15	H	3.359578	0.342748	1.786183
16	H	3.275661	-1.400635	1.508753
17	H	3.295543	1.395418	-1.087436
18	H	3.844450	-0.203619	-1.606160
19	H	2.239618	0.358912	-2.054960
20	H	0.434296	-0.562244	-1.757685
21	H	0.797567	2.032778	-0.884071
22	H	-0.547797	1.609405	-1.971109
23	F	-0.477890	1.960920	1.259685
24	F	-1.837964	2.834667	-0.186876
25	H	-2.917385	0.638029	-0.356425
26	H	-2.211870	0.280922	1.237207
27	H	-3.528693	-1.736332	-0.106590
28	H	-2.842645	-3.351058	0.210550
29	H	-2.671528	-2.043277	1.400073

7.84 *N*-Acetyl-(4,4)-F_2Pro-NMe_2 **7b**, *trans*-rotamer, *endo*-pucker, psi2-rotamer, solvent = water

SCF energy (Hartree) = -810.520242

center number	atomlabel	x	y	z
1	C	-3.255021	-1.006192	0.704558
2	N	-1.919618	-0.450487	0.506816
3	C	-1.422812	0.376844	1.601133
4	C	-1.292489	-0.804124	-0.621739
5	O	-1.793084	-1.638862	-1.393287
6	C	0.033611	-0.219179	-1.119722
7	N	0.636128	0.899418	-0.411492
8	C	1.150154	-1.265101	-1.116245
9	C	1.847485	-0.975824	0.188146
10	C	1.835931	0.535464	0.331612
11	C	0.061820	2.120504	-0.421062
12	O	-0.974722	2.321979	-1.062943
13	C	0.729274	3.172546	0.406916
14	H	-3.207474	-2.039154	1.051434
15	H	-3.769544	-0.405432	1.449519
16	H	-3.816880	-0.979431	-0.225177
17	H	-1.725496	1.418596	1.485050
18	H	-1.858675	-0.002524	2.522894
19	H	-0.348197	0.316681	1.711989
20	H	-0.207546	0.081707	-2.137854
21	H	0.791054	-2.288254	-1.173556

22	H	1.848440	-1.067289	-1.926706
23	F	1.137047	-1.542723	1.215584
24	F	3.095194	-1.490867	0.270128
25	H	2.737458	0.949353	-0.119347
26	H	1.789177	0.827342	1.380522
27	H	1.792326	3.239493	0.176242
28	H	0.247935	4.129405	0.232135
29	H	0.636418	2.904727	1.462429

7.85 *N*-Acetyl-(4,4)-F_2Pro-NMe_2 **7b**, *cis*-rotamer, *exo*-pucker, psi1-rotamer, solvent = water

SCF energy (Hartree) = -810.524351

center number	atomlabel	x	y	z
1	C	3.767780	-0.649134	-0.663541
2	N	2.497025	-0.713461	0.039821
3	C	2.524431	-1.340886	1.357010
4	C	1.398219	-0.165741	-0.489444
5	O	1.361336	0.374776	-1.601541
6	C	0.110018	-0.279318	0.323262
7	N	-0.799437	0.831142	0.045974
8	C	-0.673455	-1.510417	-0.151305
9	C	-2.105984	-1.048572	-0.056593
10	C	-2.081271	0.401842	-0.484946
11	C	-0.507456	2.138081	0.199116
12	O	-1.316969	3.007841	-0.143780
13	C	0.822135	2.489823	0.798273
14	H	4.120321	-1.653876	-0.895460
15	H	3.649763	-0.087868	-1.583466
16	H	4.509851	-0.157983	-0.034319
17	H	2.476630	-0.600549	2.156667
18	H	1.715111	-2.057983	1.478751
19	H	3.462351	-1.882414	1.446542
20	H	0.311843	-0.334310	1.390798
21	H	-0.461565	-1.709835	-1.201859
22	H	-0.482743	-2.398798	0.443834
23	F	-2.960725	-1.796408	-0.793610
24	F	-2.539712	-1.139451	1.237528
25	H	-2.904321	0.965775	-0.053740
26	H	-2.123431	0.463203	-1.573716
27	H	1.562500	2.566672	0.000925
28	H	0.733686	3.459794	1.279617
29	H	1.169375	1.752135	1.521166

7.86 *N*-Acetyl-(4,4)-F_2Pro-NMe_2 **7b**, *cis*-rotamer, *exo*-pucker, psi2-rotamer, solvent = water

SCF energy (Hartree) = -810.522236

center number	atomlabel	x	y	z

1	C	-3.418167	-1.098343	0.572904
2	N	-1.983225	-0.839005	0.580898
3	C	-1.425581	-0.289819	1.808196
4	C	-1.377797	-0.854227	-0.615598
5	O	-1.968507	-1.245908	-1.630943
6	C	0.074414	-0.414302	-0.782164
7	N	0.476787	0.822513	-0.105541
8	C	1.070100	-1.452765	-0.254426
9	C	2.273615	-0.603436	0.073639
10	C	1.718426	0.687169	0.637667
11	C	-0.188153	1.997081	-0.124333
12	O	0.223074	2.956147	0.537279
13	C	-1.431068	2.079652	-0.959686
14	H	-3.943240	-0.335376	-0.005462
15	H	-3.633171	-2.069785	0.133578
16	H	-3.779418	-1.081544	1.596659
17	H	-1.904980	-0.785559	2.648781
18	H	-0.360308	-0.477200	1.887462
19	H	-1.610547	0.784139	1.891694
20	H	0.196644	-0.301861	-1.859480
21	H	0.719175	-1.916319	0.666509
22	H	1.300255	-2.222907	-0.983841
23	F	3.144429	-1.206662	0.916064
24	F	2.973113	-0.337576	-1.071461
25	H	2.387669	1.524555	0.456738
26	H	1.553434	0.583283	1.712341
27	H	-2.276282	1.669968	-0.400624
28	H	-1.634841	3.126040	-1.166879
29	H	-1.338478	1.528196	-1.894291

7.87 *N*-Acetyl-(4,4)-F_2Pro-NMe_2 **7b**, *cis*-rotamer, *endo*-pucker, psi1-rotamer, solvent = water

SCF energy (Hartree) = -810.526144

center number	atomlabel	x	y	z
1	C	3.435437	0.349904	-1.008807
2	N	2.402993	0.212476	0.004751
3	C	2.899160	-0.212904	1.302837
4	C	1.116915	0.423559	-0.303802
5	O	0.741437	0.800734	-1.420133
6	C	0.072804	0.170785	0.796570
7	N	-1.251877	0.538989	0.327007
8	C	-0.102167	-1.319874	1.093616
9	C	-1.027578	-1.756473	-0.019135
10	C	-1.940269	-0.575380	-0.310178
11	C	-1.661661	1.805456	0.093748
12	O	-2.707944	2.017444	-0.527883
13	C	-0.819758	2.911547	0.655631
14	H	2.983217	0.477744	-1.985630
15	H	4.070278	1.208697	-0.788089
16	H	4.056152	-0.546126	-1.011438
17	H	2.162848	-0.082394	2.087835

18	H	3.204563	-1.259996	1.272764
19	H	3.771216	0.389750	1.555952
20	H	0.332444	0.733657	1.689425
21	H	0.817308	-1.898463	1.091947
22	H	-0.619460	-1.445283	2.042577
23	F	-0.293150	-2.069094	-1.131568
24	F	-1.722926	-2.881177	0.284819
25	H	-2.924039	-0.740694	0.125110
26	H	-2.044284	-0.426137	-1.384700
27	H	-1.308358	3.860175	0.456591
28	H	-0.689716	2.783930	1.730334
29	H	0.169463	2.912180	0.196995

7.88 *N*-Acetyl-(4,4)-F_2Pro-NMe_2 **7b**, *cis*-rotamer, *endo*-pucker, psi2-rotamer, solvent = water

SCF energy (Hartree) = -810.519523

center number	atomlabel	x	y	z
1	C	3.150695	-1.323958	-0.650005
2	N	1.871825	-0.633397	-0.507653
3	C	1.433965	0.144162	-1.662807
4	C	1.221549	-0.822019	0.647079
5	O	1.645770	-1.618234	1.499243
6	C	-0.042689	-0.065103	1.080454
7	N	-0.590663	0.990935	0.241522
8	C	-1.230927	-1.019661	1.221323
9	C	-1.897197	-0.884327	-0.124355
10	C	-1.777619	0.581420	-0.496238
11	C	-0.105690	2.244515	0.117368
12	O	-0.647573	3.046009	-0.650497
13	C	1.084031	2.603857	0.955517
14	H	3.721365	-1.254830	0.272341
15	H	3.006986	-2.378126	-0.889970
16	H	3.709781	-0.849933	-1.451586
17	H	0.363848	0.082964	-1.816878
18	H	1.738220	1.189933	-1.591104
19	H	1.906955	-0.284643	-2.542878
20	H	0.234400	0.334257	2.054811
21	H	-0.945948	-2.043874	1.441315
22	H	-1.915863	-0.647305	1.980236
23	F	-1.221454	-1.650523	-1.040669
24	F	-3.177373	-1.321076	-0.146936
25	H	-2.660772	1.124819	-0.162126
26	H	-1.666468	0.712599	-1.572656
27	H	1.423513	3.596374	0.676994
28	H	0.807636	2.596143	2.010735
29	H	1.895368	1.887732	0.822135

8 Compound synthesis and characterisation

8.1 α,α-Deuterated octanol

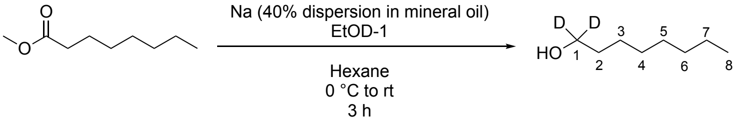

To a solution of methyl octanoate (31.6 g, 1 equiv) in hexane (1 L) was added EtOD-d_1 (52.4 mL 4.5 equiv), followed by a Na dispersion in oil (40% wt., 28.8 g, 4.5 equiv) at 0 °C. After 5 min, the reaction is allowed to warm to rt and then after a further 5 minutes, the reaction is quenched with aq. HCl (3M, 450 mL). The mixture was diluted with Et_2O (200 mL) and brine (200 mL) and the aqueous layer was extracted with Et_2O (2 x 800 mL) before the combined organic layers were dried over $MgSO_4$. The solution was then filtered over a plug of silica gel. The crude was purified by flash column chromatography (3:7, Et_2O/pentane) to afford a light orange oil which was then further purified by distillation (64 – 66 °C, 8 mbar) to yield a colourless oil (15.3 g, 58%).

MW: 132.24 g mol^{-1} ($C_8H_{16}D_2O$); **R$_f$** 0.023 (5:95 Et_2O/hexane); **^{1}H NMR** (400 MHz, $CDCl_3$) δ 3.62 (residual signal, 2%, H-1), 1.56 (2H, t, *J* 7.34 Hz, H-2), 1.46–1.29 (10H, m, H-3/4/5/6/7), 0.89 (3H, t, *J* 7.34 Hz, H-8) ppm; **^{13}C{^{1}H} NMR** (101 MHz, $CDCl_3$) δ 62.3 (quin, *J* 21.9 Hz, C-1), 32.6 (C-2), 31.8 (C-4/5/6/7), 29.4 (C-4/5/6/7), 29.3 (C-4/5/6/7), 25.7 (C-3), 22.6 (C-4/5/6/7), 14.1 (C-8) ppm.
Data consistent with literature.[5]

8.2 Known compounds

Synthesis of *N*-(Acetyl)-(2S,4R)-4-fluoroproline methyl ester **9a** has been previously reported by Hudlicky,[6] Raines [7] and Wennemers.[8] The synthesis of *N*-(Acetyl)-(2*S*,4*R*)-4-fluoroproline dimethyl amide **9b** has been previously reported by Wennemers.[8] Our data is consistent with the literature. The synthesis of *N*-(acetyl)-(2*S*)-4,4-difluoroproline methyl ester **10a** has been previously reported by Raines.[9] Our data are consistent with the literature.

8.3 *N*-Acetyl-3-fluoropyrrolidine (**2**)

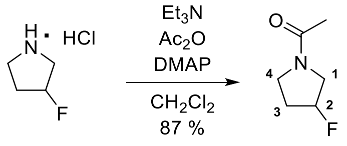

To a solution of 3-fluoropyrrolidine hydrochloride (315 mg, 1 equiv) in CH_2Cl_2 (10 mL) was added Et_3N (0.75 mL, 2.2 equiv) and DMAP (15 mg, 0.05 equiv). Once all the 3-fluoropyrrolidine hydrochloride had dissolved, acetic anhydride (0.28 mL, 1.2 equiv) was added dropwise. After 6 h the reaction was quenched with sat. aq. NH_4Cl (10 mL) and washed with CH_2Cl_2 (10 mL). The organic layer was washed with aq. 2 M HCl (2×10 mL), sat. aq. $NaHCO_3$ (2×10 mL) and brine, and dried over $MgSO_4$ and concentrated *in vacuo*. The crude oil was purified by column chromatography (3:7, acetone/petrol ether 40–60 °C) to afford a pale-yellow oil (272 mg, 87%).

Rotamer ratio: 1:0.73 ($CDCl_3$), Cis Major; **^{1}H NMR** (400MHz, $CDCl_3$) δ (mixture of rotamers) 5.33–5.15 (m, 1H, major, H2), 5.29 (td, *J*=3.7, 52.6 Hz, 1H, minor, H2), 3.96–3.31 (m, 4H, major + minor, H1 + H4), 2.40–1.85 (m, 2H, major + minor, H3), 2.08 (s, 3H, major, CH_3), 2.05 (s, 3H, minor, CH_3) ppm; **^{13}C{^{1}H} NMR** (101MHz, $CDCl_3$) δ (mixture of rotamers) 169.5 (major, C=O), 169.3 (minor, C=O), 92.9 (d, *J*=176.8 Hz, minor, C2), 91.6 (d, *J*=176.1 Hz, major, C2), 53.8 (d, *J*=23.5 Hz, minor C1), 52.2 (d, *J*=23.5 Hz, major, C1), 45.0 (major, C4), 43.3 (minor, C4), 32.8 (d, *J*=22.0 Hz, major, C3), 31.2 (d, *J*=21.3 Hz, minor, C3), 22.4 (minor, CH_3), 22.3 (major, CH_3) ppm; **^{19}F NMR** (376MHz, $CDCl_3$) δ -177.2– -177.7 (m, 1F, minor), -177.7– -178.4 (m, 1F, major) ppm; **^{19}F{^{1}H} NMR** (376MHz, $CDCl_3$) δ -177.5 (s, 1F, minor), -178.0 (s, 1F, major) ppm; **IR** (neat) 2982 (w), 2886 (w), 1623 (s), 1420 (m), 1204 (s), 1093 (s) cm^{-1}; **MS** (ESI+) m/z 132.1 [M+H]$^+$, 154.2 [M+Na]$^+$; **HRMS** (ESI+) $C_6H_{11}FNO$ [M+H]$^+$, calculated 132.0819 found 132.0818 (+1.0 ppm error), $C_6H_{11}FNNaO$ [M+Na]$^+$, calculated 154.0639, found 154.0636 (+1.8 ppm error.)

8.4 *N*-Acetyl-3,3-difluoropyrrolidine (**3**)

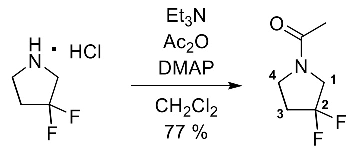

SI3 **3**

To a solution of 3,3-difluoropyrrolidine hydrochloride (305 mg, 1 equiv) in CH_2Cl_2 (10 mL) was added Et_3N (0.65 mL, 2.2 equiv) and DMAP (20 mg, 0.05 equiv). Once all the 3,3-difluoropyrrolidine hydrochloride had dissolved, acetic anhydride (0.24 mL, 1.2 equiv) was added dropwise. After 6 h the reaction was quenched with sat. aq. NH_4Cl (10 mL) and washed with CH_2Cl_2 (10 mL). The organic layer is then washed with aq. 2 M HCl (2×10 mL), sat. aq. $NaHCO_3$ (2×10 mL) and brine, and dried over $MgSO_4$ and concentrated *in vacuo*. The crude oil was then purified by column chromatography (3:7, acetone/petrol ether 40–60 °C) to afford a pale-yellow oil (243 mg, 77%).

Rotamer ratio 1:0.81 ($CDCl_3$), Cis Major; **^{1}H NMR** (400MHz, $CDCl_3$) δ (mixture of rotamers) 3.80 (t, *J*=13.1 Hz, 2H, major, H1), 3.79 (t, *J*=12.6 Hz, 2H, minor, H1), 3.73–3.64 (m, 2H, major + minor, H4), 2.57–2.26 (m, 2H, major + minor, H3), 2.06 (s, 3H, major, CH_3), 2.04 (s, 3H, minor, CH_3) ppm; **^{13}C{^{1}H} NMR** (101MHz, $CDCl_3$) δ (mixture of rotamers) 169.4 (major, C=O), 169.2 (minor, C=O), 126.5 (t, *J*=247.2 Hz, minor, C2), 127.5 (t, *J*=249.4 Hz, major, C2), 54.0 (t, *J*=31.9 Hz, minor, C3), 52.2 (t, *J*=31.9 Hz, major, C3), 44.8 (t, *J*=3.3 Hz, major, C4), 42.9 (t, *J*=2.9 Hz minor, C4), 34.5 (t, *J*=24.6 Hz, major, C1), 33.1 (t, *J*=23.5 Hz, minor, C1), 22.2 (major, CH_3), 21.2 (minor, CH_3) ppm; **^{19}F NMR** (376MHz, $CDCl_3$) δ -101.2 (quin, *J*=13.0 Hz, 2F, minor), -102.2 (quin, *J*=13.0 Hz, 2F, major) ppm; **^{19}F{^{1}H} NMR** (376MHz, $CDCl_3$) δ -101.2 (s, 2F, minor), -102.2 (s, 2F, major) ppm; **IR** (neat) 2963 (w), 2866 (w), 1626 (s), 1422(m), 1213 (s), 1118 (s) cm^{-1}; **MS** (ESI+) m/z 150.2 $[M+H]^+$, 172.2 $[M+Na]^+$; **HRMS** (ESI+) $C_6H_{10}F_2NO$ $[M+H]^+$, calculated 150.0725, found 150.0725 (+0.2 ppm error).

8.5 *N*-Acetyl-3-fluoropiperidine (**4**)

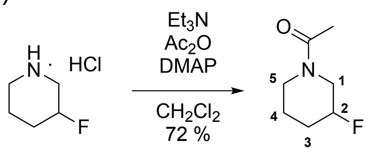

SI4 **4**

To a solution of 3-fluoropiperidine hydrochloride of (296 mg, 1 equiv) in CH_2Cl_2 (10 mL) was added Et_3N (0.9 mL, 3.1 equiv) and DMAP (17 mg, 0.05 equiv). Once all the 3-fluoropiperidine hydrochloride had dissolved, acetic anhydride (0.33 mL, 1.2 equiv) was added dropwise. After 6 h the reaction was quenched with sat. aq. NH_4Cl (10 mL) and washed with CH_2Cl_2 (10 mL). The organic layer is then washed with aq. 2 M HCl (2×10 mL), sat. aq. $NaHCO_3$ (2×10 mL), and brine, and dried over $MgSO_4$ and concentrated *in vacuo*. The crude oil was then purified by column chromatography (3:7, acetone/petrol ether 40–60 °C) to afford a pale-yellow oil (223 mg, 72%).

Rotamer ratio 1:0.8 ($CDCl_3$), undetermined (acetate overlap); **^{1}H NMR** (400MHz, $CDCl_3$) δ (mixture of rotamers) 4.83–4.48 (m, 1H, major + minor, H2), 4.11–4.01 (m, 1H, major, H'5), 3.90 (ddd, *J*=13.8, 8.8, 6.2 Hz, 1H, minor, H'1), 3.75 (ddd, *J*=14.2, 9.0, 5.1 Hz, 1H, major, H'1), 3.65 (ddd, *J*=24.7, 13.7, 3.3 Hz, 1H, minor, H"1), 3.54–3.40 (m, 2H, major, H"1 and major, H'5), 3.40–3.29 (m, 1H, major H"5), 3.11 (tt, *J*=8.9, 4.0 Hz, 1H, major, H"5), 2.11 (s, 3H, major + minor, CH_3), 2.05–1.73 (m, 3H, major + minor, H3 and major + minor H'4), 1.63–1.44 (m, 1H, major + minor, H"4) ppm; **^{13}C{^{1}H} NMR** (101MHz, $CDCl_3$) δ (mixture of rotamers) 169.7 (major, C=O), 169.7 (minor, C=O), 86.4 (d, *J*=175.3 Hz, major, C2), 86.1 (d, *J*=174.6 Hz, minor, C2), 50.6 (d, *J*=22.7 Hz, major, C1), 46.2 (minor, C5), 45.4 (d, *J*=24.9 Hz, minor, C1), 41.7 (major, C5), 29.6 (d, *J*=20.5 Hz, minor, C3), 29.5 (d, *J*=21.3 Hz, major, C3), 21.8 (d, *J*=4.4 Hz, minor C4), 21.4 (major + minor, CH_3), 20.5 (d, *J*=2.9 Hz, major, C4) ppm; **^{19}F NMR** (376MHz, $CDCl_3$) δ -184.20– -184.61 (m, 1F, minor), -185.8 (tddd, *J*=45.8, 36.7, 28.0, 9.8 Hz, 1F, major) ppm; **^{19}F{^{1}H} NMR** (376MHz, $CDCl_3$) δ -184.4 (s, 1F, minor), -185.8 (s, 1F, major) ppm; **IR** (neat) 2947 (w), 2864 (w), 1625 (s), 1427 (m), 1200 (s), 1123 (s) cm^{-1}; **MS** (ESI+) m/z 146.2 $[M+H]^+$, 168.2 $[M+Na]^+$; **HRMS** (ESI+) $C_7H_{12}FNO$ $[M+H]^+$, calculated 146.0976,

found 146.0973 (+1.9 ppm error). $C_7H_{13}FNNaO$ $[M+Na]^+$, calculated 168.0795, found 168.0793 (+1.0 ppm error.)

8.6 *N*-Acetyl-3,3-difluoropiperidine (**5**)

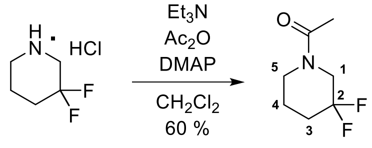

SI5 **5**

To a solution of 3,3-difluoropiperidine hydrochloride (312 mg, 1 equiv) in CH_2Cl_2 (10 mL) was added Et_3N (0.58 mL, 2.2 equiv) and DMAP (10 mg, 0.05 equiv). Once all the 3,3-difluoropiperidine hydrochloride had dissolved, acetic anhydride (0.22 mL, 1.2 equiv) was added dropwise. After 6 h the reaction was quenched with sat. aq. NH_4Cl (10 mL) and washed with CH_2Cl_2 (10 mL). The organic layer is then washed with aq. 2 M HCl (2×10 mL), sat. aq. $NaHCO_3$ (2×10 mL), and brine, and dried over $MgSO_4$ and concentrated *in vacuo*. The crude oil was then purified by column chromatography (30% acetone/petrol ether 40–60 °C) to afford a pale-yellow oil (187 mg, 60%).

Rotamer ratio 1:0.72 ($CDCl_3$), Cis Major; **^{1}H NMR** (400MHz, $CDCl_3$) δ (mixture of rotamers) 3.86–3.36 (m, 4H, major + minor, H1 + H5), 2.12 (s, 3H, CH_3) + 2.11 (s, 3H, major, CH_3), 2.09–1.97 (m, 2H, major + minor, H3), 1.84–1.66 (m, 2H, major + minor, H4) ppm; **^{13}C{^{1}H} NMR** (101MHz, $CDCl_3$) δ (mixture of rotamers) 169.6 (major, C=O), 169.4 (minor, C=O), 119.1 (t, *J*=244.7 Hz, C2), 52.2 (t, *J*=31.5 Hz, major, C1), 46.9 (t, *J*=32.6 Hz, minor, C1), 45.5 (minor, C5), 41.0 (major, C5), 32.6 (t, *J*=23.8 Hz, minor, C3), 32.5 (t, *J*=23.1 Hz, major, C3), 22.6 (t, *J*=4.4 Hz, minor, C4), 21.7 (t, *J*=4.4 Hz, major, C4), 21.4 (minor, CH_3), 21.3 (major, CH_3) ppm; **^{19}F NMR** (376MHz, $CDCl_3$) δ -103.3 (quin, *J*=12.6 Hz, 2F, minor), -103.8 (quin, *J*=12.6 Hz, 2F, major) ppm; **^{19}F{^{1}H} NMR** (376MHz, $CDCl_3$) δ -103.3 (s, 2F, minor), -103.8 (s, 2F, major) ppm; **IR** (neat) 2941 (w), 2869 (w), 1641 (s), 1427 (m), 1243 (s), 1103 (s) cm^{-1}; **MS** (ESI+) m/z 164.2 $[M+H]^+$, 186.2 $[M+Na]^+$; **HRMS** (ESI+) $C_7H_{12}F_2NO$ $[M+H]^+$, calculated 164.0885, found: 164.0881 (-2.0 ppm error), $C_7H_{13}FNNaO$ $[M+Na]^+$, calculated 186.0705, found 186.0701 (-2.4 ppm error.)

8.7 *N*-acetyl-(2*S*)-4,4-difluoroproline dimethyl amide (**10b**)

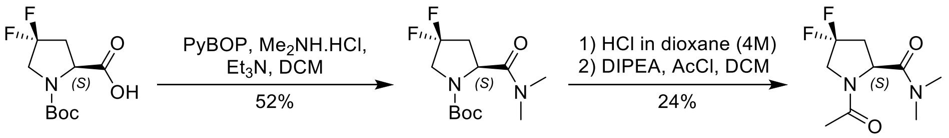

S1 **S2** **10b**

To a solution of **S1** (537.5 mg, 2.14 mmol), which is commercially available but synthesised according to literature procedures,[10] in DCM (18.0 mL) at 0 °C was added triethylamine (0.68 mL, 4.88 mmol), PyBOP (1.22 g, 2.35 mmol) and $HNMe_2$.HCl (174.4 mg, 2.14 mmol). The reaction mixture was allowed to warm to rt and stirred overnight. After 18 hours, the solvent was removed *in vacuo* to yield an orange oil which was redissolved in ethyl acetate (25 mL). The organic phase was washed with a saturated aqueous solution of $NaHCO_3$ (3 × 20 mL), a 10% aqueous solution of HCl (2 × 10 mL) and brine (20 mL). Next, the organic phase was dried over $MgSO_4$ and the solvent was removed *in vacuo*. Purification by column chromatography using a Biotage® purification system (hexane/acetone gradient) gave **S2** as a white solid (307.2 mg, 52%). Intermediate **S2** was then dissolved in a 4 M solution of HCl in dioxane (3.0 mL) and stirred at rt. After one hour, the solvent was removed *in vacuo* to give an off-white solid which was redissolved in DCM (5.0 mL). Next, DIPEA (0.22 mL, 1.24 mmol) and acetyl chloride (0.08 mL, 1.08 mmol) were added. After 16 hours, the solvent was removed *in vacuo* and the resulting oil was redissolved in DCM (20 mL) and water (20 mL). The aqueous phase was extracted with DCM (3 × 20 mL) and the combined organic phases were dried over $MgSO_4$ and evaporated *in vacuo*. Purification by flash chromatography (ethyl acetate/acetonitrile/methanol 50:25:25) followed by HPLC (DCM/methanol 95:5) yielded **10b** (44.1 mg, 24 %) as a clear oil.

^{1}H NMR (400 MHz, $CDCl_3$): (90:10 rotamer ratio) δ 4.99 (br t, *J*=7.8 Hz, 1H, major $C_\alpha H$), 4.82 (dd, *J*=9.2, 5.5 Hz, 1H, minor $C_\alpha H$), 4.22–3.76 (m, 2H major $C_\delta H_2$ + 2H minor $C_\delta H_2$), 3.13 (s, 3H, major $NC\underline{H}_3$), 3.06 (s, 3H,

minor NC<u>H</u>$_3$), 3.01 (s, 3H, minor NC'<u>H'</u>$_3$), 2.96 (s, 3H, major NC'<u>H'</u>$_3$), 2.70–2.32 (m, 2H minor $C_{\beta}H_2$ + 2H major $C_{\beta}H_2$), 2.06 (s, 3H, major NCOC<u>H</u>$_3$), 1.89 (s, 3H, minor NCOC<u>H</u>$_3$) ppm; **^{13}C{^{1}H} NMR** (100 MHz, $CDCl_3$): mixture of rotamers δ 170.2 (major C_{α}-<u>C</u>O), 169.6 (minor N<u>C</u>OCH$_3$), 169.3 (minor C_{α}-<u>C</u>O), 169.1 (major N<u>C</u>OCH$_3$), 126.5 (dd, *J*=252.4, 247.2 Hz, major C_{γ}), (minor C_{γ} not visible), 56.2 (d, *J*=2.9 Hz, minor C_{α}), 54.6 (dd, *J*=33.8, 30.1 Hz, major C_{δ}), 53.7 (d, *J*=4.4 Hz, major C_{α}), 53.1 (dd, *J*=33.0, 30.8 Hz, minor C_{δ}), 39.3 (dd, *J*=27.1, 24.9 Hz, minor C_{β}), 37.5 (dd, *J*=26.0, 23.8 Hz, major C_{β}), 36.9 (major N<u>C</u>H$_3$), 36.7 (minor N<u>C</u>H$_3$), 36.2 (minor N<u>C'</u>H$_3$), 35.9 (major N<u>C'</u>H$_3$), 22.1 (major NCO<u>C</u>H$_3$), 20.8 (minor NCO<u>C</u>H$_3$) ppm; **^{19}F NMR** (471 MHz, $CDCl_3$): (90:10 rotamer ratio) δ -94.9 (dm, *J*= 231.9 Hz, 1F, major F), -96.7 (dm, *J*= 233.3 Hz, 1F, minor F), -101.2 (dm, *J*= 232.6 Hz, 1F, minor F'), -102.7 (dm, *J*= 231.2 Hz, 1F, major F') ppm; **^{19}F (^{1}H} NMR** (471 MHz, $CDCl_3$): (90:10 rotamer ratio) δ -94.9 (d, *J*=231.0 Hz, 1F, major F), -96.7 (d, *J*=233.2 Hz, 1F, minor F), -101.2 (d, *J*=233.2 Hz, 1F, minor F'), -102.7 (d, *J*=231.0 Hz, 1F, major F') ppm; **Rf** 0.38 (hexane/acetone 60:40); **$(\alpha)_D$** –13.3 (c 0.5, $CHCl_3$, 22 °C); **MS** (ESI)(*m/z*): 221.3 $[M+H]^+$, 243.3 $[M+Na]^+$; **HRMS** (ESI) for $C_9H_{14}F_2N_2NaO_2$ $[M+Na]^+$ Calcd. 243.0916; Found 243.0912; **IR** 1641 (s), 1417 (m), 1370 (m), 1141 (m), 1094 (m) cm^{-1}.

9 NMR spectra of novel compounds

9.1 *N*-Acetyl-3-fluoropyrrolidine (**2**): ^{1}H NMR spectrum ($CDCl_3$, 400 MHz)

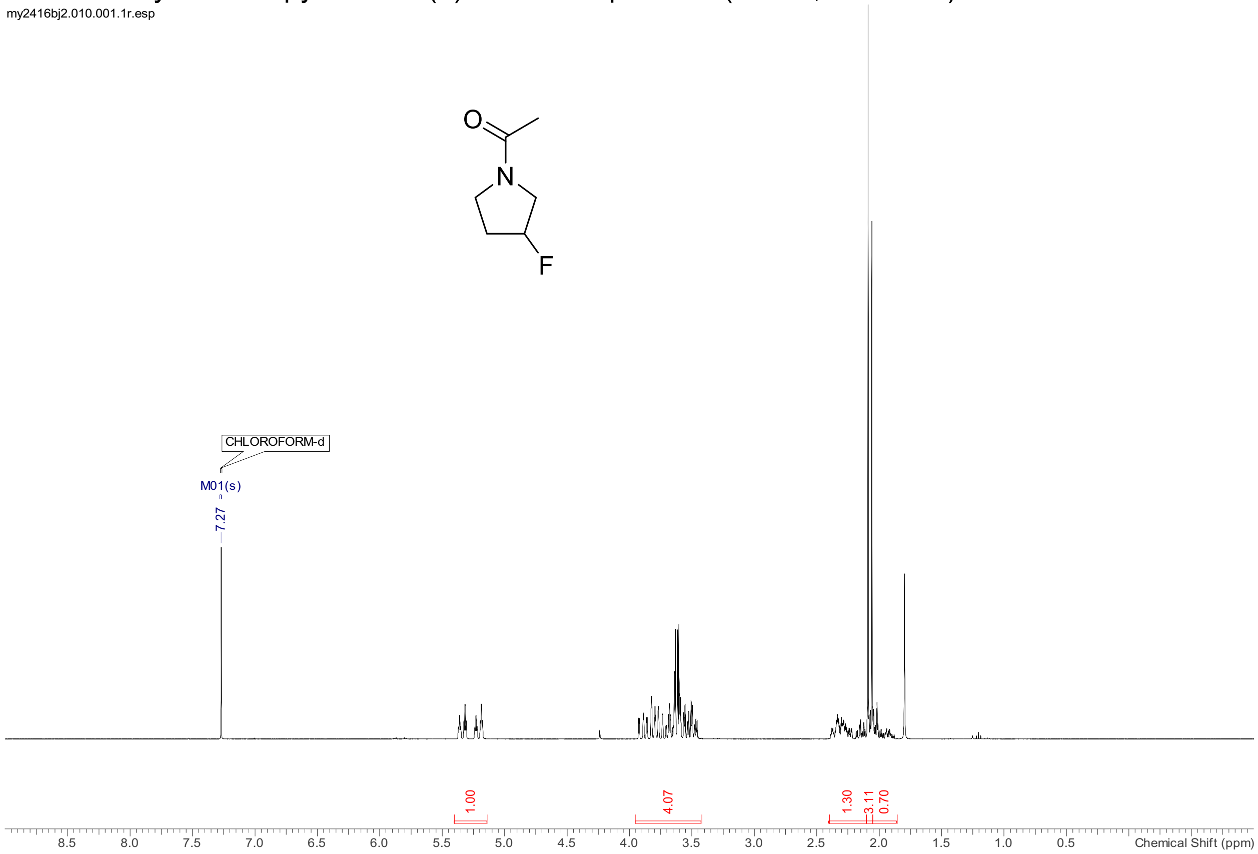

9.2 *N*-Acetyl-3-fluoropyrrolidine (**2**): ^{13}C{^{1}H} NMR spectrum ($CDCl_3$, 101 MHz)

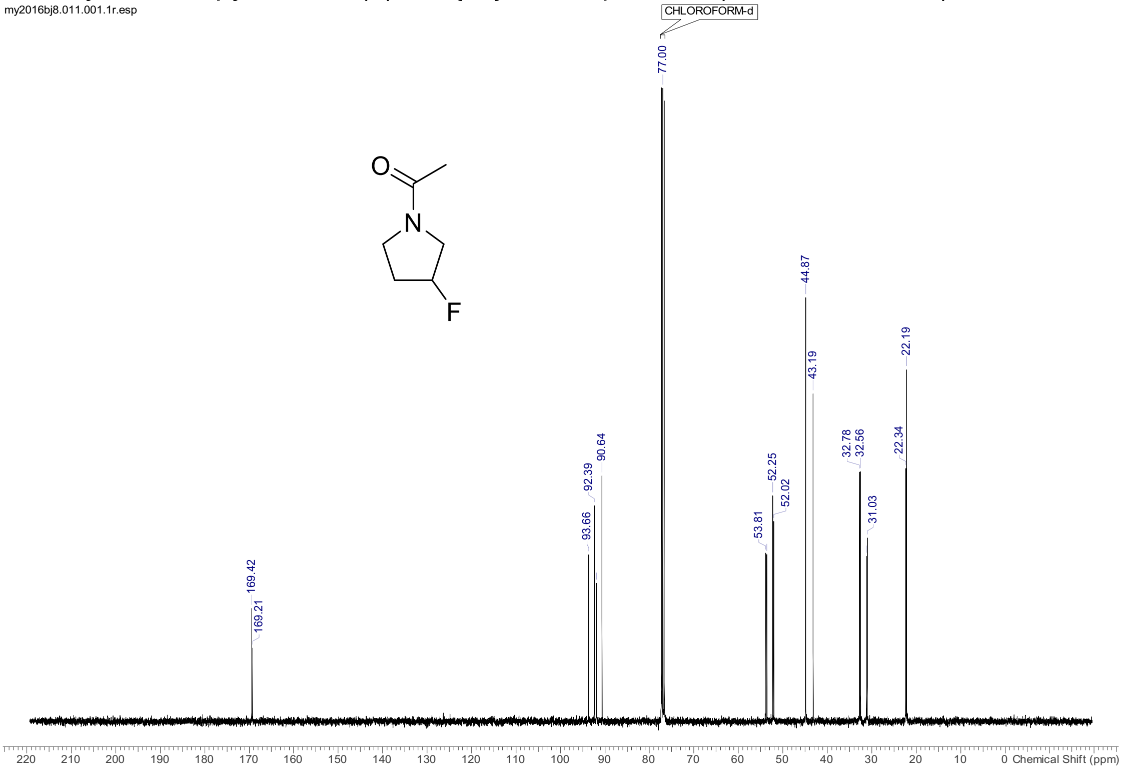

9.3 *N*-Acetyl-3-fluoropyrrolidine (**2**): ^{19}F NMR spectrum ($CDCl_3$, 376 MHz)

my2416bj2.011.001.1r.esp

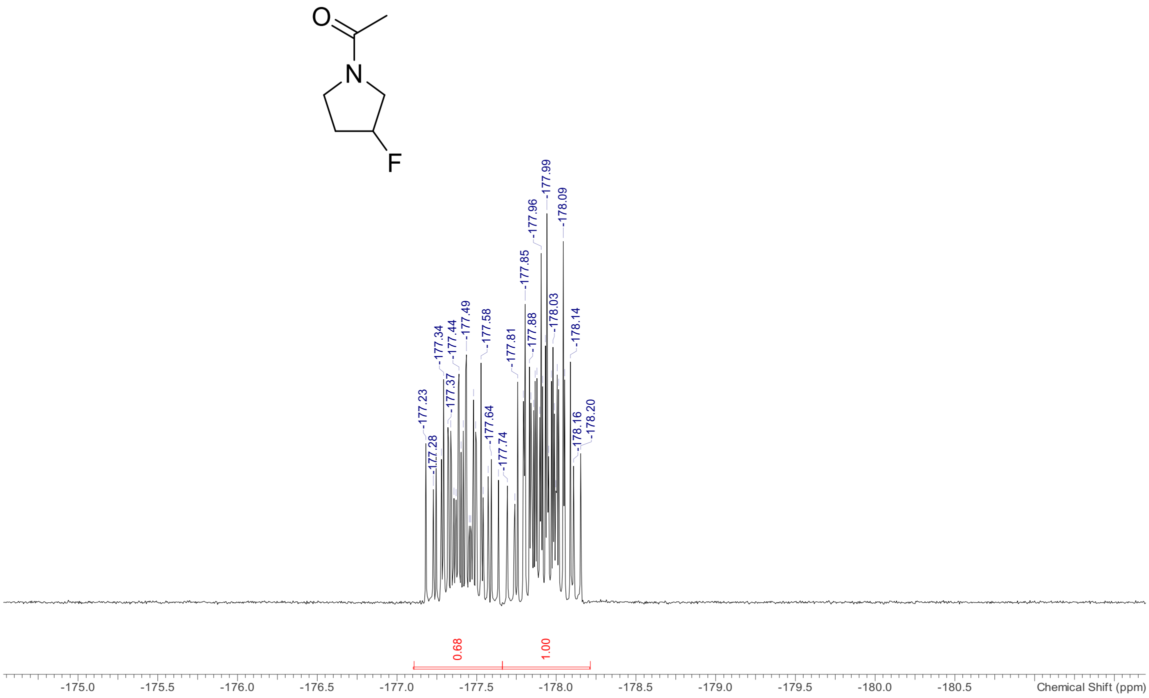

9.4 *N*-Acetyl-3-fluoropyrrolidine (**2**): ^{19}F {H} NMR spectrum ($CDCl_3$, 376 MHz)

my2416bj2.012.001.1r.esp

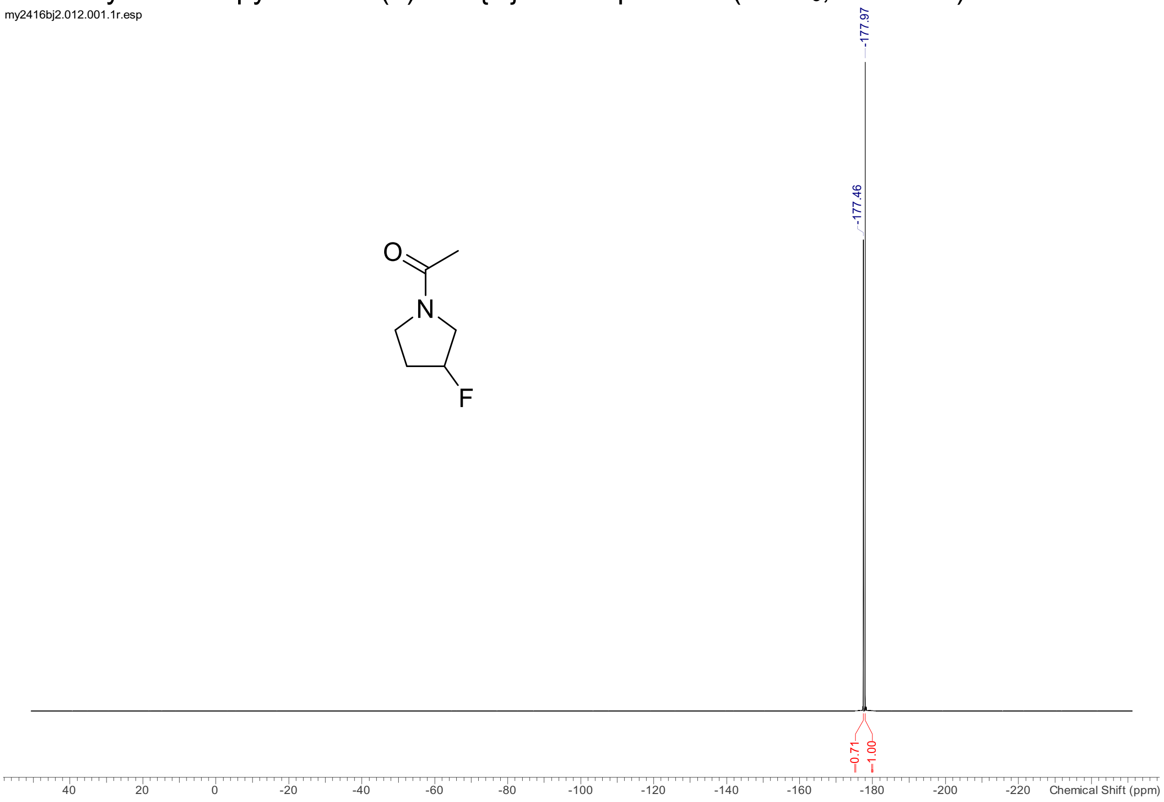

9.5 *N*-Acetyl-3,3-difluoropyrrolidine (**3**): ^{1}H NMR spectrum ($CDCl_3$, 400 MHz)

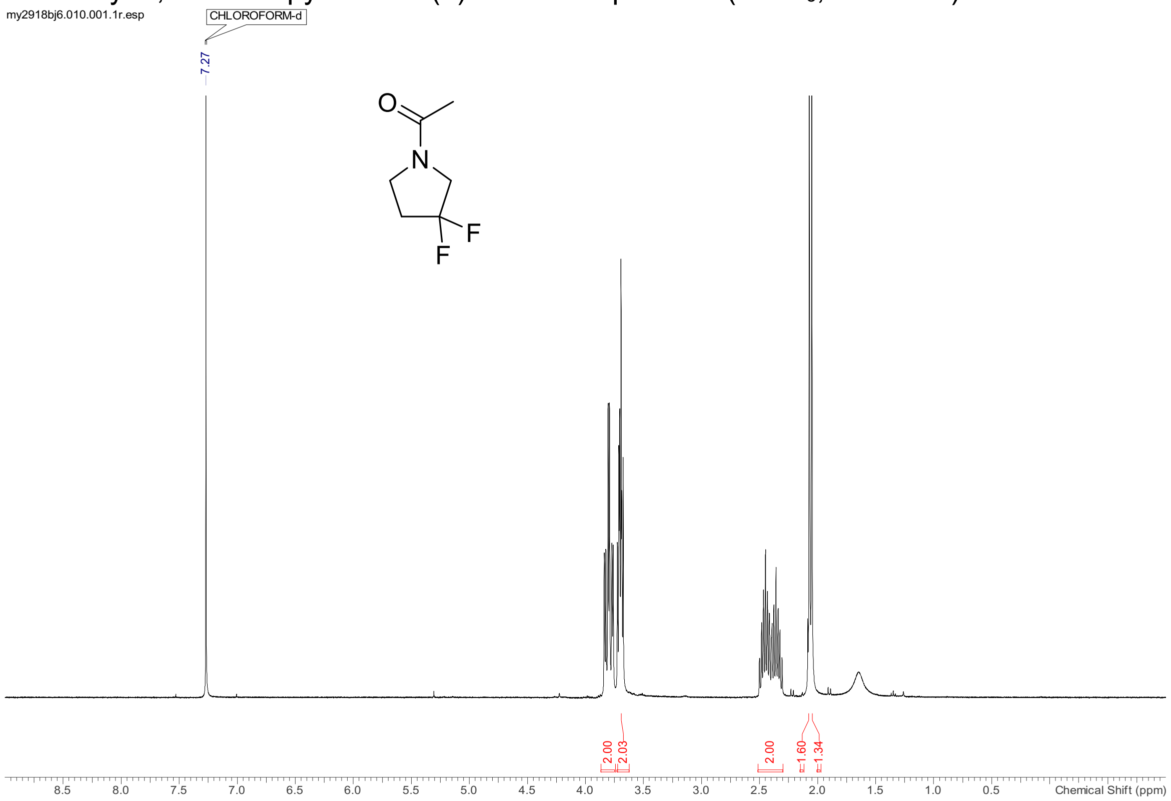

9.6 *N*-Acetyl-3,3-difluoropyrrolidine (**3**): $^{13}C\{^{1}H\}$ NMR spectrum ($CDCl_3$, 101 MHz)

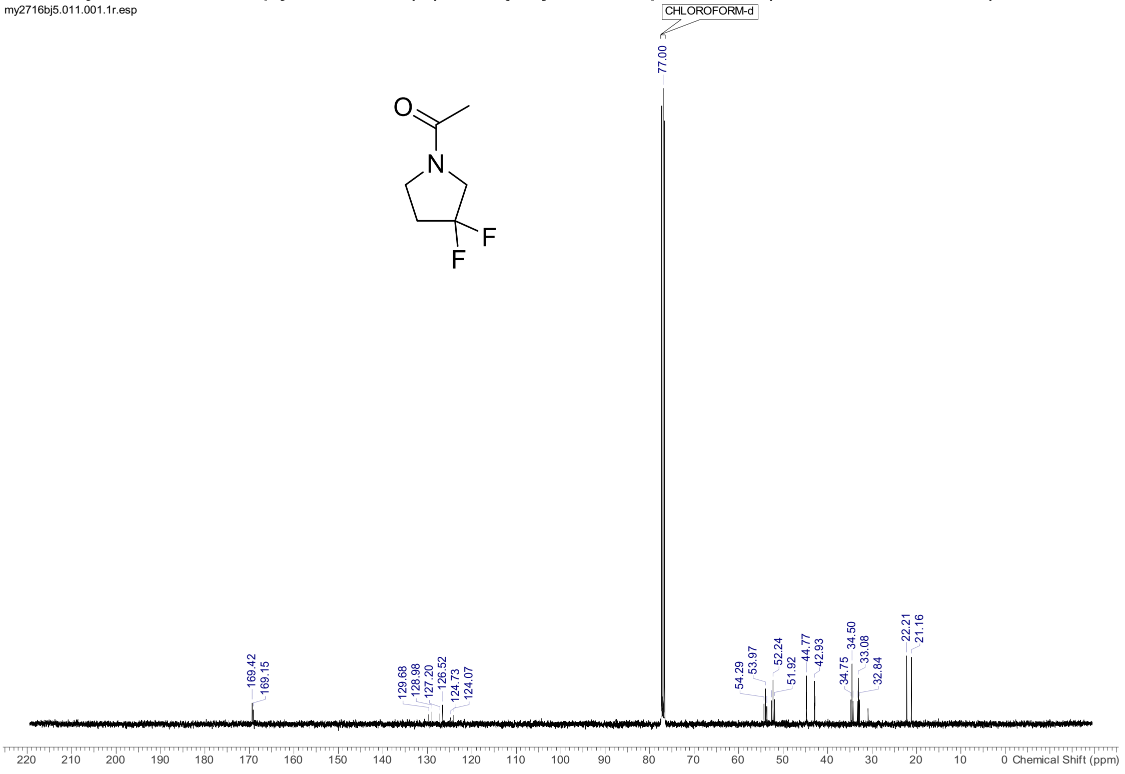

9.7 *N*-Acetyl-3,3-difluoropyrrolidine (**3**): ^{19}F NMR spectrum ($CDCl_3$, 376 MHz)

my2918bj6.011.001.1r.esp

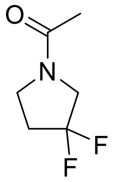

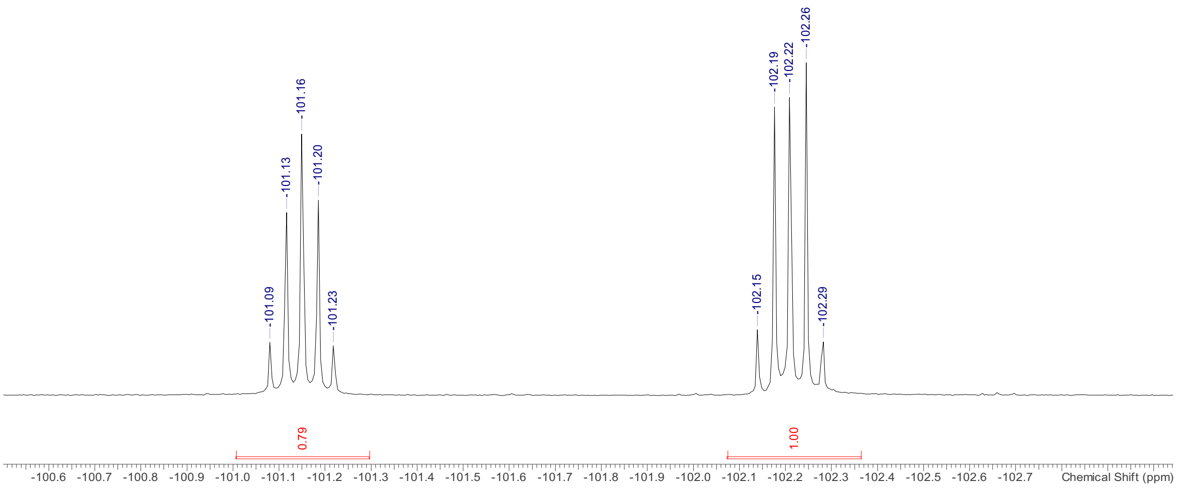

9.8 *N*-Acetyl-3,3-difluoropyrrolidine (**3**): ^{19}F ^{1}\{H\} NMR spectrum ($CDCl_3$, 376 MHz)

my2918bj6.012.001.1r.esp

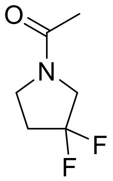

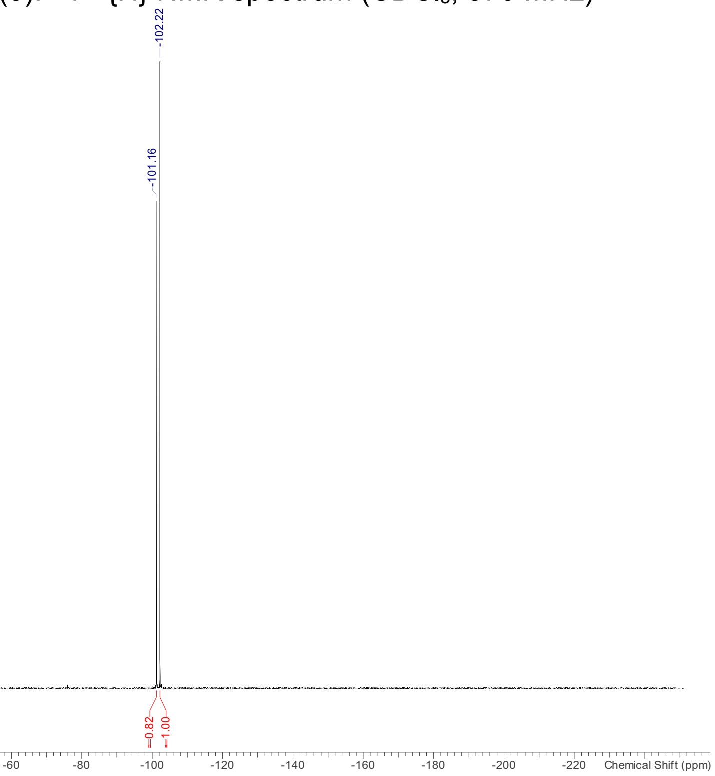

9.9 *N*-Acetyl-3-fluoropiperidine (**4**): ^{1}H NMR spectrum ($CDCl_3$, 400 MHz)

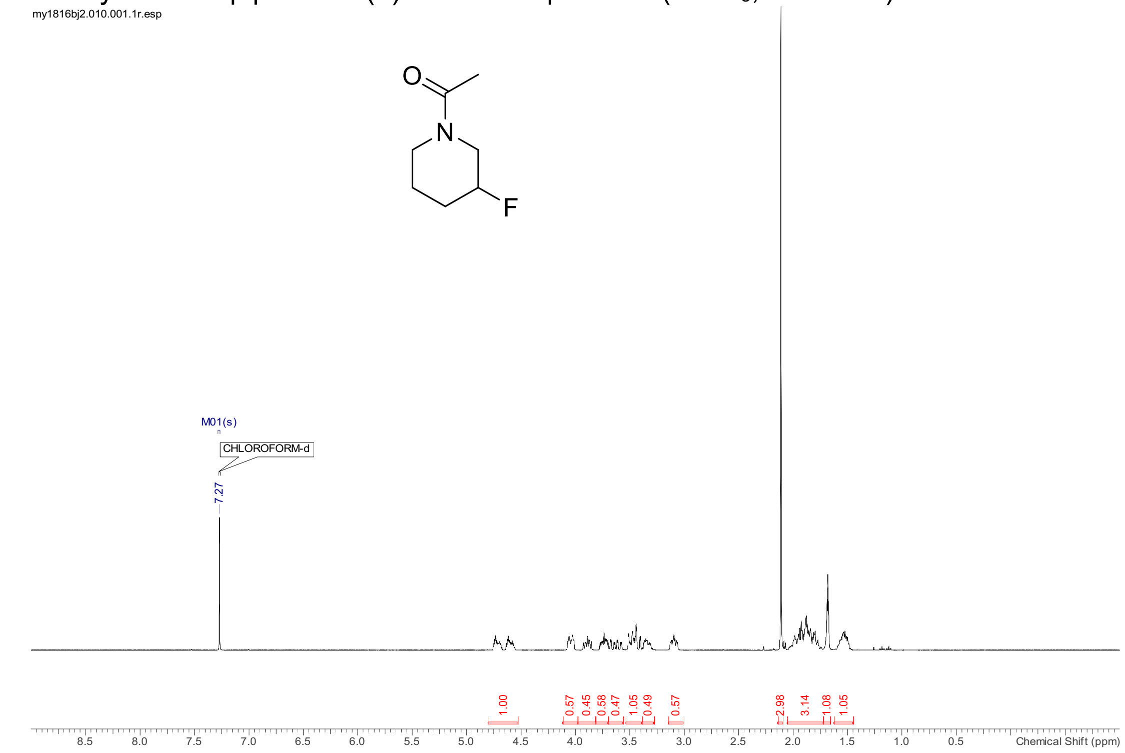

9.10 *N*-Acetyl-3-fluoropiperidine (**4**): ^{13}C{^{1}H} NMR spectrum ($CDCl_3$, 101 MHz)

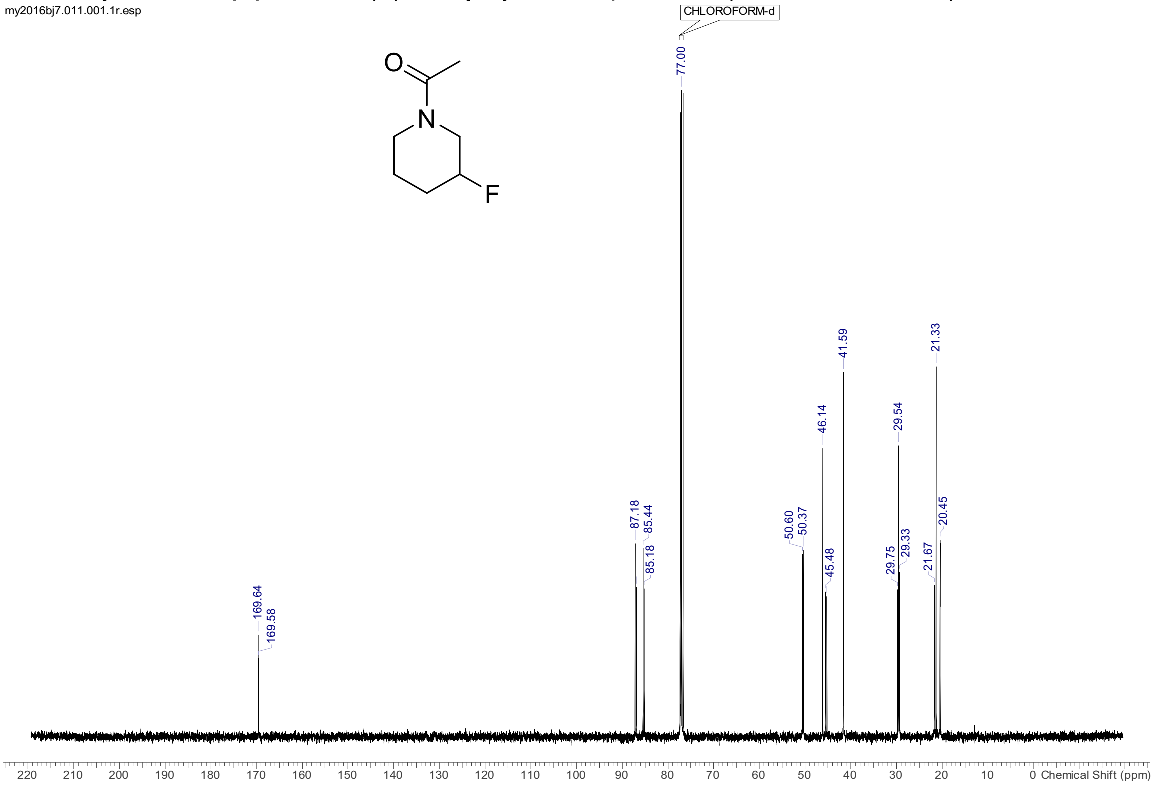

9.11 *N*-Acetyl-3-fluoropiperidine (**4**): ^{19}F NMR spectrum ($CDCl_3$, 376 MHz)

my1816bj2.011.001.1r.esp

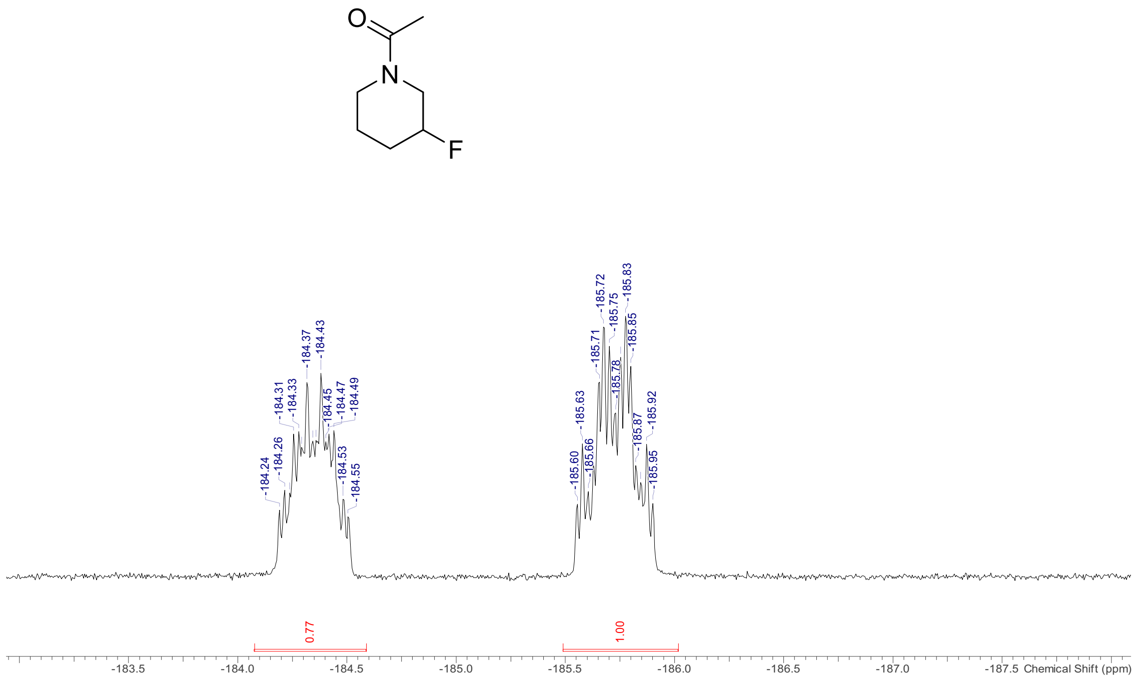

9.12 *N*-acetyl-3-fluoropiperidine (**4**): ^{19}F ^{1}{H} NMR spectrum ($CDCl_3$, 376 MHz)

my1816bj2.012.001.1r.esp

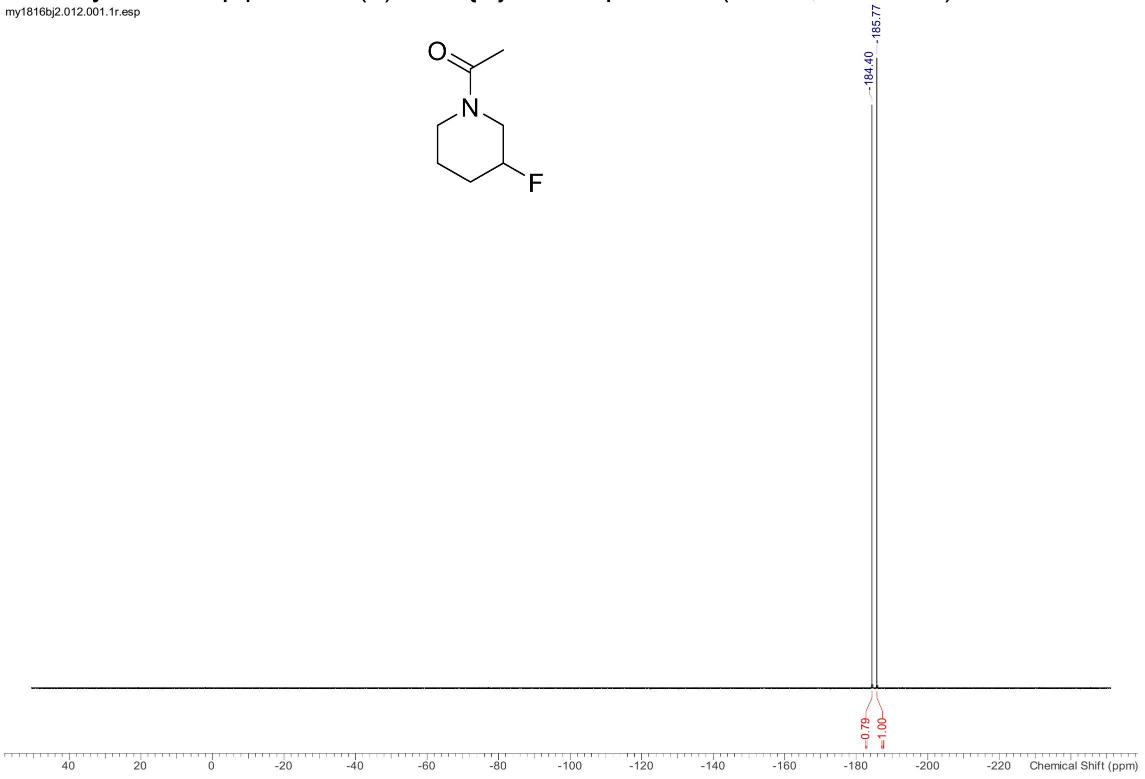

9.13 *N*-Acetyl-3,3-difluoropyrrolidine (**5**): ^{1}H NMR spectrum ($CDCl_3$, 400 MHz)

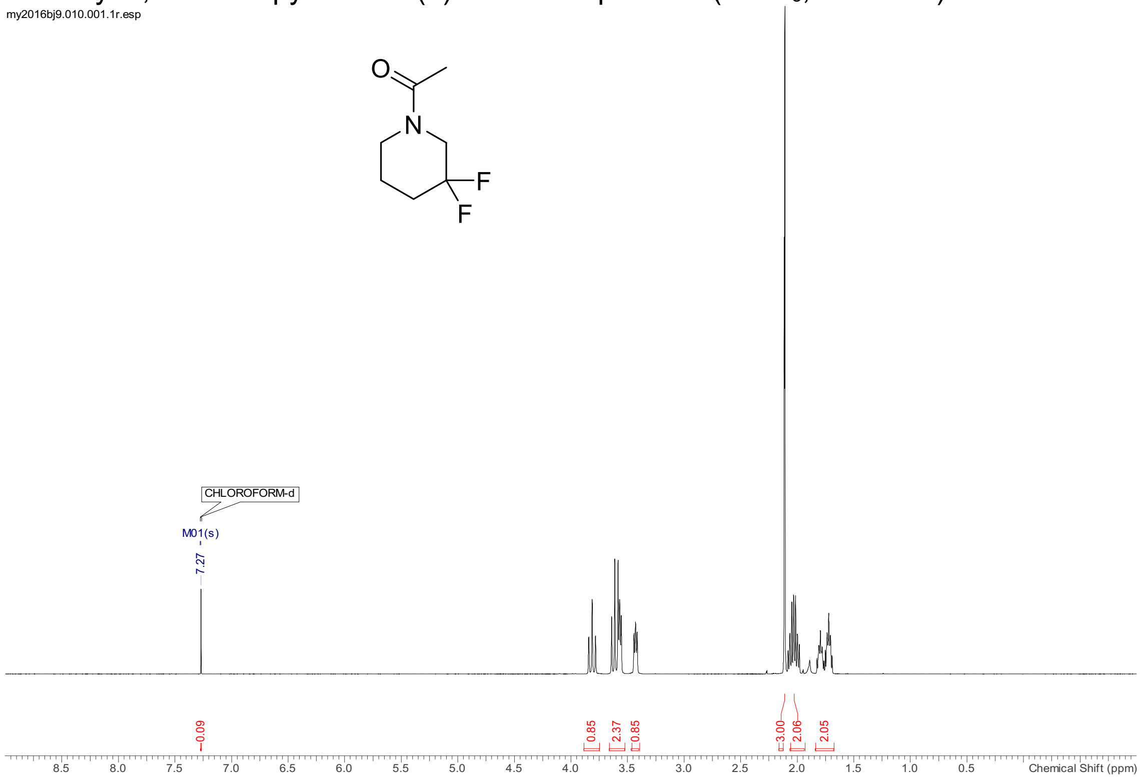

9.14 *N*-Acetyl-3,3-difluoropyrrolidine (**5**): ^{13}C{^{1}H} NMR spectrum ($CDCl_3$, 101 MHz)

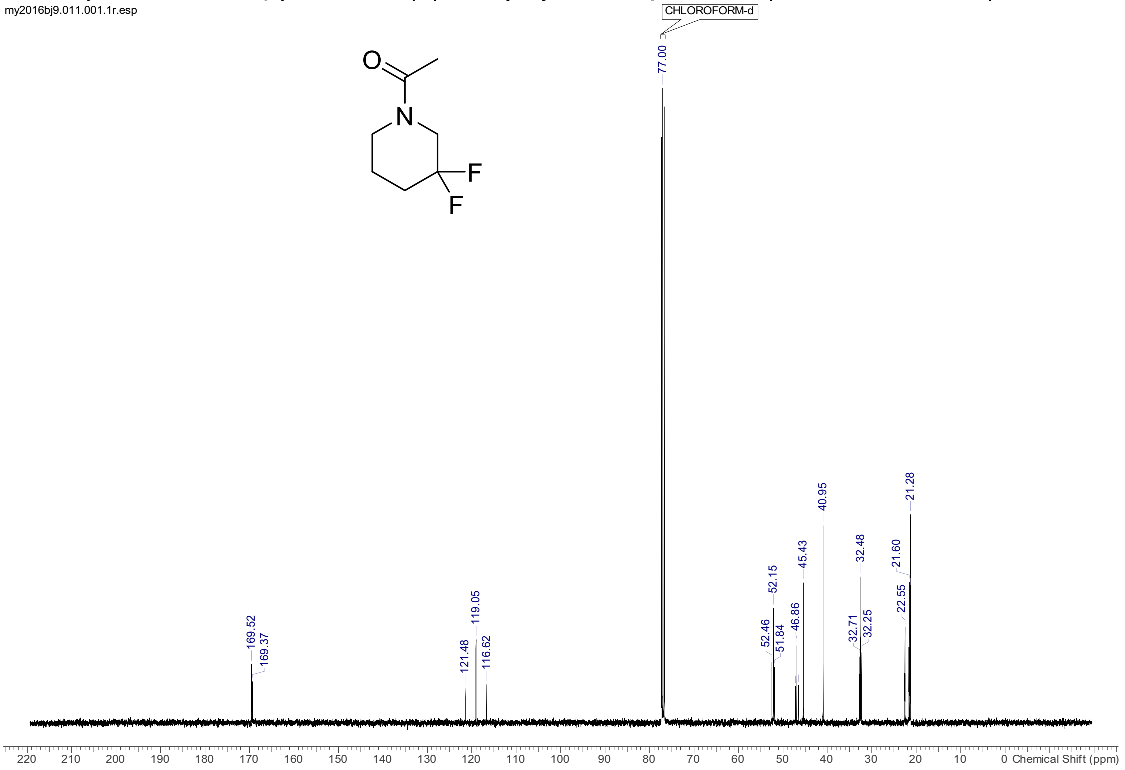

9.15 *N*-Acetyl-3,3-difluoropyrrolidine (**5**):^{19}F NMR spectrum ($CDCl_3$, 376 MHz)

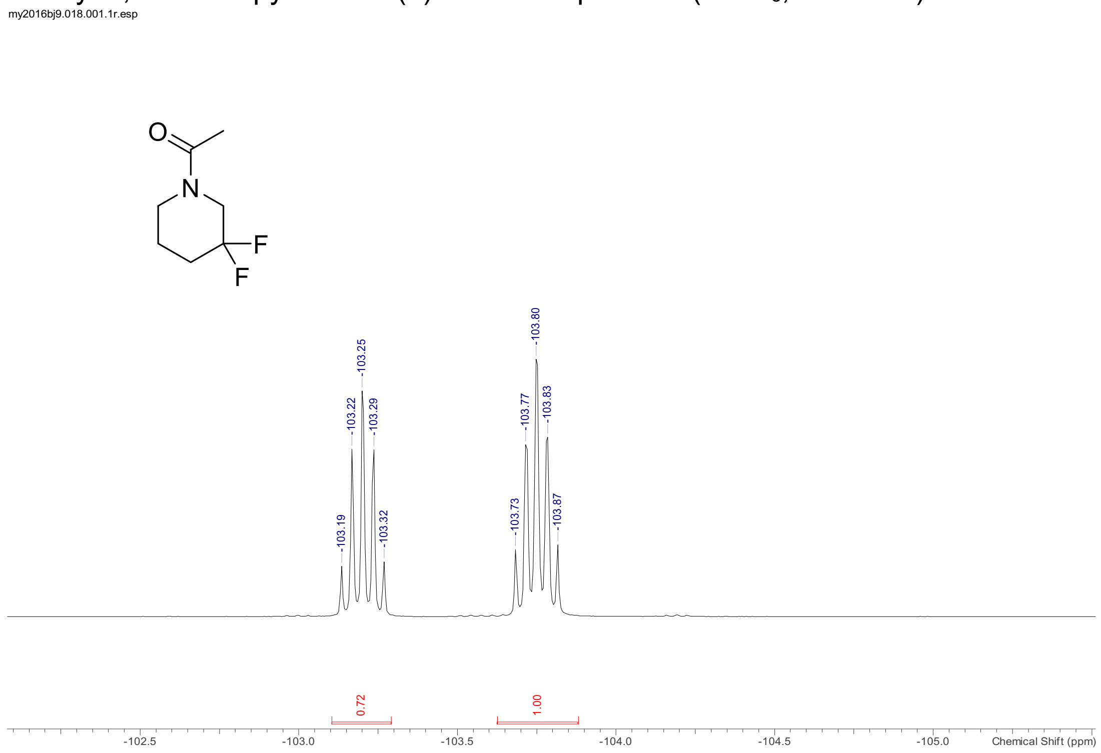

9.16 *N*-Acetyl-3,3-difluoropyrrolidine (**5**):^{19}F {H} NMR spectrum ($CDCl_3$, 376 MHz)

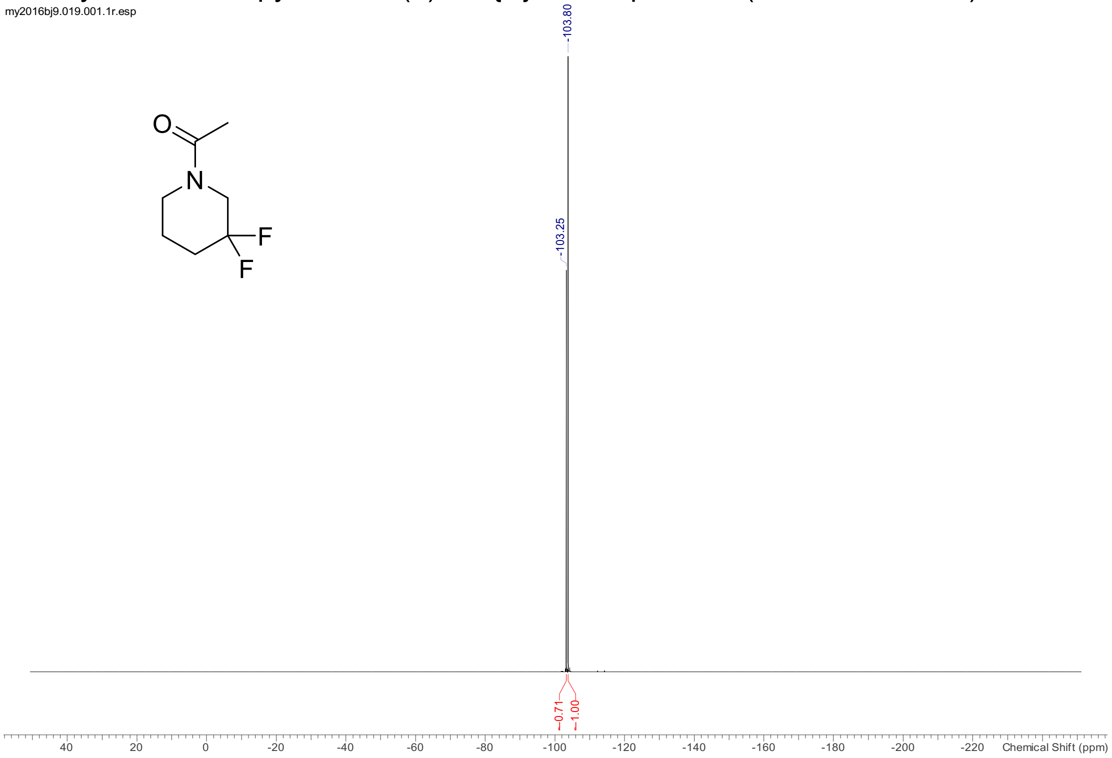

9.17 *N*-(Acetyl)-(2*S*)-4,4-difluoroproline dimethyl amide **7b**: ^{1}H NMR spectrum ($CDCl_3$, 400 MHz)

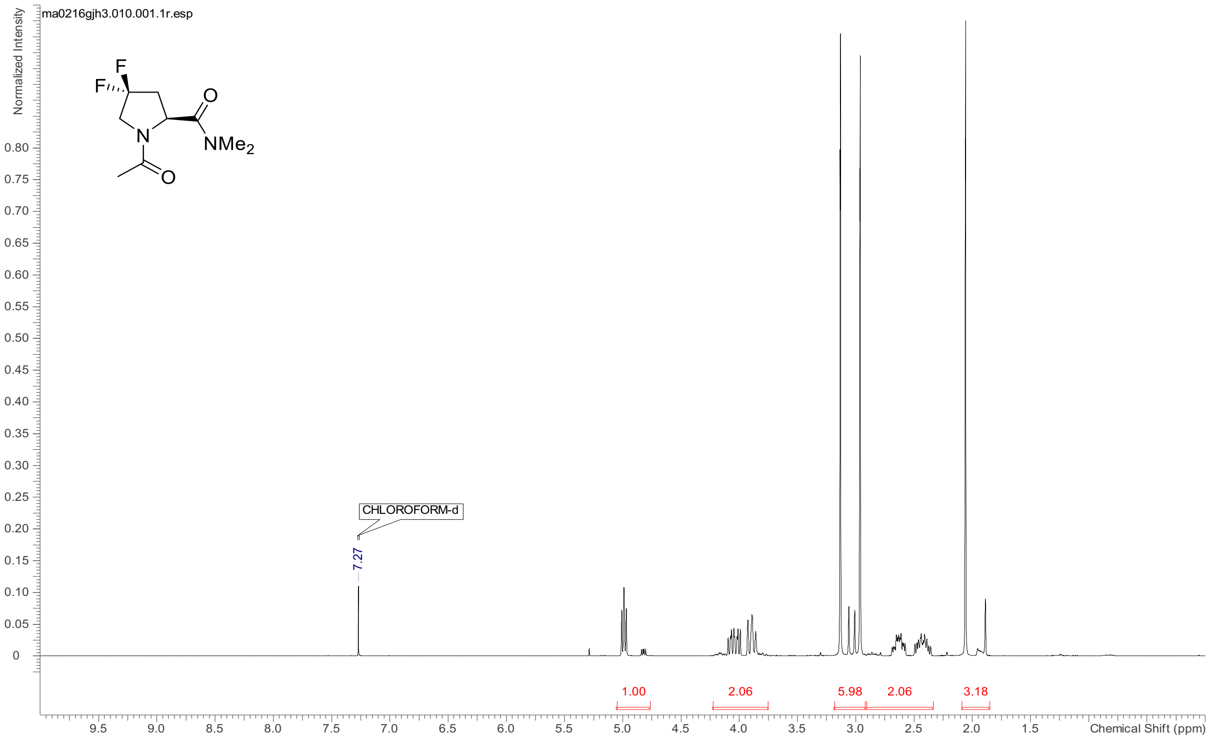

9.18 *N*-(Acetyl)-(2*S*)-4,4-difluoroproline dimethyl amide **7b**: $^{13}C\{^{1}H\}$ NMR spectrum ($CDCl_3$, 100 MHz)

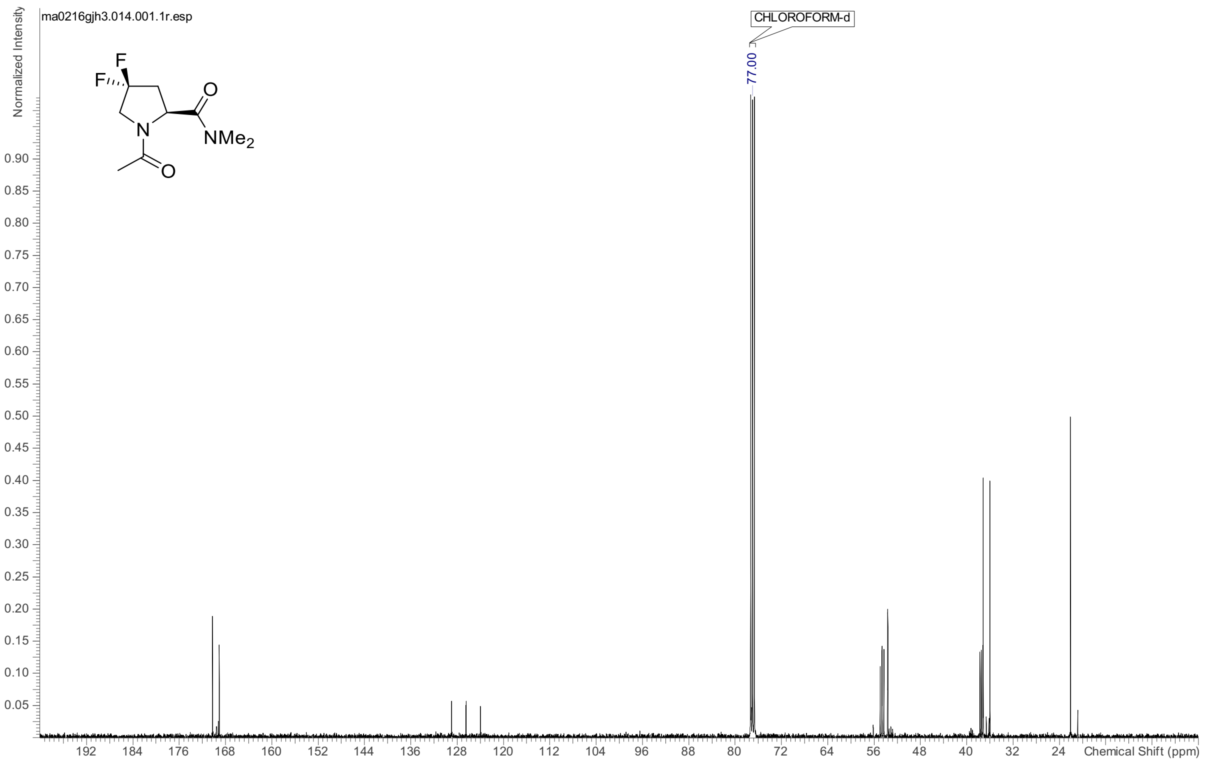

9.19 *N*-(Acetyl)-(2*S*)-4,4-difluoroproline dimethyl amide **7b**: ^{19}F NMR spectrum ($CDCl_3$, 471 MHz)

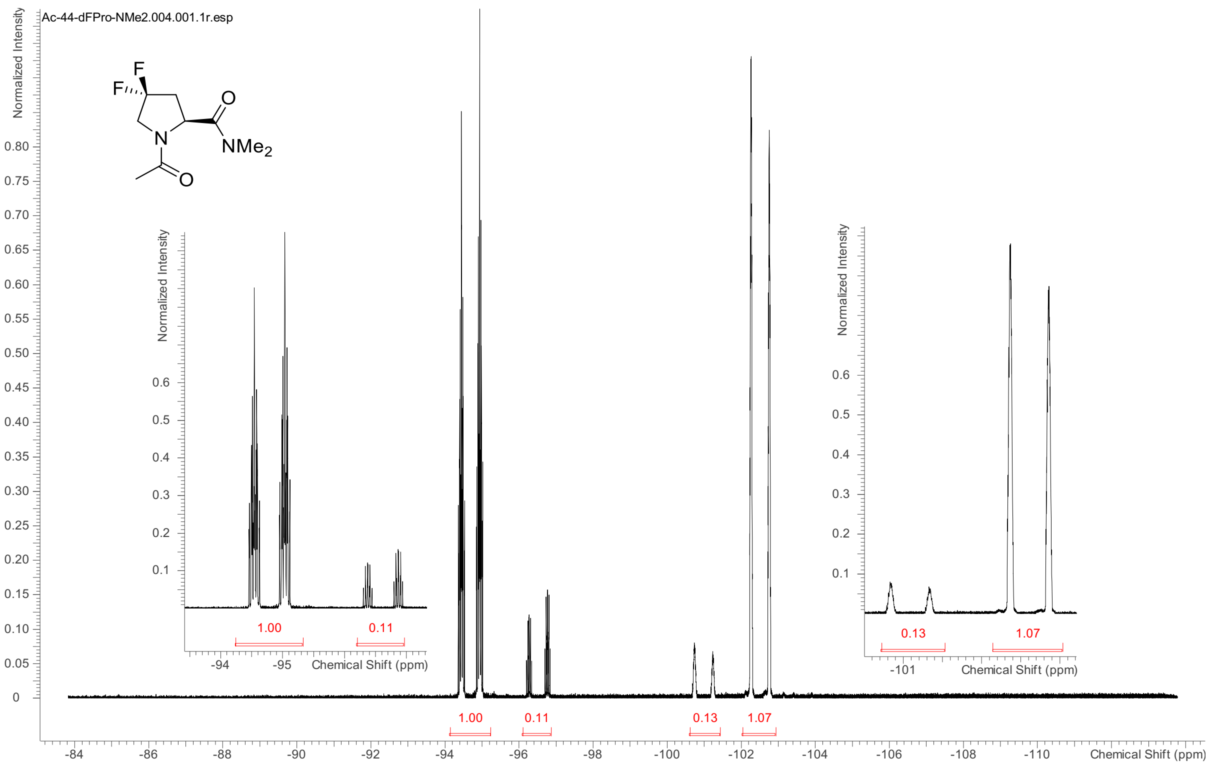

9.20 *N*-(Acetyl)-(2*S*)-4,4-difluoroproline dimethyl amide **7b**: $^{19}F\{^1H\}$ NMR spectrum ($CDCl_3$, 471 MHz)

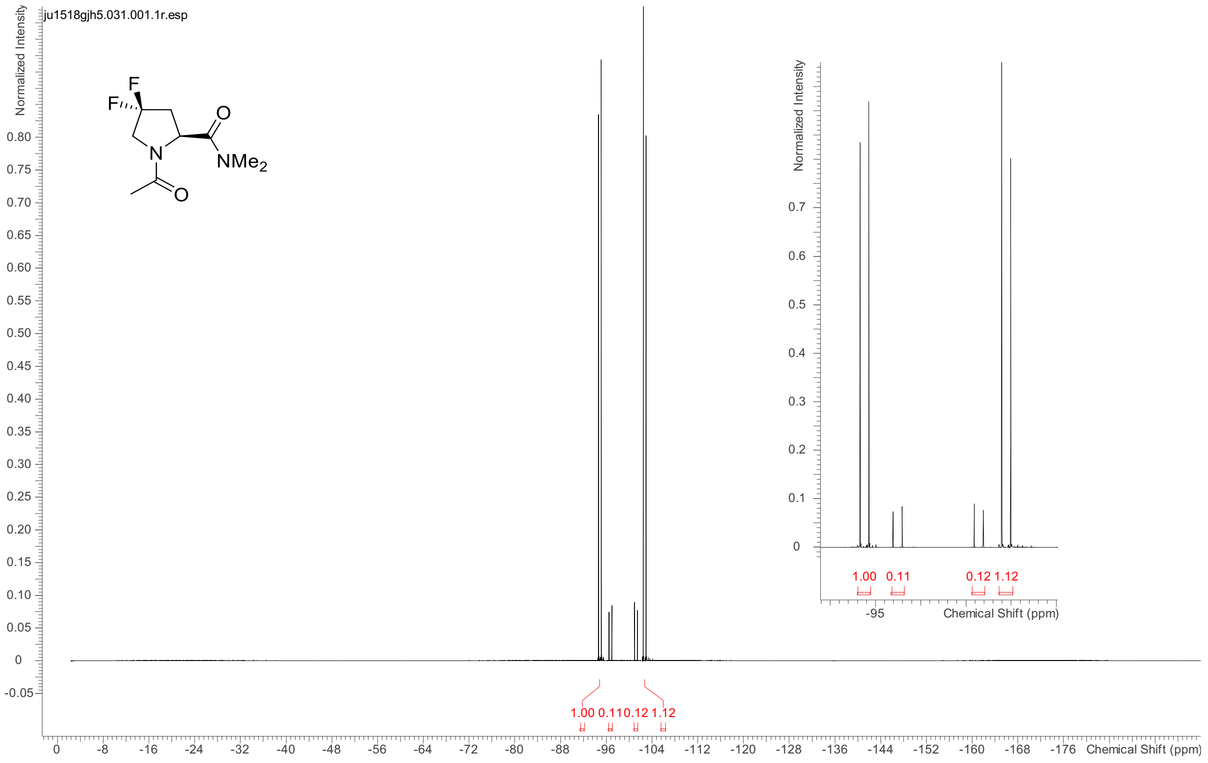

10 References

[1] B. Linclau, Z. Wang, G. Compain, V. Paumelle, C. Q. Fontenelle, N. Wells, A. Weymouth-Wilson, *Angew. Chem. Int. Ed.* **2016**, *55*, 674-678.

[2] M. J. Frisch, G. W. Trucks, H. B. Schlegel, G. E. Scuseria, M. A. Robb, J. R. Cheeseman, G. Scalmani, V. Barone, G. A. Petersson, H. Nakatsuji, X. Li, M. Caricato, A. V. Marenich, J. Bloino, B. G. Janesko, R. Gomperts, B. Mennucci, H. P. Hratchian, J. V. Ortiz, A. F. Izmaylov, J. L. Sonnenberg, Williams, F. Ding, F. Lipparini, F. Egidi, J. Goings, B. Peng, A. Petrone, T. Henderson, D. Ranasinghe, V. G. Zakrzewski, J. Gao, N. Rega, G. Zheng, W. Liang, M. Hada, M. Ehara, K. Toyota, R. Fukuda, J. Hasegawa, M. Ishida, T. Nakajima, Y. Honda, O. Kitao, H. Nakai, T. Vreven, K. Throssell, J. A. Montgomery Jr., J. E. Peralta, F. Ogliaro, M. J. Bearpark, J. J. Heyd, E. N. Brothers, K. N. Kudin, V. N. Staroverov, T. A. Keith, R. Kobayashi, J. Normand, K. Raghavachari, A. P. Rendell, J. C. Burant, S. S. Iyengar, J. Tomasi, M. Cossi, J. M. Millam, M. Klene, C. Adamo, R. Cammi, J. W. Ochterski, R. L. Martin, K. Morokuma, O. Farkas, J. B. Foresman, D. J. Fox, Wallingford, CT, **2016**.

[3] aH. S. Yu, X. He, D. G. Truhlar, *J. Chem. Theory Comput.* **2016**, *12*, 1280-1293; bH. Y. S. Yu, X. He, S. H. L. Li, D. G. Truhlar, *Chem. Sci.* **2016**, *7*, 6278-6279.

[4] A. V. Marenich, C. J. Cramer, D. G. Truhlar, *J. Phys. Chem. B* **2009**, *113*, 6378-6396.

[5] P. Bhar, D. W. Reed, P. S. Covello, P. H. Buist, *Angew. Chem. Int. Ed.* **2012**, *51*, 6686-6690.

[6] M. Hudlicky, *J. Fluorine Chem.* **1993**, *60*, 193-210.

[7] N. Panasik, E. S. Eberhardt, A. S. Edison, D. R. Powell, R. T. Raines, *Int. J. Pept. Protein Res.* **1994**, *44*, 262-269.

[8] C. Siebler, B. Maryasin, M. Kuemin, R. S. Erdmann, C. Rigling, C. Grünenfelder, C. Ochsenfeld, H. Wennemers, *Chem. Sci.* **2015**, *6*, 6725-6730.

[9] M. D. Shoulders, K. J. Kamer, R. T. Raines, *Bioorg. Med. Chem. Lett.* **2009**, *19*, 3859-3862.

[10] aL. Demange, A. Ménez, C. Dugave, *Tetrahedron Lett.* **1998**, *39*, 1169-1172; bJ. Chiba, G. Takayama, T. Takashi, M. Yokoyama, A. Nakayama, J. J. Baldwin, E. McDonald, K. J. Moriarty, C. R. Sarko, K. W. Saionz, R. Swanson, Z. Hussain, A. Wong, N. Machinaga, *Biorg. Med. Chem.* **2006**, *14*, 2725-2746.
